# Reactive machine learning potential for accelerating transition state search in organic synthesis

**DOI:** 10.1038/s41467-026-72945-0

**Published:** 2026-05-08

**Authors:** Kaipai Ren, Kun Tang, Yujing Zhao, Lei Zhang, Jian Du, Qingwei Meng, Qilei Liu

**Affiliations:** 1 State Key Laboratory of Fine Chemicals, Frontiers Science Center for Smart Materials Oriented Chemical Engineering, Department of Pharmaceutical Sciences, Institute of Chemical Process Systems Engineering, School of Chemical Engineering, Dalian University of Technology, Dalian, China; 2 MOE Key Laboratory of Bio-Intelligent Manufacturing, School of Bioengineering, Dalian University of Technology, Dalian, China; 3 Ningbo Institute of Dalian University of Technology, Ningbo, China

**Keywords:** Method development, Density functional theory, Reaction mechanisms

## Abstract

Understanding reaction kinetics is fundamental to organic synthesis, yet traditional quantum chemistry-based transition state searches are computationally expensive. Here we present DeePEST-OS, a reactive machine learning potential designed for rapid and accurate transition state optimization and energy barrier prediction spanning ten chemical elements. Trained on approximately 75,000 reactions generated by a low-cost data preparation strategy, this model integrates physical priors from semi-empirical quantum chemistry with equivariant message passing networks to predict potential energy surfaces nearly 10,000 times faster than quantum chemistry methods, while achieving high accuracy for transition state geometry (averaged root mean square deviation of 0.12 Å) and energy barriers (mean absolute error of 0.60 kcal/mol) on unseen reactions. DeePEST-OS enables practical applications including transition state conformer screening, barrier prediction for retrosynthesis of complex pharmaceuticals, and experimentally validated diastereoselectivity prediction in Diels-Alder reactions. Collectively, these results establish DeePEST-OS as a powerful tool for accelerating reaction kinetics studies in multi-element organic synthesis.

## Abstract (web summary)

Reaction kinetics is fundamental to organic synthesis. Here the authors build a 10-element reaction database and develop a reactive machine learning potential to accelerate transition state search and reaction barrier prediction.

## Introduction

Organic synthesis is a fundamental aspect of modern chemistry, enabling the creation of a wide variety of molecules with essential applications in medicine, materials science, and so on^1–3^. The role of reaction kinetics in organic synthesis is crucial, as it helps explain reaction rates, predict their dependence on reaction conditions, and thereby optimize conditions for greater efficiency. Central to reaction kinetics is transition state theory^4^, which describes the high-energy, transient configuration of atoms that occurs during a reaction. Through the analysis of the transition state, researchers gain valuable insights into the factors that govern reaction rates, including the energy barrier and the influence of molecular interactions and reaction conditions on the overall reaction pathway^5^.

Although theoretically significant, capturing the intermediates and transition states involved in elementary reactions remains experimentally challenging. As a result, the study of reaction kinetics primarily relies on Quantum Chemistry (QC) calculations. Density Functional Theory (DFT)^6^ is widely applied in reaction kinetics research, but its calculations demand costly energy, gradients, and Hessian information to optimize transition states and intermediate structures, presenting challenges for high-throughput implementations. This constraint impedes large-scale exploration and screening of reaction mechanisms. Another increasingly popular approach in recent years is the use of semi-empirical QC methods (e.g., GFN-xTB)^7^ or approximate QC methods (e.g., DFTB)^8^ for the geometry optimization of transition states and intermediates. Although these methods are typically several orders of magnitude faster than DFT calculations, this approach tends to struggle in providing precise structures or accurate energy values, meaning that the predicted reaction barriers are likely to be untrustworthy.

In light of this, there is a growing need for methods that combine the accuracy of DFT calculations with the efficiency of semi-empirical approaches, in order to advance reaction kinetics research more effectively. Machine Learning (ML) has emerged as a promising solution, capable of achieving high-precision predictions of reaction rates and mechanisms by rapidly processing large datasets, while reducing computational costs^9–12^. Furthermore, as more data becomes available, ML models can be continuously refined, improving their predictive accuracy and reliability, and thereby enabling reaction kinetics research to be conducted in a more time-efficient and scalable manner, with the potential to transform the discovery of new reactions and the optimization of synthetic strategies. Currently, end-to-end prediction methods represent a relatively mature application of ML in the field of reaction kinetics. For instance, in 2022, William H. Green and colleagues^13^ developed a directed message passing neural network to predict barrier heights more accurately than DFT, while in 2024, Green and Maike Bergeler^14^ trained a deep graph network to predict Gibbs free energy barriers in both gas and solution phases, all from atom-mapped Simplified Molecular Input Line Entry System (SMILES). These end-to-end methods, due to their circumvention of transition state search for predicting reaction-related properties, are limited in providing comprehensive and detailed understanding of reaction mechanisms, consequently demonstrating inferior performance compared to standard benchmarks on external test sets^15^. In 2025, Duan et al. achieved a breakthrough in ML-based prediction of transition state structures, developing the React-OT model for organic reactions based on optimal transport model^16^. This model enabled high-throughput generation of transition state structures with relatively high accuracy based on the structures of reactants and products. However, its applicability and accuracy are constrained by the benchmark dataset (Transition1x)^17^, which exclusively contains four elements (C, H, O, N), and its DFT level (ωB97x/6-31 G(d)) has relatively low accuracy, limiting its applicability.

In contrast to end-to-end ML methods that directly generate molecular geometries without considering the Potential Energy Surface (PES), Machine Learning Potential (MLP) models the functional relationship between atomic coordinates and molecular energy/force through ML algorithms, thereby providing enhanced interpretability. This approach eliminates the need for explicit solutions to the Schrödinger equation, enabling the efficient construction of PES and advancing the acceleration of QC calculations. A landmark development in this field was the work of Behler and Parrinello in 2006^18^, who introduced the high-dimensional neural network model, utilizing Atom-Centered Symmetry Functions (ACSFs) as descriptors to ensure translational, rotational, and permutation invariance for many-atom systems of arbitrary size. Since then, descriptor-based MLP models similar to this approach have undergone substantial advancements^19^. In contrast, although the application of graph neural networks to predict the PES of molecular systems is relatively recent, it has rapidly advanced, with models like PaiNN^20^, SchNet^21^, NequIP^22^, AIMNet^23^, SpookyNet^24^, and MACE^25^ achieving high accuracy across various benchmark datasets^22,25–32^. The MLP model architecture has progressively advanced from invariance to high-order equivariance, with recent developments extending into more refined domains, including the optimization of atomic cluster expansion and neural equivariant interatomic potentials^33^.

While MLP models have been widely used in molecular dynamics simulations^34^ and geometry optimizations^35^, their application in transition state search and reaction kinetics prediction remains limited. In 2025, Zhao et al. ^36^ established an end-to-end workflow for transition state search to benchmark seven MLP models against the React-OT generative model. The results demonstrated that React-OT outperformed MLP-based transition state search, achieving a higher success rate in locating transition states. The poor performance of these MLP models may be attributed to their lack of training on transition state structures and reactive PES. A major challenge is that transition states, as first-order saddle points on high-dimensional PES, require dynamic evolution of reaction coordinates and higher precision for potential energy gradient than stable molecules with minimal energy on PES. Another critical limitation is that the available datasets for training MLP models in organic molecular systems predominantly consist of equilibrium or near-equilibrium configurations^37^, while large-scale databases and modeling efforts focused on transition states remain comparatively scarce. In 2020, William H. Green et al. used automated PES exploration to generate 12,000 organic reactions involving H, C, N, and O atoms calculated at the ωB97X-D3/def2-TZVP theory level^38^. In 2022, Mathias Schreiner et al. constructed the Transition1x dataset by applying the climbing image nudged elastic band method to 10,073 types of organic reactions, generating forces and energies for 9.6 million molecular configurations along reaction pathways at the ωB97x/6-31 G(d) theory level^17^. In 2023, Zhao et al. developed the RGD1 database based on reaction network exploration^39^. RGD1 encompasses 176,992 organic reactions, featuring C, H, O, and N-containing molecules with up to 10 heavy atoms^40^. However, as MLP studies of organic reactions expand to more complex systems, such as drug molecules with sulfur or halogens, the limitations of current databases in element types have become increasingly evident. In order to advance the application of ML in organic reaction prediction, it is imperative to construct a reaction database that encompasses a broader range of atomic types and more diverse reaction types^41,42^.

Two pivotal contributions are presented in this work to address the limitations in existing studies. First, a hybrid data preparation strategy is developed to construct a large-scale Database of Organic Reaction Transition States (DORTS) in a cost-effective manner. Compared to the Transition1x dataset, DORTS provides substantially broader chemical diversity in organic reactions. Second, DeePEST-OS (Deep learning-based molecular Potential Energy Surface prediction Tool for Organic Synthesis) is proposed by integrating prior physical knowledge from GFN2-xTB into the MACE architecture via a Δ-learning strategy^43^, enabling accurate predictions of transition state geometries and energy barriers with robust extrapolation capabilities across diverse reaction scenarios. Compared to DFT calculations, DeePEST-OS achieves comparable accuracy while accelerating transition state optimization by four orders of magnitude. Additionally, it provides accurate reaction barrier predictions that are highly consistent with rigorous DFT methods, outperforming semi-empirical QC methods (e.g., GFN2-xTB). DeePEST-OS offers an efficient alternative to DFT for accurate transition state optimizations, transition state conformer evaluations and reaction barrier predictions in organic synthesis, facilitating high-throughput explorations of complex reaction networks.

## Results

### Workflow of developing DeePEST-OS

**Fig. 1:**
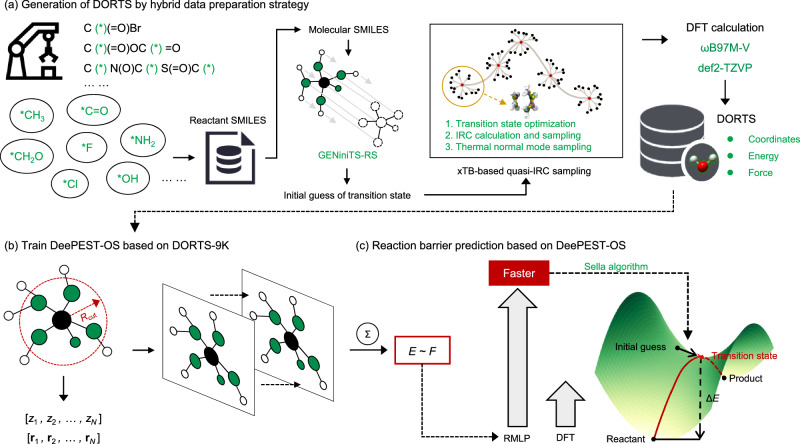
Workflow of developing DeePEST-OS. **a** Generation of the DORTS database using a hybrid data preparation strategy. **b** Training DeePEST-OS on the DORTS-9K database. **c** Prediction of reaction energy barriers using DeePEST-OS.

The workflow of developing DeePEST-OS comprises three key stages: (1) Establish the DORTS database, (2) Construct the DeePEST-OS, and (3) Use DeePEST-OS to predict reaction barriers, as shown in Fig. 1. In the first step (Fig. 1(a)), DORTS is constructed using a hybrid data preparation strategy that automatically and efficiently generates approximately 7.5 million molecular conformations along 74,837 organic reaction pathways, together with the corresponding molecular energies and atomic forces. In the second step (Fig. 1(b)), DORTS-9K, a subset of DORTS containing 9,017 reactions, is randomly selected and employed to train DeePEST-OS using the Δ-learning strategy within the MACE architecture, enabling high-precision and high-efficiency predictions of molecular energies and atomic forces. In the third step (Fig. 1(c)), DeePEST-OS is integrated with transition state optimization algorithms (e.g., Sella)^44^ to achieve high-throughput transition state searches and reaction barrier predictions.

### Generation of the DORTS database

Organic reactions exhibit high diversity in both variety and quantity. A thorough investigation of any single organic reaction network may yield tens of thousands of distinct reactions. The sheer volume of these chemical transformations renders the establishment of a comprehensive reaction database through network exploration impractical. Moreover, such enumeration tends to generate a large number of low-value reactions, which dilute the overall quality of the dataset. As a result, the predictive performance of MLP models on high-value or frequently studied reactions can be severely compromised. To address these challenges, a hybrid data preparation strategy was employed to generate a comprehensive reaction database. This strategy consisted of four key components: reaction template acquisition, reaction generation, xTB-based quasi-IRC sampling, and DFT calculations of molecular energies and atomic forces.

First, reaction templates were acquired. March’s Advanced Organic Chemistry^45^ covers a wide range of topics in organic chemistry and systematically expounds on various organic reactions and their mechanisms. In this study, 255 fundamental reactions were extracted from this book and encoded as the SMARTS (SMiles ARbitrary Target Specification)-based reaction templates for constructing our DORTS database. These reactions exhibit high significance and strong mechanistic coverage while maintaining elemental diversity across ten main-group elements (C, H, O, N, P, S, F, Cl, Br, I). The reaction types include substitution, addition, ring-opening/closing process, and molecular rearrangement, with the reaction templates provided in Suppl. Table 1.

**Fig. 2:**
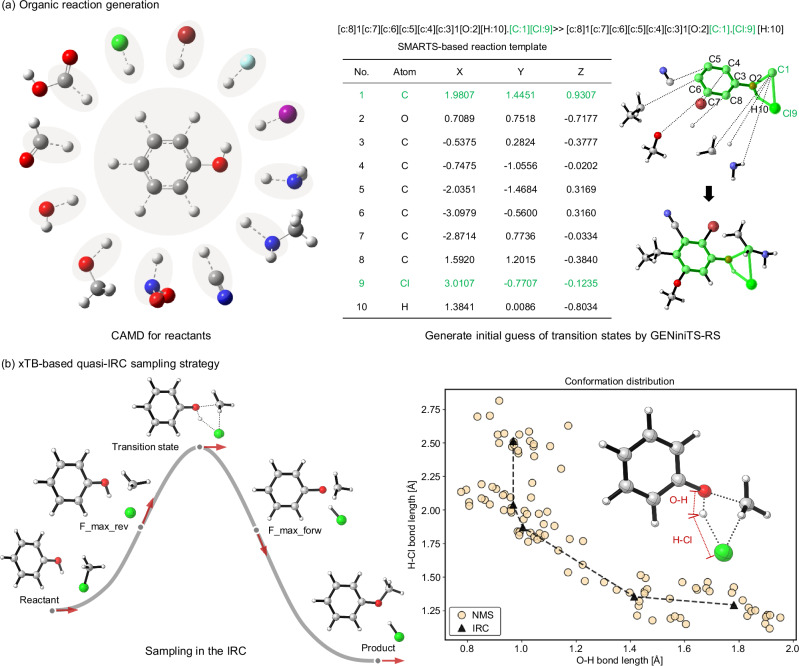
The generation of the DORTS database. **a** The scaffold-based CAMD method and the GENiniTS-RS algorithm used to generate initial guesses of transition states. **b** Five selected critical conformations along the IRC pathway (left figure) and the NMS-sampled conformations (yellow dots) for the five selected critical conformations (black dots) along the IRC pathway during phenol etherification (right figure). Source data are provided as a Source Data file.

Second, organic reactions were generated. Within the realm of organic chemistry, substituent groups play a pivotal role in modulating reaction reactivity, kinetics, selectivity, and stability through diverse mechanisms, including electronic, steric, and resonance effects. To investigate substituent effects, a scaffold-based Computer-Aided Molecular Design (CAMD) method^46^ was employed to generate a variety of SMILES-represented reactants and corresponding reactions based on reaction templates. Specifically, the CAMD-based reactant design problem was formulated as a Mixed-Integer Linear Programming (MILP) model and solved using the BARON solver^47^, enabling substituent modifications of reactants across 255 templates and generating approximately 90,000 SMILES-encoded reactions (Fig. 2(a)). Afterwards, the GENiniTS-RS algorithm^48^ was implemented to automatically detect the reactive sites in SMILES-encoded reactions and performed high-throughput generations of corresponding ~90,000 initial guesses of transition states via reaction template matching. This systematic approach provides robust structured data support for developing comprehensive reaction databases, effectively bridging computational predictions with chemically meaningful molecular transformations.

Third, an xTB-based quasi-IRC sampling strategy was developed. The advancement of MLP models over conventional DFT calculations for predicting reaction barriers hinges on the systematic learning of interatomic potential evolution across reaction pathways. When trained on databases containing sufficient atomic environments analogous to those in novel compounds, MLP models exhibit reliable extrapolation capabilities to new chemical systems. This underscores the necessity of constructing databases encompassing transition states and molecular conformations along IRC pathways, as critically emphasized by Schreiner et al. ^17^. To achieve this objective, an xTB-based quasi-IRC sampling strategy (Fig. 2(b)) was developed to enable full-coverage and low-cost sampling of complete reaction pathways. First, the geometric conformations of all initial guesses of transition states were optimized using the efficient GFN2-xTB method, accompanied by IRC path analysis at the same computational level^49^. Any structures that failed IRC validation were discarded. This process successfully generated 74,837 optimized transition states with converged IRC pathways. Then, five critical conformations were selected along the IRC pathway. The two endpoints represented the reactant and product structures, and the maximum point corresponded to the transition state. The remaining two conformations were identified as those with the steepest slopes along the IRC pathway—specifically, the points where the absolute value of the first derivative of energy with respect to the reaction coordinate reaches its maximum. Since this first derivative represents the force acting on the system, these two conformations were denoted as F_max_rev (reverse direction) and F_max_forw (forward direction). Finally, the quantum thermal Normal Mode Sampling (NMS)^50^ method was implemented at these five critical conformations (transition state, reactant, product, F_max_rev and F_max_forw) along each IRC path. This enabled enhanced sampling of molecular conformations near the IRC, facilitated MLP-driven transition state optimizations. For illustration, Fig. 2(b) demonstrates this approach using phenol etherification, where the reaction pathway is monitored via O-H and H-Cl bond length variations. The sampling methodology exhibits robust coverage of the reaction coordinate space, evidenced by continuous geometry progression visualization. This NMS sampling method was subsequently applied to all 74,837 confirmed IRC paths, generated approximately 7.5 million distinct conformations that constituted the comprehensive DORTS database.

Finally, DFT calculations of molecular energies and atomic forces were performed. The optimization of transition states using MLP models fundamentally relies on the accurate prediction of molecular energies and atomic forces. DFT has been widely adopted for calculating these properties. The selection of functionals and basis sets significantly impacts the precision of final results. In 2016, Mardirossian et al. developed the ωB97M-V hybrid functional^51^ and systematically evaluated its performance against MP2, four hybrid functionals, and nine double-hybrid functionals using the MainGroup Chemistry DataBase (MGCDB84)^6^. Through comprehensive benchmarking metrics^52^, including non-covalent interactions (NCED, NCEC, NCD), isomerization energies (IE, ID), thermochemistry (TCE, TCD), and barrier heights (BH), ωB97M-V demonstrated superior accuracy for main-group element calculations. Consequently, to balance computational cost and model accuracy, this study randomly selected 9,017 reactions from the DORTS database (Supplementary Fig. 1 illustrates the distribution of reaction counts for DORTS-9K and DORTS across 255 reaction templates), from which 874,176 molecular conformations were generated. High-precision DFT calculations employing the ωB97M-V functional and the def2-TZVP basis set were conducted to obtain molecular energies and atomic forces, resulting in the DORTS-9K dataset, which provides a robust foundation for training DeePEST-OS in transition state optimization and reaction mechanism elucidation.

**Fig. 3:**
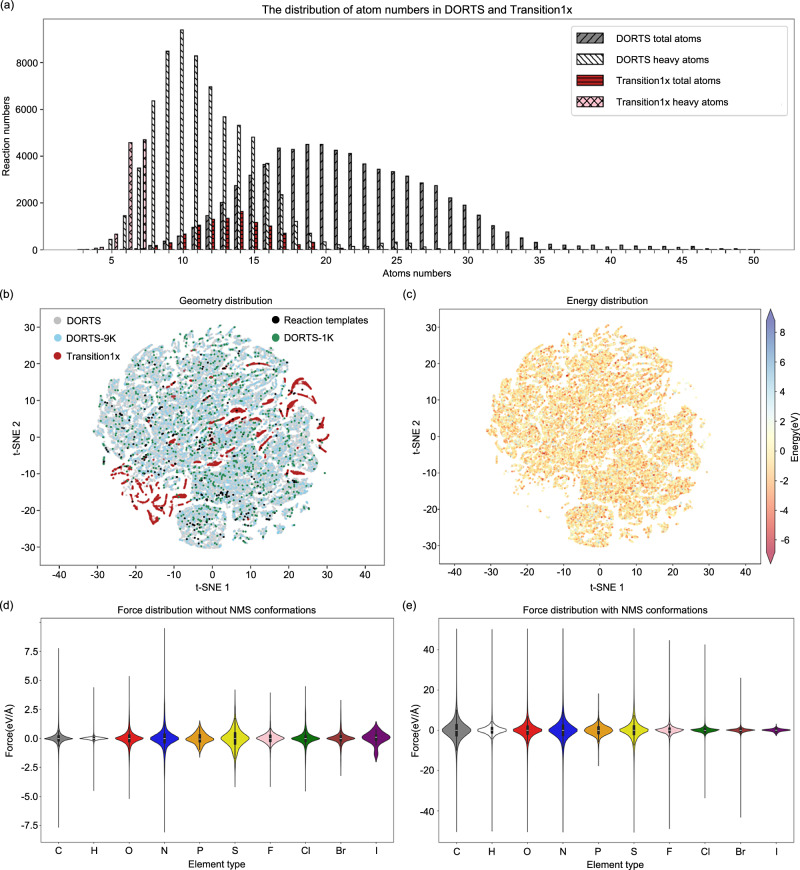
Data analysis for the DORTS database. **a** Distribution of total atoms and heavy atoms per reaction in the DORTS and Transition1x databases (gray bars represent total atom counts in DORTS; white bars represent heavy atom counts in DORTS; red bars represent total atom counts in Transition1x; pink bars represent heavy atom counts in Transition1x). **b** Chemical space mapped using the ACSFs descriptors and t-SNE method (gray points represent the DORTS transition states; blue points represent the DORTS-9K transition states that are used to train DeePEST-OS; red points represent the Transition1x transition states; black points represent the initial 255 reaction templates; green points represent DORTS-1K comprising 1,000 reactions not included in DORTS-9K). **c** Chemical space of the DORTS transition states with colors representing energy values (the plotted energy values are obtained by subtracting the reference energies from the DFT-calculated energies). **d** Violin diagram of atomic force distributions considering only IRC conformations. **e** Violin diagram of atomic force distributions considering both IRC and NMS conformations. Source data are provided as a Source Data file.

The DORTS database contained molecular structures with total atom counts ranging from 6 to 50 and heavy atom counts spanning 3 to 30 across individual reactions. Comparative analysis of atomic distributions between the DORTS and Transition1x databases^17^ (Fig. 3(a)) reveals that reactions in DORTS exhibited greater structural complexity, as evidenced by broader ranges of both total and heavy atom counts per reaction, along with increased data volume. Elemental composition analysis showed distinct representation percentages for each atom type: carbon (C, 29.7%), hydrogen (H, 45.9%), oxygen (O, 14.3%), nitrogen (N, 6.2%), phosphorus (P, 0.1%), sulfur (S, 1.1%), fluorine (F, 0.6%), chlorine (Cl, 1.4%), bromine (Br, 0.8%), and iodine (I, 0.01%). Structural diversity among the 255 reaction templates and their corresponding 74,837 transition states is visualized through a chemical space mapped by ACSFs descriptors^19^ and the t-distributed Stochastic Neighbor Embedding (t-SNE) method (Fig. 3(b)), where each point corresponds to a 3D molecular conformation and geometric proximity between points reflects structural similarity. Overall, the 74,837 reactions exhibited uniform spatial distribution across chemical domains, though minor sparsity patterns were observed in boundary regions. Complementary visualization of energy-annotated chemical space was subsequently performed (Fig. 3(c)), utilizing ACSFs descriptors and t-SNE projection with energy values normalized by subtraction of reference energies from DFT-computed values. The resultant energy landscape demonstrated uniform continuity with aligned energy progression gradients, indicating that DORTS possessed great energy span characteristics conducive to the training of DeePEST-OS. Reference energy baselines for individual atomic types are tabulated in supporting information Suppl. Table 2. Comparative spatial distribution analysis between the DORTS and Transition1x databases (Fig. 3(b)) reveals that DORTS exhibited superior chemical space coverage both in spatial extent and uniformity. Other comparisons between the DORTS and Transition1x databases, including element types, reaction types, number of reactions, and calculation level, are summarized in Table 1.

**Table 1:** Comparison of the DORTS database and Transition1x database

Database	DORTS	Transition1x
Element types	C, H, O, N, P, S, F, Cl, Br, I	C, H, O, N
Reaction types	A → C A → C + D A + B → C A + B → C + D	A → C A → C + D A + B → C
Number of reactions	74,837	10,073
Calculation level	High level: ωB97M-V/def2-TZVP	Low level: ωB97x/6-31 G(d)

Previous studies have demonstrated that a more extensive force distribution dataset is critical for training MLP models^32^. Analysis of DORTS conformations without NMS-enhanced geometries demonstrates that optimized transition states exhibited near-zero atomic forces (Fig. 3(d)). This finding indicates that DeePEST-OS trained on such restricted datasets faced substantial limitations in transition state optimization^53^, necessitating the inclusion of NMS-generated conformations in training data composition. Figure 3(e) demonstrates that datasets with NMS-sampled conformations showed broader atomic force distributions across element types compared to their IRC-only counterparts. These characteristics suggest that the hybrid data preparation strategy (xTB-based quasi-IRC sampling combined with high-precision DFT molecular energies and atomic force calculations) served as a cost-effective alternative to fully DFT-based data preparation strategies, the effectiveness of which was confirmed by the model training results and the successful optimizations of transition states on the external test set in the following sections.

The proposed hybrid data preparation strategy reduces the computational costs associated with sampling conformations. Comparative analyses of the computational costs for phenol etherification were conducted on a laptop computer (AMD Ryzen 9 7945HX, 48GB RAM). When utilizing the hybrid data preparation strategy, the GFN2-xTB method took 0.3, 13.6, and 4.2 core·seconds (core·s) to perform the tasks of transition state search, IRC path search, and NMS calculations, respectively. If the hybrid strategy was not used, the DFT method (ωB97M-V functional and def2-TZVP basis set) necessitated 19,381, 82,058, and 80,594 core·s to execute the transition state search, IRC path search, and NMS calculations, respectively. In summary, the total time required for conformation sampling in this example, with and without the hybrid data preparation strategy, was 18.1 and 182,033 core·s, respectively. The inherent computational efficiency of GFN2-xTB itself enabled the time with the strategy to account for approximately 0.01% of that without the strategy, highlighting the efficiency of this hybrid data preparation strategy.

### Accuracy and efficiency of DeePEST-OS

The DORTS-9K subset, comprising 874,176 NMS conformations in total, was used to train DeePEST-OS and randomly split into training, validation, and test sets at an 8:1:1 ratio. The DFT-calculated molecular energies and atomic forces served as the benchmark for evaluations of the trained DeePEST-OS. In this work, the MACE architecture was employed to construct DeePEST-OS. This architecture achieves high-order tensor message passing (up to four-body interactions) through a differentiable atomic cluster expansion framework, demonstrating high accuracy and data efficiency in molecular energy and atomic force prediction within the current MLP field^25^. To systematically evaluate the impact of model architecture on prediction performance, the PaiNN architecture was also selected as a comparative benchmark. This architecture, based on a message passing mechanism of spherical harmonics Taylor expansion, offers computational efficiency advantages but has relatively limited accuracy^20^. Notably, this study introduced a Δ-learning strategy^43^, coupling the mechanism-driven semi-empirical QC methods (GFN2-xTB) with the data-driven MACE architecture. The former, based on parameterization methods with physical approximations (such as the accurate and broadly parametrized self-consistent tight-binding framework), possesses theoretical self-consistency in scenarios involving electron delocalization, weak interactions, and long-range interactions. The latter captures the fine-grained features of the local chemical environment through DFT data-driven high-order tensor learning. This cross-paradigm fusion overcomes the limitations of single modeling approaches: the theoretically prior knowledge from GFN2-xTB enhances the model extrapolation robustness in data-sparse regions, while the data-driven characteristics of MACE accurately reproduce DFT-level PES details.

**Fig. 4:**
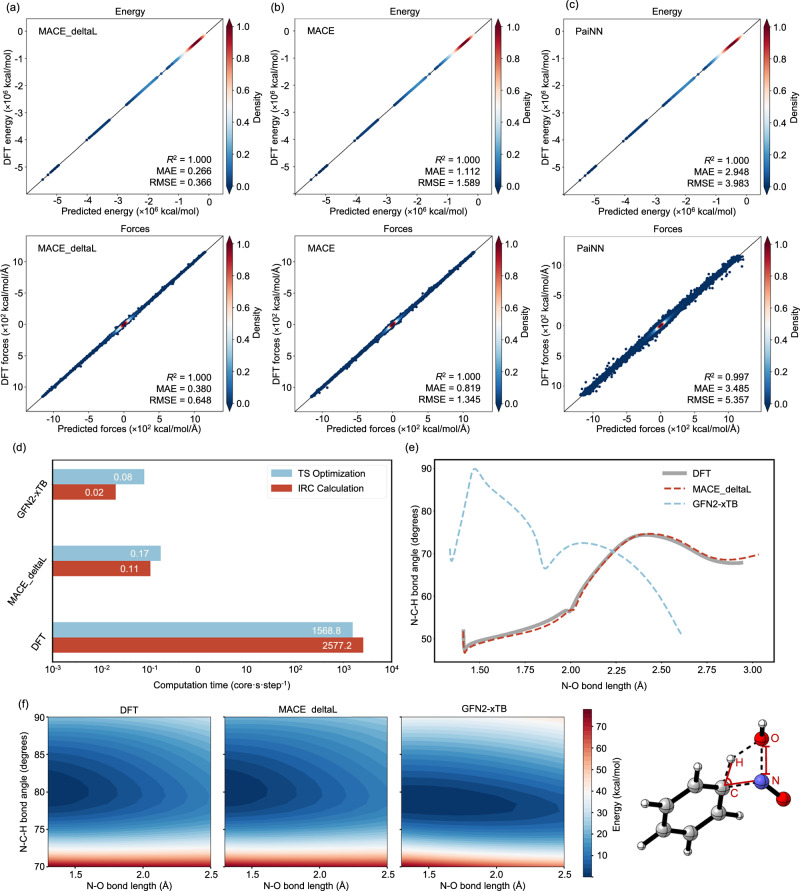
Model training results and comparisons. **a** Performance of the MACE_deltaL model. **b** Performance of the MACE model. **c** Performance of the PaiNN model. **d** Comparison of the time cost by different methods (the blue and red bars denote TS optimization and IRC calculation, respectively). **e** Comparison of the reaction coordinates for N-O bond stretching and N-C-H angle bending along the IRC path calculated by different methods (the grey line, red line, and blue line correspond to DFT, MACE_deltaL, and GFN2-xTB, respectively). **f** Comparison of the PES scanning at the reaction coordinates for N-O bond stretching and N-C-H angle bending calculated by different methods. Source data are provided as a Source Data file.

The systematic comparison in Fig. 4(a-c) reveals the performance differences among various MLP architectures. The PaiNN architecture exhibited significant accuracy limitations on the test set, with its Mean Absolute Errors (MAE) for molecular energy and atomic force predictions (2.948 kcal/mol and 3.485 kcal/mol/Å, respectively) and Root Mean Square Errors (RMSE) (3.983 kcal/mol and 5.357 kcal/mol/Å, respectively) being substantially larger than those of the MACE architecture (Fig. 4(a)). This discrepancy stems from the insufficient representation of high-order chemical interactions by its message passing mechanism. The pure MACE architecture demonstrated a significant improvement, with the MAE for energy and forces decreasing to 1.112 kcal/mol and 0.819 kcal/mol/Å, respectively (Fig. 4(b)). This is attributed to its differentiable four-body cluster expansion framework capability for explicit modeling of complex interactions such as bond angle torsions and π-π stacking. Most importantly, the MACE_deltaL architecture, which incorporates the Δ-learning strategy based on the GFN2-xTB method, further enhanced the energy prediction accuracy to an MAE of 0.266 kcal/mol and an RMSE of 0.366 kcal/mol, as well as the atomic force prediction accuracy to an MAE of 0.380 kcal/mol/Å and an RMSE of 0.648 kcal/mol/Å (Fig. 4(c)). This breakthrough stems from the generalizability advantage of GFN2-xTB: this semi-empirical QC method self-consistently incorporates the atomic partial charge-dependent D4 London dispersion model, derived naturally in a tight-binding picture from second-order density fluctuations. The result is universal capability in describing electronic structures across diverse systems, including organic molecules and non-covalent complexes. The Δ-learning mechanism provides strong generalization: GFN2-xTB stable predictions for scenarios where traditional MLPs tend to fail, such as complex interatomic interactions involved in transition state search, providing MACE with a cross-chemical space correction benchmark. This led to a reduction in the RMSE of PES prediction for MACE during organic reaction pathway extrapolation (especially in the saddle point regions of PES) compared to the pure MACE architecture. This improvement in accuracy reflects the complementarity of data-driven and mechanism-driven paradigms: MACE fits the local fine-grained features of the DFT-calculated PES, while GFN2-xTB ensures the topological rationality of the global PES through physical constraints. The synergy between the two enhances MACE_deltaL predictive reliability for reaction pathways not covered in the training set.

Based on the comparison of the above results, the reaction of benzene with nitrous acid was employed as a representative case to evaluate the efficiency and accuracy of MACE_deltaL (Fig. 4(d-f)). In this study, transition state optimization and IRC calculation were performed by the Sella algorithm^44^. In the transition state search task, the computational time for MACE_deltaL (0.17 core·s·step^−1^) was comparable in magnitude to that of GFN2-xTB (0.08 core·s·step^−1^), representing a 9228-fold acceleration compared to DFT (1568.8 core·s·step^−1^). Particularly in IRC path calculations, MACE_deltaL (0.11 core·s·step^−1^) achieved four orders of magnitude of acceleration compared to DFT (2577.2 core·s·step^−1^) (Fig. 4(d)), demonstrating the promising inference efficiency of MACE_deltaL that couples MACE with GFN2-xTB. Comparative analysis of key reaction coordinates (N-O bond stretching and N-C-H angle changes) demonstrates that the IRC path reconstructed by MACE_deltaL maintained high consistency with DFT results (Fig. 4(e)), and its PES contour curvature closely matched DFT calculations, outperforming the GFN2-xTB method (Fig. 4(f)). This accuracy advantage stems from the MACE architecture high-order tensor message passing mechanism, which accurately models the p-orbital conjugation effects of the nitro-group oxygen and the charge characteristics of the benzene ring π-system during the reaction. This case study indicates that MACE_deltaL successfully achieves a good balance between computational efficiency and prediction accuracy. The transition state search speed based on the Sella algorithm is comparable to the level of semi-empirical methods, while the PES prediction accuracy approaches the DFT benchmark, showcasing a unique advantage of being both fast and good. This characteristic makes it a powerful tool for studying the kinetics of multi-step organic reactions, offering possibilities for the automated exploration of large-scale reaction networks.

**Fig. 5:**
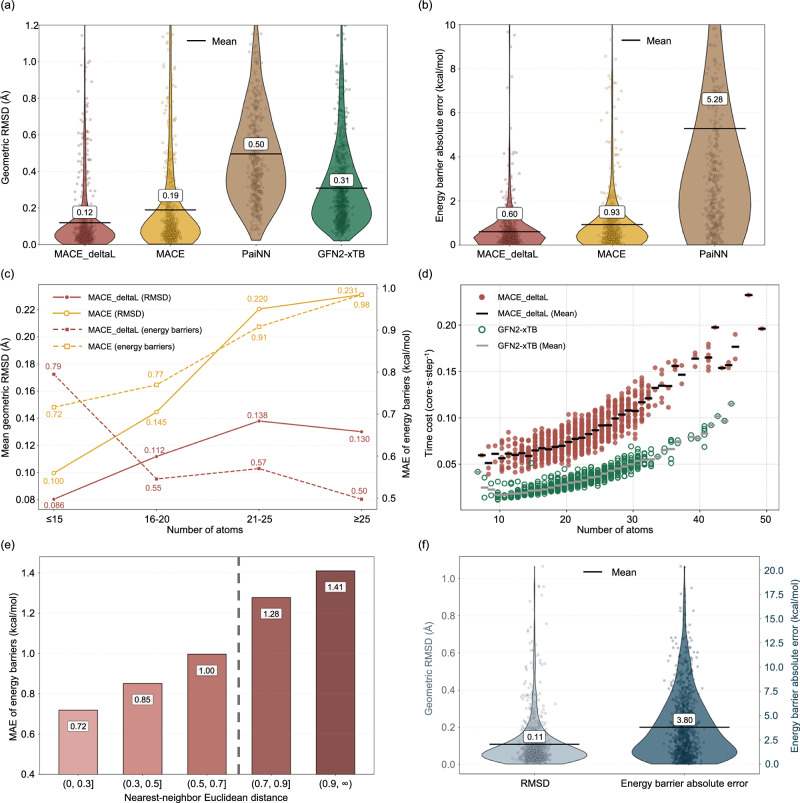
Accuracy and computational cost evaluations based on the test set. **a** Violin plot illustrating the distribution of transition state geometric RMSDs for various methods based on DORTS-1K (red, gold, brown, and green areas denote MACE_deltaL, MACE, PaiNN, and GFN2-xTB, respectively). **b** Violin plot depicting the distribution of absolute errors in predicted reaction energy barriers across different methods based on DORTS-1K (red, gold, and brown areas denote MACE_deltaL, MACE, and PaiNN, respectively). **c** Comparison of MACE and MACE_deltaL prediction errors (mean geometric RMSD and the MAE of reaction energy barriers) on DORTS-1K with respect to the number of atoms (solid lines denote geometric RMSD, while dashed lines denote the MAE of energy barriers; red and gold colors correspond to MACE_deltaL and MACE, respectively). **d** Comparison of computational costs for transition state geometry optimization across systems with different numbers of atoms for GFN2-xTB and MACE_deltaL based on DORTS-1K (red filled circles and green open circles represent individual calculations for MACE_deltaL and GFN2-xTB, respectively, with black and grey horizontal line segments indicating their corresponding mean values). **e** Bar chart illustrating the trend between the nearest-neighbor Euclidean distance and the MAE of predicted reaction energy barriers for DORTS−1K (the grey dashed line at a distance of 0.7 marks the defined cutoff for the applicability domain of MACE_deltaL). **f** Violin plot depicting the distribution of transition state geometric RMSDs and absolute errors in predicted reaction energy barriers for the 1073 reactions in Transition1x by MACE_deltaL (light blue and dark blue areas represent geometric RMSD and energy barrier absolute error, respectively; in all violin plots (**a**, **b**, **f**), horizontal black lines indicate the mean values). Source data are provided as a Source Data file.

Next, a comprehensive evaluation of four methods (GFN2-xTB, PaiNN, MACE and MACE_deltaL) was conducted through a systematic test using DORTS-1K (1000 reactions not included in DORTS-9K, corresponding to the green dots in Fig. 3(b)). Following this evaluation, MACE_deltaL successfully optimized 880 transition states in DORTS-1K. The same initial guesses corresponding to these 880 transition states were subsequently re-optimized using MACE, PaiNN, and GFN2-xTB, with DFT-derived transition state geometries and reaction energy barriers serving as the benchmark. MACE_deltaL exhibited superior geometric precision compared to the pure MACE model, with a mean geometric Root Mean Square Deviation (RMSD) of 0.12 Å versus 0.19 Å, respectively (Fig. 5(a)). While GFN2-xTB ranked third with 0.31 Å mean geometric RMSD, its inherent accuracy limitations make it difficult to maintain DFT-level precision in transition state energy and atomic force calculations. Notably, PaiNN model exhibited the worst performance (0.50 Å mean geometric RMSD) among four methods, as the inability of its message-passing mechanism to characterize high-order tensor features frequently leads to convergence into incorrect saddle points for complex transition states. Most importantly, MACE_deltaL achieved improvements in reaction energy barrier prediction (Fig. 5(b)), with the MAE of reaction energy barriers reaching 0.60 kcal/mol, which is below the chemical accuracy threshold (1 kcal/mol). This represents a step-change in accuracy compared to the pure MACE model (0.93 kcal/mol) and PaiNN model (5.28 kcal/mol), marking a fitting accuracy better than ±1 kcal/mol for transition state energies. Based on the above results, the best-performing MACE_deltaL model was selected as DeePEST-OS for subsequent research and analysis.

To investigate how MACE_deltaL achieves its performance gains over pure MACE, its prediction errors for transition state geometric RMSDs and reaction energy barrier absolute errors on DORTS-1K were systematically analyzed and stratified by both reaction class and atom count. Specifically, for reaction class, incorporating Δ-learning yielded consistent gains across all four reaction classes (substitution, addition, ring-opening/closing, and molecular rearrangement), indicating a broad rather than niche benefit (Supplementary Fig. 2). For atom count, Fig. 5(c) presents the comparison of MACE and MACE_deltaL prediction errors (mean geometric RMSD and the MAE of reaction energy barriers) on DORTS-1K with respect to the number of atoms. After incorporating Δ-learning, both the mean geometric RMSD and the MAE of reaction energy barriers exhibited a general decrease, with the magnitude of this error reduction increasing as the number of atoms grew. This result likely stems from the tendency of larger molecular systems, with increasing atomic count, to exhibit more long-range interactions. Incorporating the prior physical knowledge of GFN2-xTB effectively compensates for the inherent limitations of pure MLP models (e.g., MACE) in capturing such long-range interactions.

To quantify the transferability of MACE_deltaL and mitigate the risks of unreliable extrapolation, an applicability domain metric was established based on the Euclidean distance and a 512-dimensional embedding space, which was derived from the embedding layer of MACE_deltaL during inference. Utilizing this metric allows potentially unreliable predictions to be filtered out and unreasonable extrapolation to be prevented. Specifically, the Euclidean distance to the nearest neighbor in the training set (DORTS-9K) was calculated for each sample in the DORTS-1K set using the 512-dimensional embeddings. A smaller Euclidean distance to the nearest neighbor indicates a higher similarity between the test sample and the training set. The relationship between the nearest-neighbor Euclidean distance and the MAE of reaction energy barriers is presented in Fig. 5(e), revealing a clear positive correlation where the MAE rises with increasing nearest-neighbor Euclidean distance. Notably, when the nearest-neighbor Euclidean distance exceeds 0.7 Å, the MAE of predicted reaction energy barriers surpasses the chemical accuracy threshold of 1 kcal/mol. Consequently, a value of 0.7 was defined as the cutoff for the applicability domain. Reactions with nearest-neighbor Euclidean distances within this threshold are considered to fall within the reliable prediction scope of the MACE_deltaL model.

To validate the extrapolation ability of the MACE_deltaL model, it is essential to test its performance on Out-Of-Distribution (OOD) reactions. As illustrated in the chemical space in Fig. 3(c), the reactions in the Transition1x (red dots) and DORTS-9K training set (blue dots) exhibit limited overlap and occupy distinct regions, indicating reaction dissimilarity. This dissimilarity was further quantified using the applicability domain analysis, revealing that 1,038 reactions (~97%) in the Transition1x set exhibited a nearest-neighbor Euclidean distance greater than 0.7 relative to the training data in DORTS-9K. This confirms that Transition1x serves as a representative OOD dataset for testing MACE_deltaL. In this rigorous OOD test, a mean RMSD of 0.11 Å for transition state geometries and an MAE of 3.80 kcal/mol for reaction energy barriers were achieved by the MACE_deltaL model. While these metrics indicate a modest decline in accuracy relative to the performance on the DORTS-1K test set—attributable to the notable structural differences between the Transition1x and DORTS-9K datasets that pose challenges for extrapolation—the predictive accuracy of our MACE_deltaL model remains highly competitive with that of the OA-ReactDiff model (a mean geometric RMSD of 0.129 Å and an MAE of 4.4 kcal/mol for reaction energy barriers)^54^, which was specifically trained on the Transition1x dataset. These results demonstrate the robust extrapolation ability of the MACE_deltaL model.

### Cross-dataset evaluation of DeePEST-OS

Notably, significant advancements have recently been achieved in end-to-end ML approaches for the generation of organic reaction transition states. The React-OT model^16^, developed by Duan et al. using optimal transport theory and the Transition1x database, is an approach for directly generating high-precision transition states from reactant/product conformations. To enable direct comparison between React-OT and DeePEST-OS, the DORTS data preparation pipeline was applied to the Transition1x database using identical data splits: 9000 reactions (80% train, 10% validation, 10% test) and 1,073 reactions in an external test set. For data preparation, a hybrid strategy was implemented: GFN2-xTB-based transition state optimization and IRC calculations yielded benchmark data for 7435 reactions, followed by NMS sampling at critical IRC conformations, which generated 695,138 molecular structures. All structures were then computed at the DFT level used in Transition1x (ωB97x/6-31 G(d)). The MLP model was trained by unifying GFN2-xTB physical priors with the MACE architecture through a Δ-learning strategy, which demonstrated exceptional energy and atomic force prediction performance (MAE: 0.859 kcal/mol (energy), 0.841 kcal/mol/Å (force); RMSE: 1.245 kcal/mol (energy), 1.479 kcal/mol/Å (force); see Supplementary Fig. 3). In the following, this MLP model was named DeePEST-OS-T1x to distinguish it from the previously mentioned the DeePEST-OS model trained on DORTS-9K. A comparative analysis of transition state geometric RMSDs, reaction energy barriers absolute errors, and computational costs is performed on 1073 external test reactions using DeePEST-OS-T1x and React-OT models. DFT-optimized geometries and energies, calculated using Gaussian 16^55^, served as the reference standards to ensure consistency in structural and energetic evaluations. Among the 995 reactions for which transition states were successfully optimized using DeePEST-OS-T1x, React-OT, and DFT (Table 2), DeePEST-OS-T1x demonstrated superior accuracy over React-OT, with a lower mean geometric RMSD (0.050 Å vs. 0.077 Å) and a smaller MAE of reaction energy barriers (0.692 kcal/mol vs. 1.038 kcal/mol) (see the violin plots in Supplementary Fig. 4). Notably, DeePEST-OS-T1x was also more efficient, requiring an average of only 1.4 s per reaction for transition state optimization on an NVIDIA GeForce RTX 4060 Laptop GPU, whereas React-OT required 1.8 s per reaction. In addition, a distinct advantage of DeePEST-OS-T1x is its ability to simultaneously output a near-DFT-level predicted energy during transition state optimization. In contrast, React-OT focuses solely on geometry and does not provide energy outputs during transition state generation.

**Table 2:** Comparison of transition state geometric RMSDs, reaction energy barrier absolute errors, and computational costs between DeePEST-OS-T1x and React-OT models on the Transition1x external test set

Model	Geometric RMSD (Å)		Energy barrier absolute error (kcal/mol)		Computational cost (s)
	Mean	Median	Mean	Median	Transition state optimization
React-OT	0.077	0.030	1.038	0.565	1.8
DeePEST-OS-T1x	0.050	0.026	0.692	0.334	1.4

Furthermore, to investigate the relationship between energy absolute errors and geometric RMSDs of transition states, DeePEST-OS-T1x was compared with OA-ReactDiff—an end-to-end generative model for transition state geometry prediction developed by Duan et al. and trained on the Transition1x database^54^. To enable a consistent comparison, a similar correlation analysis was performed using the same test split and a log-log linear fit (Supplementary Fig. 5). The regression equation of DeePEST-OS-T1x is $\log \left(y\right)=0.45\log \left(x\right)+0.18$ with a Pearson correlation coefficient (*r*) value of 0.35, whereas the regression equation of OA-ReactDiff is $\log \left(y\right)=0.63\log \left(x\right)+0.99$ with *r* = 0.56. Both low *r* values indicate only a moderate-to-weak linear relationship between transition state geometric RMSDs and energy absolute errors. Notably, the smaller slope and intercept of DeePEST-OS-T1x indicate that, for a given transition state RMSD, its fitted relationship implies a lower energy absolute error compared to OA-ReactDiff. A plausible explanation is that DeePEST-OS-T1x is trained directly on energies and forces (mitigating energy-related discrepancies), whereas OA-ReactDiff is primarily optimized for minimizing transition state geometric RMSDs. This observation aligns with the findings by King et al. ^56^, who demonstrated that although models achieve high structural precision with low geometric RMSD, sole optimization for this metric fails to ensure accurate energy predictions. This limitation arises from the sensitivity of energy to minor geometric deviations, highlighting the critical need to incorporate energy constraints during model training.

### Transition state conformational isomer screening driven by DeePEST-OS

In the study of organic reaction mechanisms, conformational isomers play a critical role in determining reaction energy barriers, as subtle differences in transition state conformations can lead to qualitative changes in energy barriers^57^. Precisely determining the most stable transition state conformational isomer becomes crucial for understanding reaction selectivity and rate control. Traditional conformational search methods face dual challenges when applied to transition state systems: maintaining geometric features at reactive sites while effectively sampling sidechain conformational space. The GENConf-TS algorithm^57^, developed in our earlier work, currently addresses this by selectively freezing critical coordinates at transition state reactive centers while enabling multi-dimensional rotational scanning of sidechains connected to the reaction core. The method first optimizes generated conformations using the GFN2-xTB approach, then ranks thermodynamic stability based on standard enthalpy calculations and Boltzmann-weighted statistics. Final validation employs rigorous DFT optimizations to confirm the most stable conformer. Notably, while GENConf-TS enables automated searches of transition state conformational isomers, the high computational cost of DFT severely limits efficiency, particularly in multi-substituted or flexible systems. The integration of GENConf-TS with DeePEST-OS achieves high-throughput screening of transition state conformers, establishing a theoretical paradigm for studying transition state conformations in complex organic reaction systems.

**Fig. 6:**
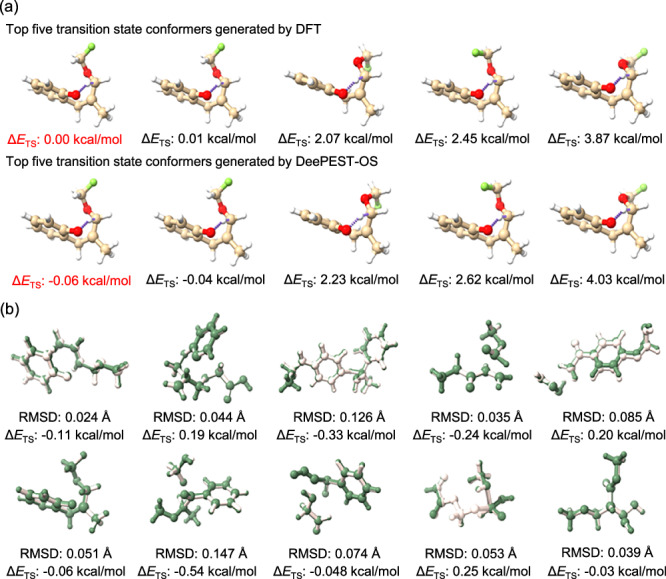
Screening of transition state conformational isomers based on GENConf-TS. **a** Energy predictions of the five most stable transition state conformational isomers using DFT and DeePEST-OS methods (Δ*E*_TS_ is used to represent the energy difference relative to the DFT-calculated most stable conformation, with the lowest energy conformation highlighted in red). **b** Geometric structure comparisons (RMSD) and energy differences (Δ*E*_TS_) between DFT-optimized (white structures) and DeePEST-OS-optimized (green structures) transition state conformations for ten complex reactions. Source data are provided as a Source Data file.

This study selected ten complex reactions from DORTS-1K for transition state conformational isomer screening. Taking one representative reaction involving C, H, O, F atoms as an example (Fig. 6(a)), the top five transition state conformational isomers predicted by DeePEST-OS were compared against those obtained through DFT optimization. Both methods demonstrated consistency in conformational rankings, with a minor energy error of 0.06 kcal/mol for the most stable conformation. Figure 6(b) displays transition state geometric comparisons and energy differences between DFT-optimized and DeePEST-OS-optimized structures across ten complex reactions involving C, H, O, N, S, and F atoms. DeePEST-OS consistently identified the same most stable conformations as DFT in all tested systems. This work demonstrates that MLP achieves DFT-level accuracy in transition state conformational screening while overcoming traditional limitations in computational efficiency and conformational sampling capability, thereby providing a robust tool for stereoselectivity studies in complex organic reactions.

### Kinetic prediction of multi-step organic synthesis driven by DeePEST-OS

Kinetic investigations of multi-step organic synthesis face core challenges, such as the accurate identification of transition states, the analysis of energy changes in intermediates, and the coupling of reaction pathways. While traditional QC approaches like DFT provide accurate PES information, their computational cost increases exponentially with reaction steps, severely limiting exploration of complex reaction networks^58^. The DeePEST-OS model addresses these limitations through ML-enabled rapid transition state searches (seconds-level optimization per transition state) coupled with DFT-comparable geometric/energy accuracy. This innovative methodology not only efficiently constructs complete PESs for multi-step reactions but also precisely captures how transition state conformational isomers influence reaction selectivity, providing an efficient and precise analytical methodology for kinetic studies of complex organic reaction networks.

**Fig. 7:**
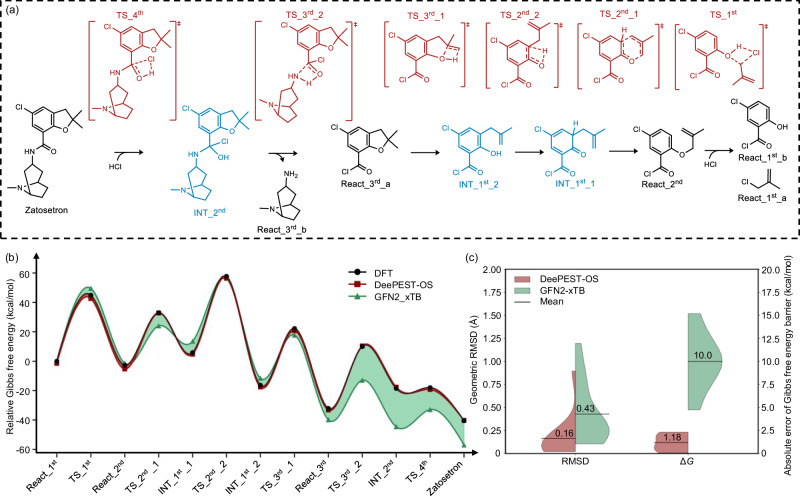
Kinetic prediction of multi-step organic synthesis for Zatosetron compound using DeePEST-OS. **a** The optimal reaction pathway for Zatosetron synthesis co-constructed by retrosynthesis software RetroSynX and initial guess of transition state generator GENiniTS-RS (black structures represent the synthesis pathway designed by RetroSynX, red structures indicate transition state conformations generated by GENiniTS-RS, and blue structures correspond to intermediate conformations along the IRC pathways). **b** Multi-step reaction potential energy profiles of Zatosetron synthesis calculated by DFT, DeePEST-OS, and GFN2-xTB (the solid black line with circles, solid red line with squares, and solid green line with triangles denote DFT, DeePEST-OS, and GFN2-xTB, respectively; the dark red and green shaded areas represent the deviation of DeePEST-OS and GFN2-xTB from DFT, respectively). **c** Violin plots comparing RMSD of transition state/intermediate geometries and absolute error of Gibbs free energy barriers between DeePEST-OS and GFN2-xTB, with DFT calculations as reference (red and green areas correspond to DeePEST-OS and GFN2-xTB, respectively; horizontal solid black lines indicate the mean values). Source data are provided as a Source Data file.

This study evaluates the comprehensive performance of DeePEST-OS in complex organic reaction explorations through a full-process validation of the Zatosetron multi-step synthesis pathway (Fig. 7). The workflow began with reaction path inference using retrosynthesis software RetroSynX^59^ (Fig. 7(a)), generating SMILES strings for multi-step reactants. Subsequently, initial guesses of transition states were constructed via GENiniTS-RS, and the resulting 3D transition state structures were fed into GENConf-TS to generate conformational isomers. DeePEST-OS was then employed to identify the most stable intermediate and transition state conformers, along with calculating Gibbs free energy barriers for elementary reactions.

Comparative analysis in Fig. 7(b) demonstrates that the DeePEST-OS predicted reaction barriers exhibited exceptional agreement with DFT benchmarks, while GFN2-xTB predictions showed error escalation with increasing system complexity (DeePEST-OS maintains stable accuracy). Geometry and energy barrier error analyses (Fig. 7(c)) reveal that DeePEST-OS achieved lower mean geometric RMSD (0.16 Å for intermediates/transition states) and MAE of Gibbs free energy barriers (1.18 kcal/mol) compared to GFN2-xTB (0.43 Å and 10.00 kcal/mol, respectively), confirming the precision of DeePEST-OS in multi-step barrier prediction. Notably, the TS_3^rd^_2 and TS_4^th^ steps in Zatosetron synthesis involve 51-atom systems that exceed the maximum molecular scale in the DORTS database and include unseen structures such as React_3^rd^_b. These results indicate that DeePEST-OS, through autonomous resolution of interatomic interaction patterns rather than reliance on training-data memorization, effectively handles molecular systems beyond the scope of training set. DeePEST-OS shows potential for applications in complex organic synthesis, particularly in scenarios requiring extrapolation beyond existing computational chemistry datasets.

### Experimental validation of endo and exo diastereoselectivity in Diels-Alder reactions

**Fig. 8:**
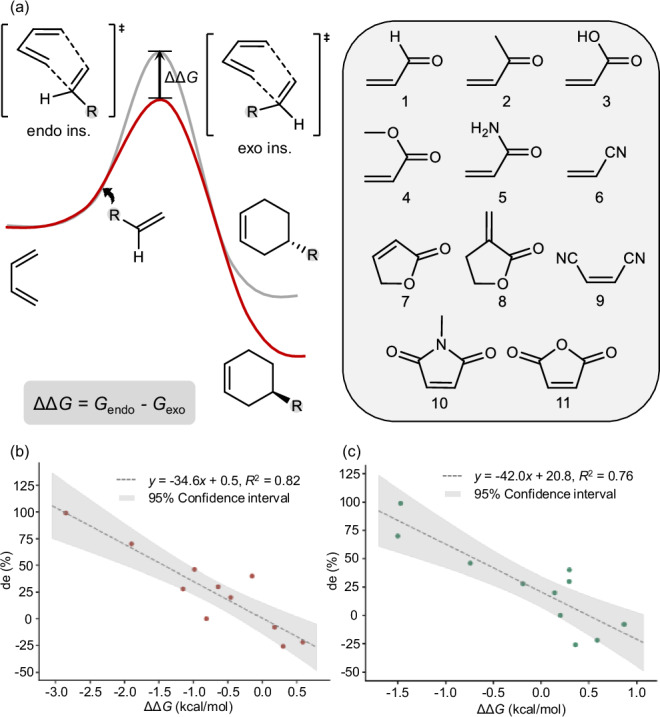
Linear fitting between ΔΔ*G* and de values for endo and exo insertions of different reactants in Diels-Alder reactions. **a** ΔΔ*G* definition and reactant structures. **b** Linear fitting between DFT-calculated ΔΔ*G* and experimental de values, with the shaded area indicating the 95% confidence interval. **c** Linear fitting between DeePEST-OS-calculated ΔΔ*G* and experimental de values, with the shaded area indicating the 95% confidence interval. Source data are provided as a Source Data file.

To evaluate the error between DeePEST-OS predictions and experimental data, the endo/exo diastereoselectivity of the Diels-Alder reaction was tested as a case study. Experimental diastereomeric excess (de) values were sourced from the literature^60^, and theoretically, de is fundamentally correlated with the Gibbs free energy barrier difference (ΔΔ*G*) between the endo and exo insertions. To predict these ΔΔ*G* values, DFT calculations were employed at the ωB97M-V/def2-TZVP level, as this DFT level maintains consistency with the DeePEST-OS training database (DORTS-9K) and exhibits excellence in predicting thermodynamic properties^52^. Furthermore, conformational isomerism was explicitly considered, since it has been shown to improve the quantitative agreement between theoretical calculations and experimental observations^61,62^. After evaluating the ΔΔ*G* values for 11 distinct reactants, a least-squares linear regression was performed to correlate the DFT-calculated values with the experimental de values, as depicted in Fig. 8(b). The resulting coefficient of determination (*R*)^2^ was 0.82, demonstrating that this benchmark DFT methodology has the capacity to provide reliable predictions of experimental de trends for the endo/exo diastereoselectivity of the Diels-Alder reaction.

To further evaluate DeePEST-OS, Fig. 8(c) displays the correlation between DeePEST-OS-predicted ΔΔ*G* values and experimental de values. The resulting *R*^2^ of 0.76 demonstrated a reasonably strong quantitative correlation. Although this *R*^2^ was slightly lower than that achieved by the DFT method, DeePEST-OS preserves the DFT-inherent predictive capacity for de values to facilitate experimental exploration.

## Discussion

The persistent trade-off between quantum-level accuracy and computational efficiency has limited comprehensive exploration of organic reaction kinetics. To overcome this long-standing challenge, this work developed DeePEST-OS based on the MACE architecture and the Δ-learning strategy, achieving DFT-comparable accuracy (energy MAE: 0.266 kcal/mol, force MAE: 0.380 kcal/mol/Å) while accelerating transition state optimization by four orders of magnitude. Addressing data scarcity, a hybrid data preparation strategy combined semi-empirical efficiency for exhaustive conformational sampling (<0.01% cost of full DFT workflows) with DFT precision for final energy/force annotations, ensuring broad chemical space coverage coupled with quantum-level reliability. This strategy enabled the creation of the DORTS database, a landmark resource encompassing 74,837 diverse reactions across ten essential main-group elements (C, H, O, N, S, P, F, Cl, Br, I). DORTS significantly exceeds established benchmarks like Transition1x in element types, reaction types, number of reactions, and calculation level. Demonstrating exceptional generalizability, DeePEST-OS delivered high accuracy on 1000 reactions excluded from training, achieving a mean RMSD of 0.12 Å for transition state geometries and an MAE of 0.60 kcal/mol for reaction barriers compared to DFT. Critically, in rigorous cross-dataset evaluation, DeePEST-OS-T1x (based on Transition1x) demonstrates superior performance, surpassing React-OT in both transition state geometric precision (mean RMSD: 0.050 Å vs. 0.077 Å) and reaction energy barrier accuracy (MAE: 0.692 kcal/mol vs. 1.038 kcal/mol), alongside improved computational efficiency (1.4 s vs. 1.8 s per transition state). Transcending conventional MLPs constrained to C, H, N, O systems, DeePEST-OS leverages coverage of ten elements to unlock mechanism-guided retrosynthesis of complex drug candidates featuring essential heteroatoms (e.g., halogens, sulfur, phosphorus) such as Zatosetron. This broad applicability delivers DFT-level accuracy (MAE of Gibbs free energy barriers: 1.18 kcal/mol) even for challenging, out-of-training-set systems (>50 atoms). In experimental validations using Diels-Alder reactions, a reasonably strong quantitative agreement with measured de values was achieved (*R*^2^ = 0.76), confirming the ability of DeePEST to facilitate experimental exploration. By addressing the accuracy-efficiency dilemma while transcending elemental limitations, DeePEST-OS provides a scalable platform for systematic navigation of complex reaction networks, enabling rapid mechanistic elucidation, rational synthetic pathway optimization, and stereoselectivity prediction across diverse organic systems with extensive elemental coverage.

This study demonstrates that DeePEST-OS exhibits unique advantages and broad application prospects in organic reaction mechanism research. However, it should be noted that despite the hybrid data preparation strategy proposed in this study, the development process still requires substantial high-precision QC computations, particularly in energy/force matrix calculations for conformations of reaction systems. Therefore, the key in future MLP development lies in establishing adaptive sampling mechanisms integrated with active learning strategies, potentially achieving orders-of-magnitude reductions in data acquisition costs through intelligent pre-screening algorithms for conformational energy/force annotations.

As a semi-empirical method, GFN2-xTB exhibits a computational cost scaling approximately as O(*N*)^3^ with respect to the number of atoms^63^, leading to high computational demands for MACE_deltaL when applied to large molecular systems. To evaluate practical performance, the inference times of GFN2-xTB and MACE_deltaL for transition state geometry optimization were assessed on DORTS-1K. Figure 5(d) compares the inference times across reactions with varying atom counts. Both methods demonstrated increased computation times with growing system size, with MACE_deltaL being slower than GFN2-xTB due to its coupling of GFN2-xTB and MACE. Although GFN2-xTB exhibits an approximate O(*N*)^3^ scaling that limits its efficiency for large systems, the reaction systems studied in this work were moderate in size (typically 6-50 atoms), thus its computational efficiency remained acceptable. The average inference time of MACE_deltaL (0.084 core·s·step^⁻1^) was approximately 2.53-fold higher than that of GFN2-xTB (0.033 core·s·step^⁻1^), yet remained below 0.1 core·s·step^⁻1^. Consequently, MACE_deltaL maintains sufficient efficiency to satisfy the requirements of high-throughput transition state geometry optimizations for the currently studied system sizes. However, future extension to substantially larger systems will face significant computational-efficiency challenges.

Another point worthy of note is that MACE_deltaL employs Δ-learning coupled with the GFN2-xTB semi-empirical method for both training and inference. Therefore, its applicability is inherently constrained by the limitations of GFN2-xTB. Although GFN2-xTB exhibits relatively excellent performance in organic systems (the mean RMSD of transition state geometries optimized by GFN2-xTB in Fig. 5(a) is 0.31 Å), it may have accuracy limitations in metal-containing reaction systems. For future training of Δ-learning models for metal-containing systems, attempts can be made to couple the g-xTB method^64^, which performs better in metal systems, or pre-trained MLP models for metal-containing systems^65^.

Furthermore, DeePEST-OS requires an initial guess of the transition state for transition state optimization. However, the automatic generation of initial guesses of transition states in this work was constrained by reliance on reaction templates. Future extensions could broaden the applicability of DeePEST-OS by integrating template-free algorithms for generating initial guesses of transition states, such as React-OT^16^. These technological advancements will collectively propel computational organic chemistry toward a quantum accuracy-high throughput screening synergistic paradigm, ultimately establishing an experiment-oriented intelligent prediction framework for organic synthesis design.

## Methods

Computer-Aided Molecular Design (CAMD) employs the BARON solver^47^ to solve the MILP model, which automatically assembles molecular scaffolds with selected groups to generate SMILES-based organic reactants based on 255 reaction templates. The structure constraints of the formulated MILP model, as presented in Eqs. (1–5), ensure the chemical feasibility and structural validity of the generated reactants through systematic enforcement of molecular connectivity rules and valence requirements.

1 $$1\le {\sum}_{i{{{\rm{\epsilon }}}}{G}_{1}}{n}_{i}\le 1$$

2 $$2\le {\sum}_{i{{{\rm{\epsilon }}}}{G}_{2}}{n}_{i}\le 6$$

3 $${n}_{i}\le 3,i\in {G}_{2}$$

4 $$1\le {\sum}_{i{{{\rm{\epsilon }}}}{G}_{3}}{n}_{i}\le 4$$

5 $$0\le {\sum}_{i{{{\rm{\epsilon }}}}({G}_{2}-{G}_{3}-{G}_{1})}{n}_{i}\le 2$$

where ${n}_{i}$ is the number of fragments (scaffolds and groups) $i$ involved in a reactant candidate, ${G}_{1}$ is the scaffold set, ${G}_{2}$ is the fragment set, and ${G}_{3}$ is a subset of ${G}_{2}$ (${G}_{3}=\left\{{{{{\rm{CH}}}}}_{3},\,{{{{\rm{CH}}}}}_{2},\,{{{\rm{CH}}}},{{{\rm{and\; C}}}}\right\}$).

The GENiniTS-RS algorithm automatically generates initial guesses of transition states by utilizing 255 SMARTS-encoded organic reaction templates containing three-dimensional Cartesian coordinates of reactive sites, which facilitates rapid conversion of SMILES-represented reactants into initial guesses of transition states. As illustrated in Fig. 2(a), these reaction templates incorporate predefined spatial arrangements of reactive centers. The workflow primarily involves two key operations: (1) the automated generation of approximate initial 3D geometries for reactants through molecular mechanics methods based on their SMILES strings, and (2) the systematic replacement of reactive site coordinates in reactants with template-derived Cartesian coordinates. Subsequent geometry optimization includes translational adjustment and stochastic rotation of side chains, where the distance geometry method is employed to prevent atomic clashes while maintaining chemically plausible molecular conformations. Final refinement combines molecular mechanics optimization with semi-empirical QC calculations to yield physically reasonable initial guesses of transition states. The detailed algorithm is comprehensively described by Liu et al. ^48^.

The GFN2-xTB method, grounded in the Geometry, Frequency, Noncovalent, extended Tight-Binding (GFN-xTB) theory, enhances the treatment of electron correlation effects and non-bonded interactions (e.g., van der Waals forces, hydrogen bonding), enabling efficient geometry pre-optimization for organic molecules. Detailed theoretical formulations of GFN2-xTB are documented in the work by Grimme et al. ^7^. In this study, the GFN2-xTB approach was implemented via a Gaussian-xTB interface developed by Lu Tian^49^, with Gaussian16 being employed to perform geometry optimization of initial guesses of transition states and IRC calculations.

The conventional Normal Mode Sampling (NMS) method employs random displacement sampling along fixed normal mode directions to generate candidate structures through configurational distortion. This approach replaces computationally expensive full-scale ab initio molecular dynamics simulations with combined Hessian matrix calculations and single-point electronic structure computations, thereby substantially reducing computational costs. The thermal probability distribution function ${\rho }_{{{{\rm{c}}}}}$ governing molecular distributions on the PES at temperature $T$, along with the reference thermal geometry ${{{\bf{R}}}}$, is defined by Eqs. (6–8). In this formulation, ${\omega }_{i}$ and ${\Omega }_{i}$ represent the natural frequency and normal coordinate corresponding to the *i*-th normalized vibrational mode vector, respectively, where $\beta$ denotes the inverse temperature ($\beta=\frac{1}{{{{{\rm{k}}}}}_{{{{\rm{B}}}}}T}$, with ${{{{\rm{k}}}}}_{{{{\rm{B}}}}}$ being the Boltzmann constant), $N$ specifies the total internal degrees of freedom (typically $3N-6$ for an $N$-atom system), and ${v}_{i}$ symbolizes the normalized vibrational vector of the *i*-th mode.

6 $$\beta=\frac{1}{{{{{\rm{k}}}}}_{{{{\rm{B}}}}}T}$$

7 $${\rho }_{{{{\rm{c}}}}}\left({\varOmega }_{1},{\varOmega }_{2},\ldots,{\varOmega }_{N}\right)\propto \exp \left(-\beta {\sum}_{i=1}^{N}\frac{{\omega }_{i}^{2}{\varOmega }_{i}^{2}}{2}\right)$$

8 $${{{\bf{R}}}}={{{{\bf{R}}}}}_{0}+{\sum}_{i=1}^{N}\sqrt{\frac{\beta }{{\omega }_{i}^{2}}} \, {\varOmega \, }_{i}\cdot {{{{\bf{v}}}}}_{i}$$

The quantum thermal NMS methodology extends thermal density distributions by quantum broadening harmonic oscillator wavefunctions, as formulated in Eqs. (9–11). The modified inverse temperature ${\beta }^{*}$ incorporates the reduced Planck constant ${{\hslash }}$ through the term $\tan h\left(\frac{\beta {{\hslash }}\omega }{2}\right)$, where the hyperbolic tangent function asymptotically approaches unity with increasing argument while remaining strictly below linear growth, thereby inherently satisfying ${\beta }^{*}$ < $\beta$. This mathematical relationship indicates that quantum harmonic oscillators persistently operate at effective temperatures exceeding their classical reference temperature $T$. Consequently, both the probability density distribution ${\rho }_{{{{\rm{c}}}}}$ and reference thermal geometry ${{{\bf{R}}}}$ exhibit systematically broader dispersion profiles compared to classical counterparts. For comprehensive theoretical derivations and implementation specifics of quantum thermal NMS, readers are directed to the foundational work by Marsalek et al. ^50^.

9 $${\beta }^{*}=\frac{2}{\hslash \omega }\tanh \left(\frac{\beta \hslash \omega }{2}\right)$$

10 $${\rho }_{c}\left({\varOmega }_{1},{\varOmega }_{2},\ldots,{\varOmega }_{N}\right)\propto \exp \left(-{\beta }^{*}{\sum}_{i=1}^{N}\frac{{\omega }_{i}^{2}{\varOmega }_{i}^{2}}{2}\right)$$

11 $${{{\mathbf{R}}}}={{{{\mathbf{R}}}}}_{0}+{\sum}_{i=1}^{N}\sqrt{\frac{{\beta }^{*}}{{\omega }_{i}^{2}}}{\varOmega }_{i}\cdot {{{{\mathbf{v}}}}}_{i}$$

The ωB97M-V functional has been rigorously validated through extensive benchmarking studies as one of the most accurate density functionals across diverse quantum chemical applications^51^. The def2-TZVP basis set^66^, employing triple-ζ valence quality with polarization functions, achieves optimal balance between computational efficiency and accuracy for main-group element systems. All DFT calculations were performed using the ORCA 6.0.0 software package^67^, incorporating dual integral acceleration through resolution-of-identity for Coulomb integrals^68^ and chain-of-spheres exchange^69^ algorithms.

MACE implements a message passing neural network architecture where molecular systems are represented as graphs with atoms as vertices and interatomic connections as edges within a specified radial cutoff. Each atomic node state at layer $t$, denoted by the tuple ${\sigma }_{i}^{t}$ in Eq. (12), integrates spatial coordinates ${{{{\bf{r}}}}}_{i}$, elemental identifiers ${z}_{i}$, and evolving feature vectors ${{{{\bf{h}}}}}_{i}^{t}$. Feature refinement occurs through successive message-passing operations, where spherical harmonic expansions encode angular dependencies between neighboring atoms, while radial basis functions parameterize pairwise distances collectively ensuring E(3)-equivariant representations.

12 $${\sigma }_{i}^{t}=\left({{{{\bf{r}}}}}_{i},{z}_{i},{{{{\bf{h}}}}}_{i}^{t}\right)$$

Notably, MACE employs a hierarchical message construction scheme (Eq. 13) through the atomic cluster expansion framework^70^, enabling efficient generation of high-body-order interactions (up to order $\nu$) via learnable functions $u$.

13 $${m}_{i}^{\left(t\right)}=	 {\sum}_{j}{u}_{1}\left({\sigma }_{i}^{t},{\sigma }_{j}^{t}\right)+{\sum}_{{j}_{1},{j}_{2}}{u}_{2}\left({\sigma }_{i}^{t},{\sigma }_{{j}_{1}}^{t},{\sigma }_{{j}_{2}}^{t}\right)+\ldots \\ 	+{\sum}_{{j}_{1},\ldots {,j}_{\nu }}{u}_{\nu }\left({\sigma }_{i}^{t},{\sigma }_{{j}_{1}}^{t},\ldots,{\sigma }_{{j}_{\nu }}^{t}\right)$$

This design circumvents the computational limitations of conventional two-body message passing neural networks by systematically aggregating multi-atom correlations through tensor product operations on spherical harmonics, thereby enhancing both expressivity and convergence rate. Following multiple message propagation layers, atomic environment descriptors progressively incorporate medium-range chemical effects, with final site energy predictions derived from node states through invariant feature pooling. The architecture demonstrates superior performance in capturing complex quantum mechanical relationships compared to traditional neural network potentials. Note that the NVIDIA cuEquivariance library, a CUDA-accelerated library tailored for equivariant neural networks, was used to accelerate MACE training and inference in this work. The hyperparameters employed for MACE training are detailed in Supplementary Table 3 of the Supporting Information.

The Δ-learning methodology for molecular property prediction involves statistically modeling the difference between a baseline approximation and a target high-accuracy value. Specifically, for optimization objectives such as molecular energies or atomic forces, preliminary calculations are performed using computationally inexpensive baseline theoretical methods (e.g., GFN2-xTB), while reference values are obtained through rigorous QC calculations employing higher-accuracy, resource-intensive target methods (e.g., DFT).

14 $${E}_{{{{\rm{target}}}}}={E}_{{{{\rm{DFT}}}}}-{E}_{{{{\rm{GFN}}}}2-{{{\rm{xTB}}}}}$$

15 $${F}_{{{{\rm{target}}}}}={F}_{{{{\rm{DFT}}}}}-{F}_{{{{\rm{GFN}}}}2-{{{\rm{xTB}}}}}$$

The MLP model is trained to predict the systematic discrepancies (Δ-values) between these two computational tiers through supervised learning. This multi-fidelity approach enables efficient property prediction by combining rapid baseline calculations with ML corrections that emulate the accuracy of advanced electronic structure methods while avoiding their prohibitive computational costs.

The Sella algorithm, a widely adopted computational method for automated saddle point localization in redundant internal coordinate systems, enhances convergence efficiency through iterative Hessian matrix updates derived from differentiation-based trajectory analysis. This methodology strategically integrates historical force derivative data to construct dynamically improved Hessian approximations during optimization cycles, as comprehensively described in Hermes et al. ^44^. The implementation in this work employed stringent convergence thresholds: maximum perpendicular force (${F}_{\max }\,\le \,0.0025\,{{{\rm{eV}}}}/{{{\mathrm{\AA}}}}$) for transition state geometries and ${F}_{\max }\,\le \,0.005\,{{{\rm{eV}}}}/{{{\mathrm{\AA}}}}$ for IRC calculations. To account for stochastic variations in conformation sampling during the search process, duplicate optimization attempts with automatic filtration were instituted—structures that failed to converge across both cycles were eliminated, while successfully optimized transition states retained associated IRC trajectories, final geometries, and computed reaction barriers. This protocol ensures robust identification of transition states while maintaining computational tractability through systematic elimination of non-productive search pathways.

## Supplementary information

Supporting information

Transparent Peer Review file

## Source data

Source Data

## Data Availability

Source data are provided with this paper. The DORTS database generated in this study has been deposited in Zenodo under accession code 10.5281/zenodo.17141108 [10.5281/zenodo.17141108]^71^. The Transition1x dataset used in this study is available in GitLab [https://gitlab.com/matschreiner/Transition1x]^72^. Source data are provided with this paper.

## Code availability

The DeePEST-OS model and its inference codes have been deposited in a private repository via GitHub [https://github.com/kaipai-ren/DeePEST-OS/tree/main] and a public repository via Zenodo under accession code 10.5281/zenodo.19305799 [10.5281/zenodo.19305799]^73^. The React-OT model used in this study is available in Zenodo under accession code 10.5281/zenodo.14836384 [10.5281/zenodo.14836384]^74^.
