# Supplementary material for: Reactive machine learning potential for accelerating transition state search in organic synthesis
Source: Nat Commun. 2026 May 8;17:6253. doi: 10.1038/s41467-026-72945-0 (PMC13376211; doi:10.1038/s41467-026-72945-0)
Supplement: Supplementary file 1 — Supporting information [file 41467_2026_72945_MOESM1_ESM.pdf]

Supplementary Information

Reactive Machine Learning Potential for Accelerating Transition State Search in Organic Synthesis

Kaipai Ren[a,#], Kun Tang[a,#], Yujing Zhao[a,b], Lei Zhang[a,c], Jian Du[a,c], Qingwei Meng[a,c], Qilei Liu[a,c*]

[a]State Key Laboratory of Fine Chemicals, Frontiers Science Center for Smart Materials Oriented Chemical Engineering, Department of Pharmaceutical Sciences, Institute of Chemical Process Systems Engineering, School of Chemical Engineering, Dalian University of Technology, Dalian 116024, China

[b]MOE Key Laboratory of Bio-Intelligent Manufacturing, School of Bioengineering, Dalian University of Technology, Dalian 116024, China

[c]Ningbo Institute of Dalian University of Technology, Ningbo 315016, China

[#]These authors contributed equally to this work

**Corresponding author: Qilei Liu (liuqilei@dlut.edu.cn)*

Suppl. Table 1 The complete list of 255 reaction templates (these templates were extracted from March's Advanced Organic Chemistry and encoded as SMARTS (SMiles ARbitrary Target Specification) to represent diverse fundamental organic reactions).

No.	SMARTS
1	[C:1](=[O:2])[O:3][C;H3:4].[O;!H0:5]>>[C:1](=[O:2])[O:3][H:6].[13C:4][17O:5]
2	[C:1](=[O:2])[Cl:3].[N;!H0:4][c:5]1[c:6][c:7][c:8][c:9][c:10]1>>[C:1](=[O:2])[N:4][c:5]1[c:6][c:7][c:8][c:9][c:10]1.[Cl:3][H:11]
3	[C:1](=[O:2])[Br:3].[N;!H0:4][c:5]1[c:6][c:7][c:8][c:9][c:10]1>>[C:1](=[O:2])[N:4][c:5]1[c:6][c:7][c:8][c:9][c:10]1.[Br:3][H:11]
4	[C:2](=[O:3])[O:4][C:5]=[O:6].[N;!H0:1]>>[C:2](=[O:3])[N:1].[C:5](=[O:6])[O:4][H:7]
5	[C:1](=[O:2])[Cl:3].[C;!$(C=O):5][O;H1:4]>>[C:1](=[O:2])[O:4][C;!$(C=O):5].[Cl:3][H:6]
6	[C:1](=[O:2])[O;!H0:3].[N;!H0:4]>>[C:1](=[O:2])[N:4].[O:3][H:5]
7	[Cl:1][C:2](=[O:3])[Cl:4].[C;!$(C=O):5][O;!H0:6]>>[Cl:1][C:2](=[O:3])[O:6][C:5].[Cl:4][H:7]
8	[Cl:1][C:2](=[O:3])[O:4][C:5].[C:6][O;!H0:7]>>[C:6][O:7][C:2](=[O:3])[O:4][C:5].[Cl:1][H:8]
9	[C:1][C:2](=[O:3])[Cl:4].[C;!$(C=O):5][O:6][C;!$(C=O):7]>>[C:1][C:2](=[O:3])[O:6][13C:5].[C:7][Cl:4]
10	[C:1](=[O:2])[N:3]([C:4])[C:5].[C;!$(C=O):6][O;!H0:7]>>[C:1](=[O:2])[O:7][C:6].[13C:4][N:3]([H:8])[C:5]
11	[C:1](=[O:2])[O;!H0:3].[C:4](=[O:5])[O:6]>>[13C:1](=[O:2])[17O:3][C:4](=[O:5]).[H:7][O:6]
12	[C:1](=[O:2])[O;!H0:3].[C:4](=[O:5])[O:6][C:7]([C:8])=[C:9]>>[C:1](=[O:2])[O:3][C:4](=[O:5]).[H:10][O:6][C:7]([C:8])=[C:9]
13	[C:1](=[O:2])[O:3].[N;!H0:4][c:5]1[c:6][c:7][c:8][c:9][c:10]1>>[13C:1](=[17O:2])[N:4][c:5]1[c:6][c:7

][c:8][c:9][c:10]1.[O:3][H:11]

14 [C:1](=[O:2])[Cl:3].[S;!H0:4]>>[C:1](=[O:2])[S:4].[Cl:3][H:5]

15 [c:10]1[c:9][c:8][c:7][c:6][c:1]1[C:2](=[O:3])[O:4].[N;!H0:5]>>[c:10]1[c:9][c:8][c:7][c:6][c:1]1[C:2](=[O:3])[N:5].[H:11][O:4]

16 [c:10]1[c:9][c:8][c:7][c:6][c:5]1[C:1](=[O:2])[Cl:3].[S;!H0:4]>>[c:10]1[c:9][c:8][c:7][c:6][c:5]1[C:1](=[O:2])[S:4].[Cl:3][H:11]

17 [C:1][C:2](=[O:3])[O:4].[N;!H0:5]>>[C:1][C:2](=[O:3])[N:5].[H:6][O:4]

18 [C:1](=[O:2])[Cl:3].[N;!H0:4][C:5]=[O:6]>>[C:1](=[O:2])[N:4][C:5]=[O:6].[Cl:3][H:7]

19 [C:1](=[O:2])[Br:3].[N;!H0:4]>>[C:1](=[O:2])[N:4].[Br:3][H:5]

20 [N;H3:1].[Cl:2][C:3](=[O:4])[O:5][c:6]1[c:7][c:8][c:9][c:10][c:11]1>>[N;H2:1][C:3](=[O:4])[O:5][c:6]1[c:7][c:8][c:9][c:10][c:11]1.[H:12][Cl:2]

21 [C;!$(C=O);!$(CO):1][C:2](=[O:3])[Cl:4].[F;H1:5]>>[C:1][C:2](=[O:3])[F:5].[Cl:4][H:6]

22 [C:1][C:2]([C:3])=[C;H1:4][C:5].[Cl:6][C:7](=[O:8])[C:9]>>[C:1][C:2]([C:3])=[13C:4]([C:5])[C:7](=[O:8])[C:9].[Cl:6][H:10]

23 [c:12]1[c:11][c:10][c:9][c:8][c:7]1[C:1]([c:13]2[c:14][c:15][c:16][c:17][c:18]2)=[C;H1:2][c:19]3[c:20][c:21][c:22][c:23][c:24]3.[Cl:3][C:4](=[O:5])[C:6]>>[c:12]1[c:11][c:10][c:9][c:8][c:7]1[C:1]([c:13]2[c:14][c:15][c:16][c:17][c:18]2)=[C:2]([c:19]3[c:20][c:21][c:22][c:23][c:24]3)[C:4](=[O:5])[C:6].[Cl:3][H:25]

24 [C;!$(C=O);!$(CO):1][C:2](=[O:3])[Cl:4].[F;H1:5]>>[C:1][C:2](=[O:3])[F:5].[Cl:4][H:6]

25 [C:1][C:2](=[O:3])[C:4]=[N;+1:5]=[N;-1:6].[Cl;H1:7]>>[C:1][C:2](=[O:3])[C:4]([H:8])[Cl:7].[N;+0:5]#[N;+0:6]

26 [C:1][C:2](=[O:3])[C:4]=[N;+1:5]=[N;-1:6].[Br;H1:7]>>[C:1][C:2](=[O:3])[C:4]([H:8])[Br:7].[N;+0:5]#[N;+0:6]

27 [C;!H0:1][C:2]=[O:3]>>[C:1]=[C:2][O:3][H:4]

28 [C:1]=[C:2][O;H1:3].[Br:4][Br:5]>>[C:1]([Br:4])[C:2]=[O:3].[Br:5][H:6]

29 [C;!H0:1][C:2]([c:4]1[c:5][c:6][c:7][c:8][c:9]1)=[O:3]>>[C:1]=[C:2]([c:4]1[c:5][c:6][c:7][c:8][c:9]1)[O:3][H:10]

30 [C:1]=[C:2]([c:4]1[c:5][c:6][c:7][c:8][c:9]1)[O;H1:3].[Br:10][Br:11]>>[C:1]([Br:10])[C:2]([c:4]1[c:5][c:6][c:7][c:8][c:9]1)=[O:3].[Br:11][H:12]

31 [C;!H0:1][C:2]([O:4])=[O:3]>>[C:1]=[C:2]([O:4])[O:3][H:5]

32 [C:1]=[C:2]([O:4])[O;H1:3].[Br:5][Br:6]>>[C:1]([Br:5])[C:2]([O:4])=[O:3].[Br:6][H:7]

33 [C;!H0:1]([Br:5])[C:2]([O:4])=[O:3]>>[C:1]([Br:5])=[C:2]([O:4])[O:3][H:6]

34 [C:1]([Br:5])=[C:2]([O:4])[O;H1:3].[Br:6][Br:7]>>[C:1]([Br:5])([Br:6])[C:2]([O:4])=[O:3].[Br:7][H:8]

35 [C;!H0:1][C:2](=[O:3])[O:4][C:8].[O;H1:5][N:6]=[O:7]>>[C:1]([C:2](=[O:3])[O:4][C:8])[N:6]=[O:7].[H:9][O:5][H:10]

36 [C;!H0:1][C:2]=[O:3].[O;H1:4][N:5]=[O:6]>>[C:1]([13C:2]=[17O:3])[N:5]=[O:6].[H:7][O:4][H:8]

37 [C;!H0:1][C:2](=[O:3])[N:4]([C:8])[C:9].[O;H1:5][N:6]=[O:7]>>[C:1]([C:2](=[O:3])[N:4]([C:8])[C:9])[N:6]=[O:7].[H:10][O:5][H:11]

38 [C;!H0:1][C:2]=[O:3].[C:4]=[C:5][O;H1:6]>>[C:1]([C:2]=[O:3])[13C:4][C:5]=[O:6].[H:7][H:8]

39 [C;!H0:1][C:2](=[O:3])[O:4][c:8]1[c:9][c:10][c:11][c:12][c:13]1.[O;H1:5][N:6]=[O:7]>>[C:1]([C:2](=[O:3])[O:4][c:8]1[c:9][c:10][c:11][c:12][c:13]1)[N:6]=[O:7].[H:14][O:5][H:15]

40 [C;!H0:1][C:2](=[O:3])[c:7]1[c:8][c:9][c:10][c:11][c:12]1.[O;H1:4][N:5]=[O:6]>>[C:1]([C:2](=[O:3])[c:7]1[c:8][c:9][c:10][c:11][c:12]1)[N:5]=[O:6].[H:13][O:4][H:14]

41 [C;!H0:1][C:2](=[O:3])[N:4]([c:8]1[c:9][c:10][c:11][c:12][c:13]1)[c:14]2[c:15][c:16][c:17][c:18][c:19]2.[O;H1:5][N:6]=[O:7]>>[C:1]([C:2](=[O:3])[N:4]([c:8]1[c:9][c:10][c:11][c:12][c:13]1)[c:14]2[c:15][c:16][c:17][c:18][c:19]2)[N:6]=[O:7].[H:20][O:5][H:21]

42 [C;!$(C=O);!$(CO):1][N;H1:2][C;!$(C=O);!$(CO):3].[Cl:10][c:4]1[c:5][c:6][c:7][c:8][c:9]1>>[C:1][N:2]([C:3])[c:4]1[c:5][c:6][c:7][c:8][c:9]1.[Cl:10][H:11]

43 [O;H1:1][N;+1:2](=[O:3])[O;-1:4].[O;H1:5][c:6]1[c:7][c:8][c:9][c:10][c:11]1>>[O:1]([N;+1:2](=[O:3])[O;-1:4])[c:6]1[c:7][c:8][c:9][c:10][c:11]1.[H:12][O:5][H:13]

44 [O;H1:1][N;+1:2](=[O:3])[O;-1:4].[O;H1:5][c:6]1[c:7][c:8][c:9][c:10][c:11]1>>[O:5]([N;+1:2](=[O:3])[O;-1:4])[c:6]1[c:7][c:8][c:9][c:10][c:11]1.[H:12][O:1][H:13]

45 [O;H1:1][c:2]1[c:3][c:4][c:5][c:6][c:7]1.[O;H1:8][N;+0:9]=[O;+0:10]>>[N;+1:9]([O;-1:10])(=[O:8])[c:2]1[c:3][c:4][c:5][c:6][c:7]1.[H:11][O:1][H:12]

46 [C:1][N:2]([C:3])[c:4]1[c:5][c:6][c;H1:7][c:8][c:9]1.[O;H1:10][N:11]=[O:12]>>[C:1][N:2]([C:3])[c:4]1[c:5][c:6][c:7]([N:11]=[O:12])[c:8][c:9]1.[H:13][O:10][H:14]

47 [O:1][c:2]1[c:3][c:4][c;H1:5][c:6][c:7]1.[O;H1:8][N:9]=[O:10]>>[O:1][c:2]1[c:3][c:4][c:5]([N:9]=[O:10])[c:6][c:7]1.[H:11][O:8][H:12]

48 [c;H1:1]1[c:2][c:3][c:4][c:5][c:6]1.[Cl:7][S:8](=[O:9])(=[O:10])[O;H1:11]>>[Cl:7][S:8](=[O:9])(=[O:10])[c:1]1[c:2][c:3][c:4][c:5][c:6]1.[H:12][O:11][H:13]

49 [c;H1:1]1[c:2][c:3][c:4][c:5][c:6]1.[Br:7][S:8](=[O:9])(=[O:10])[O;H1:11]>>[Br:7][S:8](=[O:9])(=[O:10])[c:1]1[c:2][c:3][c:4][c:5][c:6]1.[H:12][O:11][H:13]

50 [c;H1:1]1[c:2][c:3][c:4][c:5][c:6]1.[Br:7][Br:8]>>[Br:7][c:1]1[c:2][c:3][c:4][c:5][c:6]1.[Br:8][H:9]

51 [c;H1:1]1[c:2][c:3][c:4][c:5][c:6]1.[c:7]2[c:8][c:9][c:10][c:11][c:12]2[S:13](=[O:14])(=[O:15])[O;H1:16]>>[c:7]1[c:8][c:9][c:10][c:11][c:12]1[S:13](=[O:14])(=[O:15])[c:1]2[c:2][c:3][c:4][c:5][c:6]2.[H:17][O:16][H:18]

52 [c;H1:1]1[c:2][c:3][c:4][c:5][c:6]1.[Cl:7][Cl:8]>>[Cl:7][c:1]1[c:2][c:3][c:4][c:5][c:6]1.[Cl:8][H:9]

53 [O;H1:1][N;+1:2](=[O:3])[O;-1:4].[c;H1:5]1[c:6][c:7][c:8][c:9][c:10]1>>[N;+1:2](=[O:3])([O;-1:4])[c:5]1[c:6][c:7][c:8][c:9][c:10]1.[H:11][O:1][H:12]

54 [c:1]1[c:2][c:3][c:4][c:5][c;H1:6]1.[c:7]2[c:8][c:9][c:10][c:11][c:12]2[N:13]([C:14])[C:15]=[O:16]>>[c:1]1[c:2][c:3][c:4][c:5][c:6]1[C:15]=[O:16].[c:7]2[c:8][c:9][c:10][c:11][c:12]2[N:13]([C:14])[H:17]

55 [c:1]1[c:2][c:3][c:4][c:5][c:6]1[C:7][O;H1:8].[c;H1:9]2[c:10][c:11][c:12][c:13][c:14]2>>[c:1]1[c:2][c:3][c:4][c:5][c:6]1[C:7][c:9]2[c:10][c:11][c:12][c:13][c:14]2.[H:15][O:8][H:16]

56 [c:1]1[c:2][c:3][c:4]2[c:5]([c:6]1)[c:7][c:8][c:9][c:10]2[O;H1:11].[N;!H0:12]>>[c:1]1[c:2][c:3][c:4]2[c:5]([c:6]1)[c:7][c:8][c:9][c:10]2[N:12].[H:13][O:11][H:14]

57 [c:1]1([O;H1:11])[c:2][c:3][c:4]2[c:5]([c:6]1)[c:7][c:8][c:9][c:10]2.[N;!H0:12]>>[c:1]1([N:12])[c:2][c:3][c:4]2[c:5]([c:6]1)[c:7][c:8][c:9][c:10]2.[H:13][O:11][H:14]

58 [C;!$(C=O):1][O;H1:2].[C;!$(C=O):3][O;H1:4]>>[C:1][O:2][C:3].[H:5][O:4][H:6]

59 [C:1][O;H1:2].[O;H1:3][N;+1:4](=[O:5])[O;-1:6]>>[C:1][O:2][N;+1:4](=[O:5])[O;-1:6].[H:7][O:3][H:8]

60 [C:1][Cl:2].[C:3]=[N:4][O;H1:5]>>[C:3]=[N:4][O:5][C:1].[Cl:2][H:6]

61 [C:1][Cl:2].[c:11]1[c:10][c:9][c:8][c:7][c:6]1[C:5]([c:12]2[c:13][c:14][c:15][c:16][c:17]2)=[N:4][O;H1:3]>>[c:11]1[c:10][c:9][c:8][c:7][c:6]1[C:5]([c:12]2[c:13][c:14][c:15][c:16][c:17]2)=[N:4][O:3][C:1].[Cl:2][H:18]

62 [C:1][C:2]([O;H1:4])([C:5]#[N:6])[C:3].[N;!H0:7]>>[C:1][C:2]([15N:7])([C:5]#[N:6])[C:3].[H:8][17

O:4][H:9]

63 [C;!$(C=O):1][O;H1:2].[Cl;H1:3]>>[13C:1][36Cl:3].[H:4][O:2][H:5]

64 [C;!$(C=O):1][O;H1:2].[Br;H1:3]>>[13C:1][79Br:3].[H:4][O:2][H:5]

65 [C;!H0:1][C:2]#[N:3].[O;H1:4][N:5]=[O:6]>>[C:1]([C:2]#[N:3])[N:5]=[O:6].[H:7][O:4][H:8]

66 [C;!H0:1][S:2](=[O:3])[C:7].[O;H1:4][N:5]=[O:6]>>[C:1]([S:2](=[O:3])[C:7])[N:5]=[O:6].[H:8][O:4][H:9]

67 [C;!H0:1][S:2](=[O:3])(=[O:4])[C:8].[O;H1:5][N:6]=[O:7]>>[C:1]([S:2](=[O:3])(=[O:4])[C:8])[N:6]=[O:7].[H:9][O:5][H:10]

68 [C;!H0:1][S:2](=[O:3])(=[O:4])[O:5][C:9].[O;H1:6][N:7]=[O:8]>>[C:1]([S:2](=[O:3])(=[O:4])[O:5][C:9])[N:7]=[O:8].[H:10][O:6][H:11]

69 [C;!H0:1][S:2](=[O:3])(=[O:4])[N:5]([C:9])[C:10].[O;H1:6][N:7]=[O:8]>>[C:1]([S:2](=[O:3])(=[O:4])[N:5]([C:9])[C:10])[N:7]=[O:8].[H:11][O:6][H:12]

70 [C;!H0:1][S:2](=[O:3])[c:7]1[c:8][c:9][c:10][c:11][c:12]1.[O;H1:4][N:5]=[O:6]>>[C:1]([S:2](=[O:3])[c:7]1[c:8][c:9][c:10][c:11][c:12]1)[N:5]=[O:6].[H:13][O:4][H:14]

71 [C;!H0:1][S:2](=[O:3])(=[O:4])[c:8]1[c:9][c:10][c:11][c:12][c:13]1.[O;H1:5][N:6]=[O:7]>>[C:1]([S:2](=[O:3])(=[O:4])[c:8]1[c:9][c:10][c:11][c:12][c:13]1)[N:6]=[O:7].[H:14][O:5][H:15]

72 [C;!H0:1][S:2](=[O:3])(=[O:4])[O:5][c:9]1[c:10][c:11][c:12][c:13][c:14]1.[O;H1:6][N:7]=[O:8]>>[C:1]([S:2](=[O:3])(=[O:4])[O:5][c:9]1[c:10][c:11][c:12][c:13][c:14]1)[N:7]=[O:8].[H:15][O:6][H:16]

73 [C;!H0:1][S:2](=[O:3])(=[O:4])[N:5]([c:9]1[c:10][c:11][c:12][c:13][c:14]1)[c:15]2[c:16][c:17][c:18][c:19][c:20]2.[O;H1:6][N:7]=[O:8]>>[C:1]([S:2](=[O:3])(=[O:4])[N:5]([c:9]1[c:10][c:11][c:12][c:13][c:14]1)[c:15]2[c:16][c:17][c:18][c:19][c:20]2)[N:7]=[O:8].[H:21][O:6][H:22]

74 [C;!H0:1][Cl:2].[O;H1:3][c:4][c:5][c:6][c:7][c:8][c:9]>>[C:1][O:3][c:4][c:5][c:6][c:7][c:8][c:9].[Cl:2][H:10]

75 [C;!H0:1][Cl:2].[O;H1:3][C:4]>>[C:1][O:3][C:4].[Cl:2][H:5]

76 [c:10]1[c:9][c:8][c:7][c:6][c:1]1[N:2]([C;!$(C=O);!$(CO):3])[C;!$(C=O);!$(CO):4].[Br;H1:5]>>[c:10]1[c:9][c:8][c:7][c:6][c:1]1[N:2]([H:11])[C:4].[C:3][Br:5]

77 [S:1](=[O:2])(=[O:3])([Cl:4])[Cl:5].[N;!H0:6]>>[S:1](=[O:2])(=[O:3])([Cl:4])[N:6].[Cl:5][H:7]

78 [S:1](=[O:2])(=[O:3])([Cl:4])[N:5].[N;!H0:6]>>[S:1](=[O:2])(=[O:3])([N:6])[N:5].[Cl:4][H:7]

79 [C:1][N;H1:2][C:3].[O;H1:4][N:5]=[O:6]>>[C:1][N:2]([N:5]=[O:6])[C:3].[H:7][O:4][H:8]

80 [S:1](=[O:2])(=[O:3])([Cl:4])[Cl:5].[O;!H0:6]>>[S:1](=[O:2])(=[O:3])([Cl:4])[O:6].[Cl:5][H:7]

81 [S:1](=[O:2])(=[O:3])([Cl:4])[O:5].[O;!H0:6]>>[S:1](=[O:2])(=[O:3])([O:6])[O:5].[Cl:4][H:7]

82 [S:1](=[O:2])(=[O:3])([O:4][C:5])[O:6][C:7].[O;!H0:8]>>[S:1](=[O:2])(=[O:3])([O:4][C:5])[O:8].[H:9][17O:6][13C:7]

83 [S:1](=[O:2])(=[O:3])([O:4][C:5])[O:6].[O;!H0:7]>>[S:1](=[O:2])(=[O:3])([O:7])[O:6].[H:8][O:4][C:5]

84 [S:1](=[O:2])(=[O:3])([N:4])[N;H2:5].[O;!H0:6]>>[S:1](=[O:2])(=[O:3])([N:4])[O:6].[N:5]([H:7])([H:8])[H:9]

85 [S:1](=[O:2])(=[O:3])([N;H2:4])[O:5].[O;!H0:6]>>[S:1](=[O:2])(=[O:3])([O:6])[O:5].[N:4]([H:7])([H:8])[H:9]

86 [S:1](=[O:2])(=[O:3])([O:4])[O:5].[Cl:7][P:6]([Cl:8])([Cl:9])([Cl:10])[Cl:11]>>[S:1](=[O:2])(=[O:3])([O:4])[Cl:7].[O:5][P:6]([Cl:8])([Cl:9])([Cl:10])[Cl:11]

87 [S:1](=[O:2])(=[O:3])([O:4])[Cl:5].[Cl:7][P:6]([Cl:8])([Cl:9])([Cl:10])[Cl:11]>>[S:1](=[O:2])(=[O:3])([Cl:7])[Cl:5].[O:4][P:6]([Cl:8])([Cl:9])([Cl:10])[Cl:11]

88 [C:1]=[O:2].[O;H2:3]>>[H:4][O:2][C:1][O:3][H:5]

89 [C:3][C;H1:1]=[O:2].[C:4][C:5](=[O:6])[O:7][C:8](=[O:9])[C:10]>>[C:3][C:1](=[O:2])[O:6][C:5]([C:4])([H:11])[O:7][C:8](=[O:9])[C:10]

90 [C:1]=[C:2].[Cl;H1:3]>>[H:4][C:1][C:2][Cl:3]

91 [C:1]=[C:2].[C:3][O;H1:4]>>[H:5][C:1][C:2][O:4][C:3]

92 [C:1]=[C:2].[C:3][C:4](=[O:5])[O;H1:6]>>[H:7][C:1][C:2][O:6][C:4](=[O:5])[C:3]

93 [C:1]=[C:2].[S;H2:3]>>[H:4][C:1][C:2][S;H1:3]

94 [C:1]=[C:2].[S;H1:3][c:4]1[c:5][c:6][c:7][c:8][c:9]1>>[H:10][C:1][C:2][S:3][c:4]1[c:5][c:6][c:7][c:8][c:9]1

95 [C:1]=[C:2].[C:3][N;H1:4][C:5](=[O:6])[C:7]>>[H:8][C:1][C:2][N:4]([C:3])[C:5](=[O:6])[C:7]

96 [C:1]=[C:2].[c:17]2[c:16][c:15][c:14][c:13][c:12]2[N;H1:3][C:4](=[O:5])[c:6]1[c:7][c:8][c:9][c:10][c:11]1>>[H:18][C:1][C:2][N:3]([c:12]2[c:13][c:14][c:15][c:16][c:17]2)[C:4](=[O:5])[c:6]1[c:7][c:8][c:9][c:10][c:11]1

97 [C:1]=[C:2].[C:3][C;H1:4](=[O:5])>>[H:6][C:1][C:2][C:4](=[O:5])[C:3]

98 [C:1]=[C:2].[c:10]1[c:9][c:8][c:7][c:6][c:5]1[C;H1:3](=[O:4])>>[H:11][C:1][C:2][C:3](=[O:4])[c:5]1[c:6][c:7][c:8][c:9][c:10]1

99 [C:1]=[C:2].[C:6][O:3][C;H1:4](=[O:5])>>[H:7][C:1][C:2][C:4](=[O:5])[O:3][C:6]

100 [C:1]=[C:2].[N:3][C;H1:4](=[O:5])>>[H:6][C:1][C:2][C:4](=[O:5])[N:3]

101 [C:1]=[C:2].[C;H1:3]#[N:4]>>[H:5][C:1][C:2][C:3]#[N:4]

102 [C:1]=[C:2].[Cl:3][Cl:4]>>[Cl:3][C:1][C:2][Cl:4]

103 [C:1]=[C:2].[C:3][S:4](=[O:5])(=[O:6])[Cl:7]>>[Cl:7][C:1][C:2][S:4](=[O:5])(=[O:6])[C:3]

104 [C:1]=[C:2].[Cl:3][S:4](=[O:5])[Cl:6]>>[Cl:3][C:1][C:2][S:4](=[O:5])[Cl:6]

105 [C:1]=[C:2].[C:3][S:4][Cl:5]>>[C:3][C:1][C:2][S:4][Cl:5]

106 [C:1]=[C:2].[O:3]=[N:4][Cl:5]>>[Cl:5][C:1][C:2][N:4]=[O:3]

107 [C:1]=[C:2].[c:11]1[c:10][c:9][c:8][c:7][c:6]1[C:3](=[O:4])[O;H1:5]>>[H:12][C:1][C:2][O:5][C:3](=[O:4])[c:6]1[c:7][c:8][c:9][c:10][c:11]1

108 [C:1]=[C:2].[c:11]1[c:10][c:9][c:8][c:7][c:6]1[O:3][C;H1:4](=[O:5])>>[H:12][C:1][C:2][C:4](=[O:5])[O:3][c:6]1[c:7][c:8][c:9][c:10][c:11]1

109 [C:1]=[C:2].[Br;H1:3]>>[H:4][C:1][C:2][Br:3]

110 [C:1]=[C:2].[Br:3][Br:4]>>[Br:3][C:1][C:2][Br:4]

111 [C:1]=[C:2].[C:3][S:4](=[O:5])(=[O:6])[Br:7]>>[Br:7][C:1][C:2][S:4](=[O:5])(=[O:6])[C:3]

112 [C:1]=[C:2].[Br:3][S:4](=[O:5])[Br:6]>>[Br:3][C:1][C:2][S:4](=[O:5])[Br:6]

113 [C:1]=[C:2].[C:3][S:4][Br:5]>>[C:3][C:1][C:2][S:4][Br:5]

114 [C:1]=[C:2].[c:12]1[c:11][c:10][c:9][c:8][c:7]1[S:3](=[O:4])(=[O:5])[Cl:6]>>[Cl:6][C:1][C:2][S:3](=[O:4])(=[O:5])[c:7]1[c:8][c:9][c:10][c:11][c:12]1

115 [C:1]=[C:2].[c:10]1[c:9][c:8][c:7][c:6][c:5]1[S:3][Cl:4]>>[c:10]1[c:9][c:8][c:7][c:6][c:5]1[C:1][C:2][S:3][Cl:4]

116 [C:1]=[C:2].[O:3]=[N:4][Br:5]>>[Br:5][C:1][C:2][N:4]=[O:3]

117 [C:1]#[C:2].[C:3][S:4](=[O:5])(=[O:6])[Cl:7]>>[Cl:7][C:1]=[C:2][S:4](=[O:5])(=[O:6])[C:3]

118 [C:1]#[C:2].[C:3][S:4](=[O:5])(=[O:6])[Br:7]>>[Br:7][C:1]=[C:2][S:4](=[O:5])(=[O:6])[C:3]

119 [C:1]#[C:2].[c:12]1[c:11][c:10][c:9][c:8][c:7]1[S:3](=[O:4])(=[O:5])[Cl:6]>>[Cl:6][C:1]=[C:2][S:3](=[O:4])(=[O:5])[c:7]1[c:8][c:9][c:10][c:11][c:12]1

120 [C:1][N;H2:2].[C;-1:3]#[O;+1:4]>>[C:1][N;H1:2][C;+0:3]([H:5])=[O;+0:4]

121 [c:9]1[c:8][c:7][c:6][c:5][c:4]1[N;H2:1].[C;-1:2]#[O;+1:3]>>[c:9]1[c:8][c:7][c:6][c:5][c:4]1[N;H1:1][C;+0:2]([H:10])=[O;+0:3]

122 [c:1]1[c:2][c:3][c:4][c:5][c;H1:6]1.[C:7][N:8]=[C:9]=[O:10]>>[c:1]1[c:2][c:3][c:4][c:5][c:6]1[C:9](=[O:10])[N:8]([H:11])[C:7]

123 [c:1]1[c:2][c:3][c:4][c:5][c;H1:6]1.[c:15]2[c:14][c:13][c:12][c:11][c:10]2[N:7]=[C:8]=[O:9]>>[c:1]1[c:2][c:3][c:4][c:5][c:6]1[C:8](=[O:9])[N:7]([H:16])[c:10]2[c:11][c:12][c:13][c:14][c:15]2

124 [N;H1:1]=[C:2]=[O:3].[N;!H0:4]>>[H:5][N:1]([H:6])[C:2](=[O:3])[N:4]

125 [N;H1:1]=[C:2]=[S:3].[N;!H0:4]>>[H:5][N:1]([H:6])[C:2](=[S:3])[N:4]

126 [c:10]1[c:9][c:8][c:7][c:6][c:5]1[N:1]=[C:2]=[O:3].[N;H1:4]([c:17]3[c:18][c:19][c:20][c:21][c:223)[c:11]2[c:12][c:13][c:14][c:15][c:16]2>>[c:10]1[c:9][c:8][c:7][c:6][c:5]1[N:1]([H:23])[C:2](=[O:3])[N:4]([c:17]3[c:18][c:19][c:20][c:21][c:22]3)[c:11]2[c:12][c:13][c:14][c:15][c:16]2

127 [c:10]1[c:9][c:8][c:7][c:6][c:5]1[N:1]=[C:2]=[S:3].[N;H1:4]([c:17]3[c:18][c:19][c:20][c:21][c:22]3)[c:11]2[c:12][c:13][c:14][c:15][c:16]2>>[c:10]1[c:9][c:8][c:7][c:6][c:5]1[N:1]([H:23])[C:2](=[S:3])[N:4]([c:17]3[c:18][c:19][c:20][c:21][c:22]3)[c:11]2[c:12][c:13][c:14][c:15][c:16]2

128 [Cl:2][C:1][C:3][O;H1:4]>>[C:1]1[C:3][O:4]1.[Cl:2][H:5]

129 [Cl:2][C:1][C:3][C:5][O;H1:4]>>[C:1]1[C:3][C:5][O:4]1.[Cl:2][H:6]

130 [Cl:2][C:1][C:3][C:5][C:6][O;H1:4]>>[C:1]1[C:3][C:5][C:6][O:4]1.[Cl:2][H:7]

131 [Cl:2][C:1].[C:3][C:5][C:6][C:7][O;H1:4]>>[C:1]1[C:3][C:5][C:6][C:7][O:4]1.[Cl:2][H:8]

132 [C:1]1[O:2][C:3]1.[C:4][O;H1:5]>>[C:1]([O:2][H:6])[C:3][O:5][C:4]

133 [C:1]1[N;H1:2][C:3]1.[C:4][O;H1:5]>>[C:1]([N:2]([H:6])([H:7]))[C:3][O:5][C:4]

134 [C:1]1[O:2][C:3]1.[N;!H0:4]>>[C:1]([O:2][H:5])[C:3][N:4]

135 [C:1][C:2]1[C:3][C:4][O:5]1.[C:6][N;H1:7][C:8]>>[C:1][C:2]([O:5][H:9])[C:3][C:4][N:7]([C:6])[C:8]

136 [C:2][C:1]1[C;H1:3]([C:4])[N:5]1[C:6].[C:7][N;H1:8][C:9]>>[C:2][C:1]([N:5]([H:10])[C:6])[C:3]([N:8]([C:7])[C:9])[C:4]

137 [C;!$(C=O):1]1[O:2][C;!$(C=O):3]1.[F;H1:4]>>[H:5][O:2][C:1][C:3][F:4]

138 [C;!$(C=O):1]1[O:2][C;!$(C=O):3]1.[Br;H1:4]>>[H:5][O:2][C:1][C:3][Br:4]

139 [C:1]=[C:2][C:3]=[C:4].[C:5]#[C:6]>>[C:1]1[C:2]=[C:3][C:4][C:5]=[C:6]1

140 [C:1]=[C:2][C:3]=[C:4].[N:5]#[C:6]>>[C:1]1[C:2]=[C:3][C:4][N:5]=[C:6]1

141 [C:1]=[C:2][C:3]=[C:4].[N:5]=[C:6]>>[C:1]1[C:2]=[C:3][C:4][N:5][C:6]1

142 [C:1]=[C:2][C:3]=[C:4].[N:5]=[N:6]>>[C:1]1[C:2]=[C:3][C:4][N:5][N:6]1

143 [C:1]=[C:2][C:3]=[C:4].[O:5]=[N:6]>>[C:1]1[C:2]=[C:3][C:4][O:5][N:6]1

144 [C:1]=[C:2][C:3]=[C:4].[O:5]=[C:6]>>[C:1]1[C:2]=[C:3][C:4][O:5][C:6]1

145 [C:1][C:2](=[O:3])[C:4].[C:5][C:6](=[C:7]=[O:8])[C:9]>>[C:1][13C:2]1([C:4])[17O:3][C:7](=[O:8])[C:6]1([C:5])[C:9]

146 [C:1]=[O:2].[C:3]=[C:4]>>[C:1]1[O:2][C:3][C:4]1

147 [C:1][C:2](=[N:3])[C:4].[C:5][C:6](=[C:7]=[O:8])[C:9]>>[C:1][C:2]1([C:4])[N:3][C:7](=[O:8])[C:6]1([C:5])[C:9]

148 [C:1][C:2]1[C:3]=[C:4][C:5]1[C:6]>>[C:1][C:2]=[C:3][C:4]=[C:5][C:6]

149 [C:1][C:2]1[C:3]=[C:4][C:5]=[C:6][C:7]1[C:8]>>[C:1][C:2]=[C:3][C:4]=[C:5][C:6]=[C:7][C:8]

150 [C:1]=[C:2].[C:3][C:4](=[O:5])[O:6][O;H1:7]>>[C:1]1[C:2][O:7]1.[C:3][C:4](=[O:6])[O:5][H:8]

151 [C:1][O:2][C:3](=[O:4])[C;H1:5]([C:6])[Cl:7]>>[C:1][O:2][C:3]([O:4][H:8])=[C:5]([C:6])[Cl:7]

152 [C;H1:1]=[N:2][O;H1:3]>>[C:1]#[N:2].[H:4][O:3][H:5]

153 [O:3]=[C:1][N;!H0:2]>>[H:4][O:3][C:1]=[N:2]

154 [O;H1:3][C:1]=[N;H1:2]>>[C:1]#[N:2].[H:4][O:3][H:5]

155 [C:1]=[C:2]([O;H1:3])[C:4]>>[H:5][C:1][C:2](=[O:3])[C:4]

156 [c:1]1[c:2][c:3][c:4][c;H1:5][c:6]1[O:7][C:8](=[O:9])[C:10]>>[C:1]1=[C:2][C:3]=[C:4][C:5]([H:11])([

C:8](=[O:9])[C:10])[C:6]1=[O:7]

157 [C:1]1=[C:2][C:3]=[C:4][C;H1:5]([C:8](=[O:9])[C:10])[C:6]1=[O:7]>>[c:1]1[c:2][c:3][c:4][c:5]([C:8](=[O:9])[C:10])[c:6]1[O:7][H:11]

158 [c:1]1[c:2][c:3][c:4][c;H1:5][c:6]1[O:7][C:8](=[O:9])[c:10]2[c:11][c:12][c:13][c:14][c:15]2>>[C:1]1=[C:2][C:3]=[C:4][C:5]([H:16])([C:8](=[O:9])[c:10]2[c:11][c:12][c:13][c:14][c:15]2)[C:6]1=[O:7]

159 [C:1]1=[C:2][C:3]=[C:4][C;H1:5]([C:8](=[O:9])[c:10]2[c:11][c:12][c:13][c:14][c:15]2)[C:6]1=[O:7]>>[c:1]1[c:2][c:3][c:4][c:5]([C:8](=[O:9])[c:10]2[c:11][c:12][c:13][c:14][c:15]2)[c:6]1[O:7][H:16]

160 [C:1]=[C:2][C;H1:3][C:4]>>[H:5][C:1][C:2]=[C:3][C:4]

161 [C:1]=[C:2]([O;H1:3])[c:4]1[c:5][c:6][c:7][c:8][c:9]1>>[H:10][C:1][C:2](=[O:3])[c:4]1[c:5][c:6][c:7][c:8][c:9]1

162 [C:1][C:2]([O;H1:4])=[N:3][C:5]>>[C:1][C:2](=[O:4])[N:3]([H:6])[C:5]

163 [C:1]=[C:2][C:3]([C:4])[O:5][c:6]1[c:7][c:8][c:9][c:10][c;H1:11]1>>[C:1]([C:2]=[C:3][C:4])[C:11]1([H:12])[C:10]=[C:9][C:8]=[C:7][C:6]1=[O:5]

164 [C:1]=[C:2][C:3][O:4][C:5]([C:6])=[C:7][C:8][C:9]>>[C:6][C:5](=[O:4])[C:7]([C:8][C:9])[C:1][C:2]=[C:3]

165 [c:21]3[c:20][c:19][c:18][c:17][c:16]3[O:1][C:2]([c:10]2[c:11][c:12][c:13][c:14][c:15]2)=[N:3][c:4]1[c:5][c:6][c:7][c:8][c:9]1>>[c:21]3[c:20][c:19][c:18][c:17][c:16]3[C:2](=[O:1])[N:3]([c:10]2[c:11][c:12][c:13][c:14][c:15]2)[c:4]1[c:5][c:6][c:7][c:8][c:9]1

166 [c:1]1[c:2][c:3][c:4][c:5][c:6]1[O:7][C:8][C:9]([C:11])=[C:10]>>[C:1]1=[C:2][C:3]=[C:4][C:5]([C:10][C:9]([C:11])=[C:8])[C:6]1=[O:7]

167 [C:1]1=[C:2][C:3]=[C:4][C;H1:5]([C:10][C:9]([C:11])=[C:8])[C:6]1=[O:7]>>[c:1]1[c:2][c:3][c:4][c:5]([C:10][C:9]([C:11])=[C:8])[c:6]1[O:7][H:12]

168 [c:1]1[c:2][c:3][c:4][c:5]([C:10][C:9]([C:11])=[C:8])[c:6]1[O;H1:7]>>[c:1]1[c:2][c:3][c:4][c:5]2[c:6]1[O:7][C:9]([C:8][H:12])([C:11])[C:10]2

169 [c:1]1[c:2][c:3][c:4][c;H1:5][c:6]1[O:7][C:8][C:9]([C:11])=[C:10]>>[c:1]1[c:2][c:3][c:4][c:5]2[c:6]1[O:7][C:8][C:9]2([C:10][H:12])[C:11]

170 [C:1][C:2]=[C:3]=[O:4].[O;H2:5]>>[C:1][C:2]([H:6])[C:3](=[O:4])[O;H1:5]

171 [c:10]1[c:9][c:8][c:7][c:6][c:5]1[C:3]=[C:1]=[O:2].[O;H2:4]>>[c:10]1[c:9][c:8][c:7][c:6][c:5]1[C:3]([H:11])[C:1](=[O:2])[O;H1:4]

172 [C:1][N;+1:2]([N:3]=[N;-1:4])=[C:5][O:6]>>[C:1][N;+0:2]=[C:5]=[O:6].[N:3]#[N;+0:4]

173 [C:1]=[C:2].[O:3]=[N:4][Cl:5]>>[C:1]([Cl:5])[C:2][N:4]=[O:3]

174 [C:1]#[N:2].[O;H2:3]>>[O;H1:3][C:1]=[N:2][H:4]

175 [O;H1:3][C:1]=[N;H1:2]>>[O:3]=[C:1][N:2]([H:4])([H:5])

176 [C:1][C:2](=[O:3]).[N;H2:4][O:5]>>[C:1][C:2]([O:3][H:6])[N;H1:4][O:5]

177 [C:1][C:2]([O;H1:3])[N;H1:4][O:5]>>[C:1][C:2]=[N:4][O:5].[H:6][O:3][H:7]

178 [c:10]1[c:9][c:8][c:7][c:6][c:5]1[C:1](=[O:2]).[N;H2:3][O:4]>>[c:10]1[c:9][c:8][c:7][c:6][c:5]1[C:1]([O:2][H:11])[N;H1:3][O:4]

179 [c:10]1[c:9][c:8][c:7][c:6][c:5]1[C:1]([O;H1:2])[N;H1:3][O:4]>>[c:10]1[c:9][c:8][c:7][c:6][c:5]1[C:1]=[N:3][O:4].[H:11][O:2][H:12]

180 [O:1]=[N:2][O;H1:3].[C;!H0:4][C:5](=[O:6])[O:7][C:8]>>[O:1]=[N:2][C:4][C:5](=[O:6])[O:7][C:8].[H:9][O:3][H:10]

181 [O:1]=[N:2][C;!H0:3][C:4](=[O:5])[O:6][C:7]>>[H:8][O:1][N:2]=[C:3][C:4](=[O:5])[O:6][C:7]

182 [O:1]=[N:2][O:3].[C;H3:4][C:5](=[O:6])[O:7][C:8]>>[O:3][N:2]([O:1][H:9])[C;H2:4][C:5](=[O:6])[O:7][C:8]

183 [O;H1:3][N:2]([O;H1:1])[C:4][C:5](=[O:6])[O:7][C:8]>>[O:3]=[N:2][C:4][C:5](=[O:6])[O:7][C:8].[H:9][O:1][H:10]

184 [O:3][N:2]([O;H1:1])[C;!H0:4][C:5](=[O:6])[O:7][C:8]>>[O:3][N:2]=[C:4][C:5](=[O:6])[O:7][C:8].[H:9][O:1][H:10]

185 [O:1]=[N:2][O;H1:3].[C;!H0:4][C:5](=[O:6])[O:7][c:8]1[c:9][c:10][c:11][c:12][c:13]1>>[O:1]=[N:2][C:4][C:5](=[O:6])[O:7][c:8]1[c:9][c:10][c:11][c:12][c:13]1.[H:14][O:3][H:15]

186 [O:1]=[N:2][C;!H0:3][C:4](=[O:5])[O:6][c:7]1[c:8][c:9][c:10][c:11][c:12]1>>[H:13][O:1][N:2]=[C:3][C:4](=[O:5])[O:6][c:7]1[c:8][c:9][c:10][c:11][c:12]1

187 [O:1]=[N:2][O:3].[C;H3:4][C:5](=[O:6])[O:7][c:8]1[c:9][c:10][c:11][c:12][c:13]1>>[O:3][N:2]([O:1][H:14])[C;H2:4][C:5](=[O:6])[O:7][c:8]1[c:9][c:10][c:11][c:12][c:13]1

188 [O;H1:3][N:2]([O;H1:1])[C:4][C:5](=[O:6])[O:7][c:8]1[c:9][c:10][c:11][c:12][c:13]1>>[O:3]=[N:2][C:4][C:5](=[O:6])[O:7][c:8]1[c:9][c:10][c:11][c:12][c:13]1.[H:14][O:1][H:15]

189 [O:3][N:2]([O;H1:1])[C;!H0:4][C:5](=[O:6])[O:7][c:8]1[c:9][c:10][c:11][c:12][c:13]1>>[O:3][N:2]=[C:4][C:5](=[O:6])[O:7][c:8]1[c:9][c:10][c:11][c:12][c:13]1.[H:14][O:1][H:15]

190 [O:1]=[N:2][O;H1:3].[C;!H0:4][C:5]=[O:6]>>[O:1]=[N:2][C:4][C:5]=[O:6].[H:7][O:3][H:8]

191 [O:1]=[N:2][C;!H0:3][C:4](=[O:5])>>[H:6][O:1][N:2]=[C:3][C:4](=[O:5])

192 [O:1]=[N:2][O:3].[C;H3:4][C:5](=[O:6])>>[O:3][N:2]([O:1][H:7])[C;H2:4][C:5](=[O:6])

193 [O;H1:3][N:2]([O;H1:1])[C:4][C:5](=[O:6])>>[O:3]=[N:2][C:4][C:5](=[O:6]).[H:7][O:1][H:8]

194 [O:3][N:2]([O;H1:1])[C;!H0:4][C:5](=[O:6])>>[O:3][N:2]=[C:4][C:5](=[O:6]).[H:7][O:1][H:8]

195 [O:1]=[N:2][O;H1:3].[C;!H0:4][C:5](=[O:6])[c:7]1[c:8][c:9][c:10][c:11][c:12]1>>[O:1]=[N:2][C:4][C:5](=[O:6])[c:7]1[c:8][c:9][c:10][c:11][c:12]1.[H:13][O:3][H:14]

196 [O:1]=[N:2][C;!H0:3][C:4](=[O:5])[c:6]1[c:7][c:8][c:9][c:10][c:11]1>>[H:12][O:1][N:2]=[C:3][C:4](=[O:5])[c:6]1[c:7][c:8][c:9][c:10][c:11]1

197 [O:1]=[N:2][O:3].[C;H3:4][C:5](=[O:6])[c:7]1[c:8][c:9][c:10][c:11][c:12]1>>[O:3][N:2]([O:1][H:13])[C;H2:4][C:5](=[O:6])[c:7]1[c:8][c:9][c:10][c:11][c:12]1

198 [O;H1:3][N:2]([O;H1:1])[C:4][C:5](=[O:6])[c:7]1[c:8][c:9][c:10][c:11][c:12]1>>[O:3]=[N:2][C:4][C:5](=[O:6])[c:7]1[c:8][c:9][c:10][c:11][c:12]1.[H:13][O:1][H:14]

199 [O:3][N:2]([O;H1:1])[C;!H0:4][C:5](=[O:6])[c:7]1[c:8][c:9][c:10][c:11][c:12]1>>[O:3][N:2]=[C:4][C:5](=[O:6])[c:7]1[c:8][c:9][c:10][c:11][c:12]1.[H:13][O:1][H:14]

200 [O:1]=[N:2][O;H1:3].[C;!H0:4][C:5](=[O:6])[N:7]([C:8])[C:9]>>[O:1]=[N:2][C:4][C:5](=[O:6])[N:7]([C:8])[C:9].[H:10][O:3][H:11]

201 [O:1]=[N:2][C;!H0:3][C:4](=[O:5])[N:6]([C:7])[C:8]>>[H:9][O:1][N:2]=[C:3][C:4](=[O:5])[N:6]([C:7])[C:8]

202 [O:1]=[N:2][O:3].[C;H3:4][C:5](=[O:6])[N:7]([C:8])[C:9]>>[O:3][N:2]([O:1][H:10])[C;H2:4][C:5](=[O:6])[N:7]([C:8])[C:9]

203 [O;H1:3][N:2]([O;H1:1])[C:4][C:5](=[O:6])[N:7]([C:8])[C:9]>>[O:3]=[N:2][C:4][C:5](=[O:6])[N:7]([C:8])[C:9].[H:10][O:1][H:11]

204 [O:3][N:2]([O;H1:1])[C;!H0:4][C:5](=[O:6])[N:7]([C:8])[C:9]>>[O:3][N:2]=[C:4][C:5](=[O:6])[N:7]([C:8])[C:9].[H:10][O:1][H:11]

205 [O:1]=[N:2][O;H1:3].[C;!H0:4][S:5](=[O:6])[C:7]>>[O:1]=[N:2][C:4][S:5](=[O:6])[C:7].[H:8][O:3][H:9]

206 [O:1]=[N:2][C;!H0:3][S:4](=[O:5])[C:6]>>[H:7][O:1][N:2]=[C:3][S:4](=[O:5])[C:6]

207 [O:1]=[N:2][O:3].[C;H3:4][S:5](=[O:6])[C:7]>>[O:3][N:2]([O:1][H:8])[C;H2:4][S:5](=[O:6])[C:7]

208 [O;H1:3][N:2]([O;H1:1])[C:4][S:5](=[O:6])[C:7]>>[O:3]=[N:2][C:4][S:5](=[O:6])[C:7].[H:8][O:1][H:

9]

209 [O:3][N:2]([O;H1:1])[C;H2:4][S:5](=[O:6])[C:7]>>[O:3][N:2]=[C;H1:4][S:5](=[O:6])[C:7].[H:8][O:1][H:9]

210 [O:1]=[N:2][O;H1:3].[C;!H0:4][S:5](=[O:6])[c:7]1[c:8][c:9][c:10][c:11][c:12]1>>[O:1]=[N:2][C:4][S:5](=[O:6])[c:7]1[c:8][c:9][c:10][c:11][c:12]1.[H:13][O:3][H:14]

211 [O:1]=[N:2][C;!H0:3][S:4](=[O:5])[c:6]1[c:7][c:8][c:9][c:10][c:11]1>>[H:12][O:1][N:2]=[C:3][S:4](=[O:5])[c:6]1[c:7][c:8][c:9][c:10][c:11]1

212 [O:1]=[N:2][O:3].[C;H3:4][S:5](=[O:6])[c:7]1[c:8][c:9][c:10][c:11][c:12]1>>[O:3][N:2]([O:1][H:13])[C;H2:4][S:5](=[O:6])[c:7]1[c:8][c:9][c:10][c:11][c:12]1

213 [O;H1:3][N:2]([O;H1:1])[C:4][S:5](=[O:6])[c:7]1[c:8][c:9][c:10][c:11][c:12]1>>[O:3]=[N:2][C:4][S:5](=[O:6])[c:7]1[c:8][c:9][c:10][c:11][c:12]1.[H:13][O:1][H:14]

214 [O:3][N:2]([O;H1:1])[C;!H0:4][S:5](=[O:6])[c:7]1[c:8][c:9][c:10][c:11][c:12]1>>[O:3][N:2]=[C:4][S:5](=[O:6])[c:7]1[c:8][c:9][c:10][c:11][c:12]1.[H:13][O:1][H:14]

215 [O:1]=[N:2][O;H1:3].[C;!H0:4][S:5](=[O:6])(=[O:7])[C:8]>>[O:1]=[N:2][C:4][S:5](=[O:6])(=[O:7])[C:8].[H:9][O:3][H:10]

216 [O:1]=[N:2][C;!H0:3][S:4](=[O:5])(=[O:6])[C:7]>>[H:8][O:1][N:2]=[C:3][S:4](=[O:5])(=[O:6])[C:7]

217 [O:1]=[N:2][O:3].[C;H3:4][S:5](=[O:6])(=[O:7])[C:8]>>[O:3][N:2]([O:1][H:9])[C;H2:4][S:5](=[O:6])(=[O:7])[C:8]

218 [O;H1:3][N:2]([O;H1:1])[C:4][S:5](=[O:6])(=[O:7])[C:8]>>[O:3]=[N:2][C:4][S:5](=[O:6])(=[O:7])[C:8].[H:9][O:1][H:10]

219 [O:3][N:2]([O;H1:1])[C;!H0:4][S:5](=[O:6])(=[O:7])[C:8]>>[O:3][N:2]=[C:4][S:5](=[O:6])(=[O:7])[C:8].[H:9][O:1][H:10]

220 [O:1]=[N:2][O;H1:3].[C;!H0:4][N:5][O:6]>>[O:1]=[N:2][C:4][N:5][O:6].[H:7][O:3][H:8]

221 [O:1]=[N:2][C;!H0:3][N:4][O:5]>>[H:6][O:1][N:2]=[C:3][N:4][O:5]

222 [O:1]=[N:2][O:3].[C;H3:4][N:5][O:6]>>[O:3][N:2]([O:1][H:7])[C;H2:4][N:5][O:6]

223 [O;H1:3][N:2]([O;H1:1])[C:4][N:5][O:6]>>[O:3]=[N:2][C:4][N:5][O:6].[H:7][O:1][H:8]

224 [O:3][N:2]([O;H1:1])[C;!H0:4][N:5][O:6]>>[O:3][N:2]=[C:4][N:5][O:6].[H:7][O:1][H:8]

225 [O:1]=[N:2][O;H1:3].[N;!H0:4]>>[O:1]=[N:2][N:4].[H:5][O:3][H:6]

226 [O:1]=[N:2][N;!H0:3]>>[H:4][O:1][N:2]=[N:3]

227 [O:1]=[N:2][O:3].[N;!H0:4]>>[O:3][N:2]([O:1][H:5])[N:4]

228 [O;H1:3][N:2]([O;H1:1])[N:4]>>[O:3]=[N:2][N:4].[H:5][O:1][H:6]

229 [O:3][N:2]([O;H1:1])[N;!H0:4]>>[O:3][N:2]=[N:4].[H:5][O:1][H:6]

230 [O:1]=[N:2][O;H1:3].[N;!H0:4][c:5]1[c:6][c:7][c:8][c:9][c:10]1>>[O:1]=[N:2][N:4][c:5]1[c:6][c:7][c:8][c:9][c:10]1.[H:11][O:3][H:12]

231 [O:1]=[N:2][N;H1:3][c:4]1[c:5][c:6][c:7][c:8][c:9]1>>[H:10][O:1][N:2]=[N:3][c:4]1[c:5][c:6][c:7][c:8][c:9]1

232 [O:1]=[N:2][O:3].[N;!H0:4][c:5]1[c:6][c:7][c:8][c:9][c:10]1>>[O:3][N:2]([O:1][H:11])[N:4][c:5]1[c:6][c:7][c:8][c:9][c:10]1

233 [O;H1:3][N:2]([O;H1:1])[N:4][c:5]1[c:6][c:7][c:8][c:9][c:10]1>>[O:3]=[N:2][N:4][c:5]1[c:6][c:7][c:8][c:9][c:10]1.[H:11][O:1][H:12]

234 [O:3][N:2]([O;H1:1])[N;H1:4][c:5]1[c:6][c:7][c:8][c:9][c:10]1>>[O:3][N:2]=[N:4][c:5]1[c:6][c:7][c:8][c:9][c:10]1.[H:11][O:1][H:12]

235 [O:1]=[N:2][O;H1:3].[C;!H0:4][C:5]#[N:6]>>[O:1]=[N:2][C:4][C:5]#[N:6].[H:7][O:3][H:8]

236 [O;H1:3][N:2]([O;H1:1])[C:4][C:5]#[N:6]>>[O:3]=[N:2][C:4][C:5]#[N:6].[H:7][O:1][H:8]

237	[O:3][N:2]([O;H1:1])[C;!H0:4][C:5]#[N:6]>>[O:3][N:2]=[C:4][C:5]#[N:6].[H:7][O:1][H:8]
238	[C;!H0:3][N:2]([O;H1:1])[C:4][C:5](=[O:6])[O:7][c:8]1[c:9][c:10][c:11][c:12][c:13]1>>[C:3]=[N:2][C:4][C:5](=[O:6])[O:7][c:8]1[c:9][c:10][c:11][c:12][c:13]1.[H:14][O:1][H:15]
239	[C:3][N:2]([O;H1:1])[C;!H0:4][C:5](=[O:6])[O:7][c:8]1[c:9][c:10][c:11][c:12][c:13]1>>[C:3][N:2]=[C:4][C:5](=[O:6])[O:7][c:8]1[c:9][c:10][c:11][c:12][c:13]1.[H:14][O:1][H:15]
240	[C;!H0:3][N:2]([O;H1:1])[C:4][C:5](=[O:6])>>[C:3]=[N:2][C:4][C:5](=[O:6]).[H:7][O:1][H:8]
241	[C:3][N:2]([O;H1:1])[C;!H0:4][C:5](=[O:6])>>[C:3][N:2]=[C:4][C:5](=[O:6]).[H:7][O:1][H:8]
242	[O:1]=[N:2][C:3].[C;H3:4][S:5](=[O:6])[c:7]1[c:8][c:9][c:10][c:11][c:12]1>>[C:3][N:2]([O:1][H:13])[C;H2:4][S:5](=[O:6])[c:7]1[c:8][c:9][c:10][c:11][c:12]1
243	[C;!H0:3][N:2]([O;H1:1])[C:4][S:5](=[O:6])[c:7]1[c:8][c:9][c:10][c:11][c:12]1>>[C:3]=[N:2][C:4][S:5](=[O:6])[c:7]1[c:8][c:9][c:10][c:11][c:12]1.[H:13][O:1][H:14]
244	[C:3][N:2]([O;H1:1])[C;!H0:4][S:5](=[O:6])[c:7]1[c:8][c:9][c:10][c:11][c:12]1>>[C:3][N:2]=[C:4][S:5](=[O:6])[c:7]1[c:8][c:9][c:10][c:11][c:12]1.[H:13][O:1][H:14]
245	[O:1]=[N:2][C:3].[C;H3:4][S:5](=[O:6])(=[O:7])[C:8]>>[C:3][N:2]([O:1][H:9])[C;H2:4][S:5](=[O:6])(=[O:7])[C:8]
246	[C;!H0:3][N:2]([O;H1:1])[C:4][S:5](=[O:6])(=[O:7])[C:8]>>[C:3]=[N:2][C:4][S:5](=[O:6])(=[O:7])[C:8].[H:9][O:1][H:10]
247	[C:3][N:2]([O;H1:1])[C;!H0:4][S:5](=[O:6])(=[O:7])[C:8]>>[C:3][N:2]=[C:4][S:5](=[O:6])(=[O:7])[C:8].[H:9][O:1][H:10]
248	[C;!H0:3][N:2]([O;H1:1])[N:4][c:5]1[c:6][c:7][c:8][c:9][c:10]1>>[C:3]=[N:2][N:4][c:5]1[c:6][c:7][c:8][c:9][c:10]1.[H:11][O:1][H:12]
249	[C:3][N:2]([O;H1:1])[N;H1:4][c:5]1[c:6][c:7][c:8][c:9][c:10]1>>[C:3][N:2]=[N:4][c:5]1[c:6][c:7][c:8][c:9][c:10]1.[H:11][O:1][H:12]
250	[C;!H0:3][N:2]([O;H1:1])[C:4][C:5]#[N:6]>>[C:3]=[N:2][C:4][C:5]#[N:6].[H:7][O:1][H:8]
251	[C:3][N:2]([O;H1:1])[C;!H0:4][C:5]#[N:6]>>[C:3][N:2]=[C:4][C:5]#[N:6].[H:7][O:1][H:8]
252	[c:13]2[c:14][c:15][c:16][c:17][c:18]2[N:2]([O;H1:1])[C;!H0:3][C:4](=[O:5])[O:6][c:7]1[c:8][c:9][c:10][c:11][c:12]1>>[c:18]2[c:17][c:16][c:15][c:14][c:13]2[N:2]=[C:3][C:4](=[O:5])[O:6][c:7]1[c:8][c:9][c:10][c:11][c:12]1.[H:19][O:1][H:20]
253	[c:11]1[c:10][c:9][c:8][c:7][c:3]1[N:2]([O;H1:1])[C;!H0:4][C:5](=[O:6])>>[c:11]1[c:10][c:9][c:8][c:7][c:3]1[N:2]=[C:4][C:5](=[O:6]).[H:12][O:1][H:13]
254	[c:13]1[c:12][c:11][c:10][c:9][c:3]1[N:2]([O;H1:1])[C;!H0:4][S:5](=[O:6])(=[O:7])[C:8]>>[c:13]1[c:12][c:11][c:10][c:9][c:3]1[N:2]=[C:4][S:5](=[O:6])(=[O:7])[C:8].[H:14][O:1][H:15]
255	[c:11]1[c:10][c:9][c:8][c:7][c:3]1[N:2]([O;H1:1])[C;!H0:4][C:5]#[N:6]>>[c:11]1[c:10][c:9][c:8][c:7][c:3]1[N:2]=[C:4][C:5]#[N:6].[H:12][O:1][H:13]

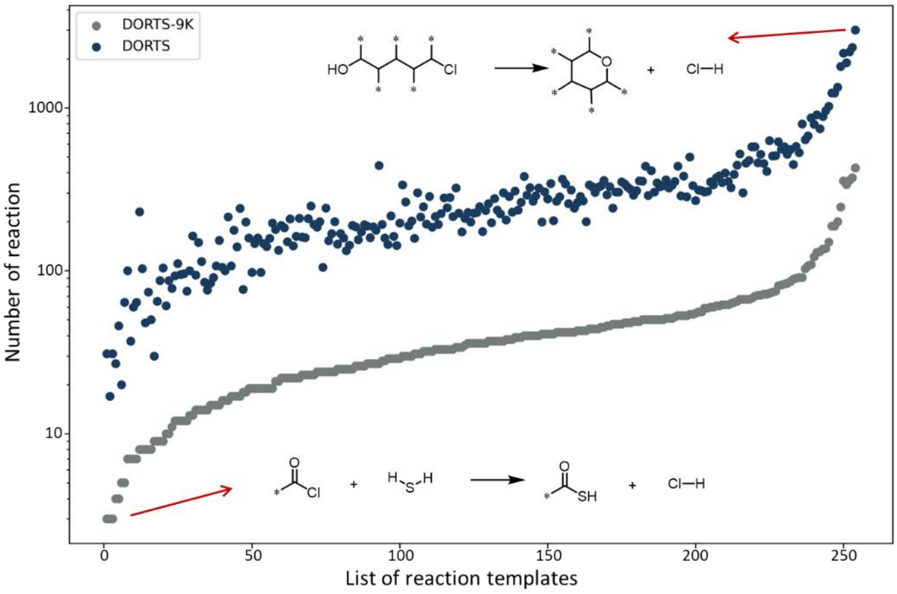

Suppl. Fig. 1 Distribution of reaction counts for DORTS-9K and DORTS across 255 reaction templates (the scatter plot illustrates the number of generated reactions per template, arranged in ascending order based on the DORTS database; grey dots denote the reaction counts in the DORTS-9K training subset; dark blue dots represent the total reaction counts in the comprehensive DORTS database; representative chemical transformations, such as substitution and addition reactions, are structurally depicted alongside their corresponding data points (indicated by red arrows)). Source data are provided as a Source Data file.

Suppl. Table 2 Reference energy baselines for individual atomic species (the tabulated values represent the reference energies used for data normalization in the DORTS-9K database, determined via DFT calculations at the ωB97M-V/def2-TZVP level).

Atom type	Reference energy
H	-16.207135472936834
C	-1036.9911886335535
N	-1489.0235184524395
O	-2046.731821396091
P	-9291.375536011736
S	-10834.466126460631
F	-2716.441171982367
Cl	-12522.258447185908
Br	-70041.934317188
I	-8101.434000183483

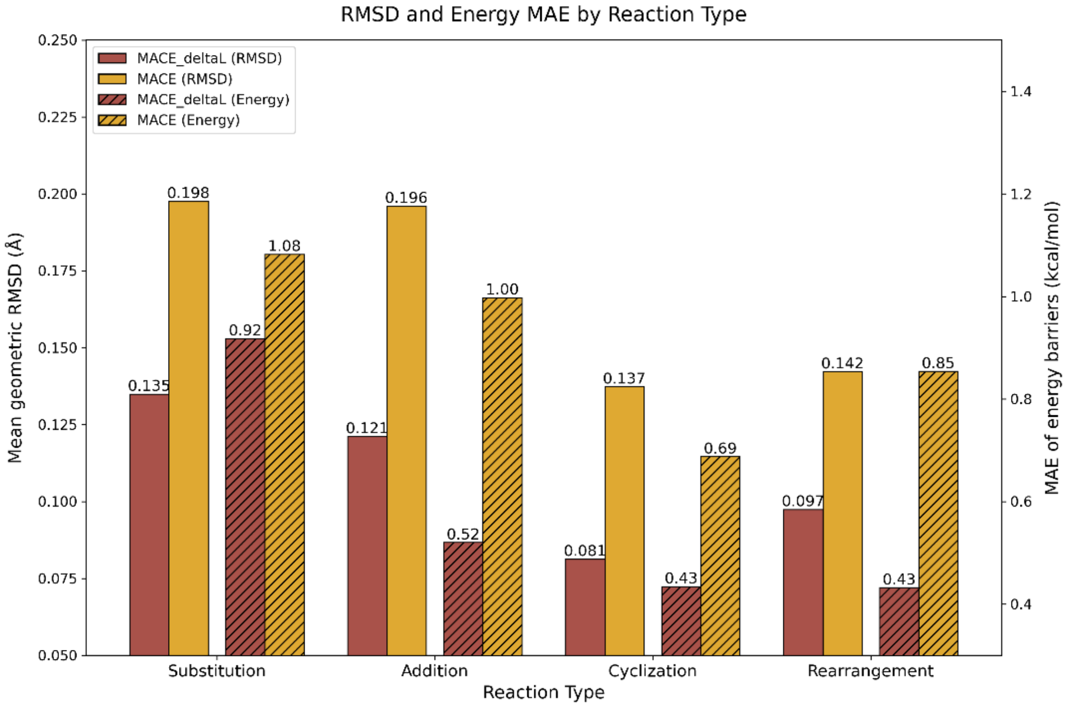

Suppl. Fig. 2 Error comparison of MACE and MACE_deltaL across four distinct organic reaction classes (the bar chart presents the prediction errors on the DORTS-1K test set categorized by substitution, addition, cyclization (ring-opening/closing), and rearrangement reactions; solid bars represent the mean geometric Root Mean Square Deviation (RMSD) of transition states (left *y*-axis), while hatched bars indicate the Mean Absolute Error (MAE) of the predicted reaction energy barriers (right *y*-axis); brown and gold colors denote the performance of the MACE_deltaL and pure MACE models, respectively). Source data are provided as a Source Data file.

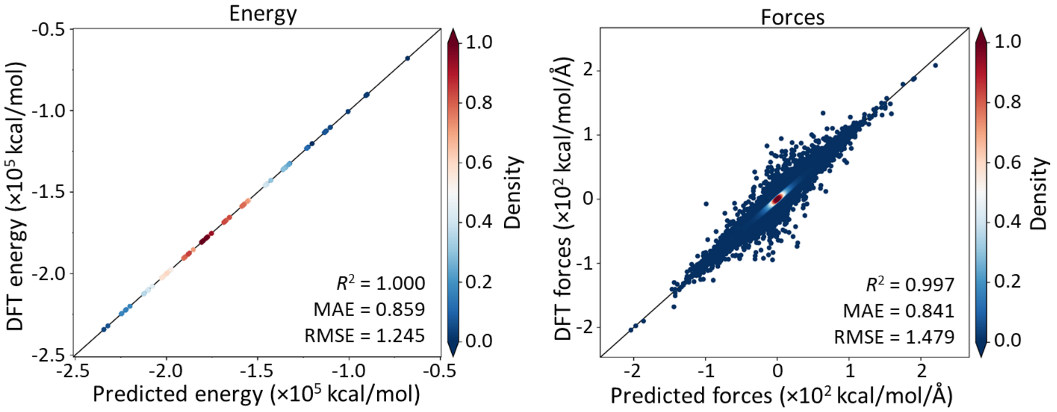

Suppl. Fig. 3 Performance of DeePEST-OS-T1x in cross-dataset validation. Source data are provided as a Source Data file.

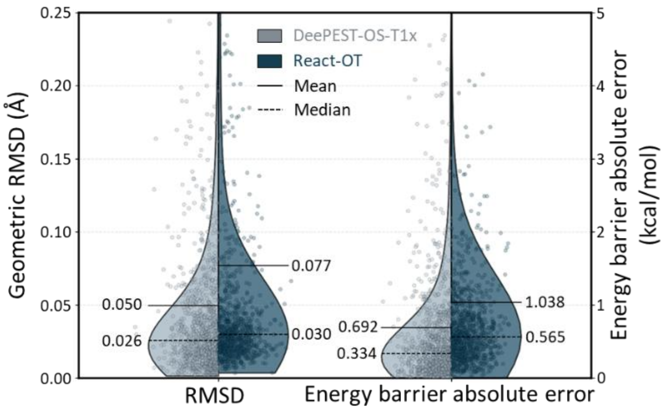

Suppl. Fig. 4 Accuracy evaluation based on external validation of 1,073 reactions (violin plots compare the transition state prediction accuracy between DeePEST-OS-T1x (light blue areas) and React-OT (dark blue areas); within each violin plot, horizontal solid black lines indicate the mean values, while dashed black lines represent the median values). Source data are provided as a Source Data file.

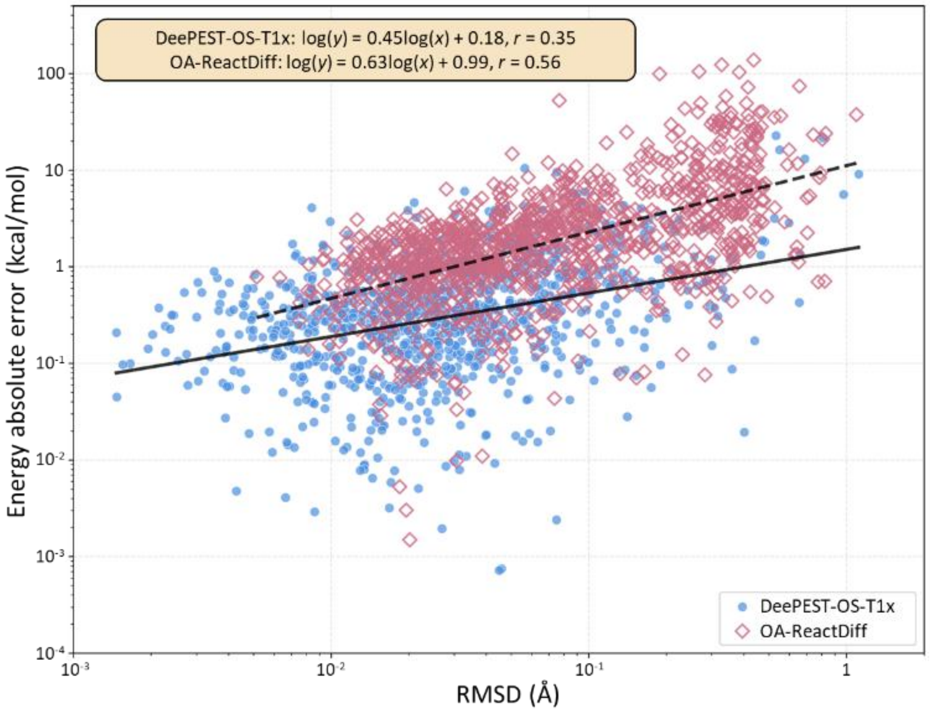

Suppl. Fig. 5 Relationship between transition state geometric RMSDs and energy absolute errors (scatter plot shows transition state energy absolute errors versus geometric RMSDs on a log-log scale for the 1,073 reactions in the Transition1x test set; blue filled circles and pink open diamonds represent predictions from DeePEST-OS-T1x and OA-ReactDiff, respectively; solid black line and dashed black line represent the corresponding linear regression fits for DeePEST-OS-T1x and OA-ReactDiff; the regression equations and Pearson correlation coefficients (r) are displayed in the inset box). Source data are provided as a Source Data file.

Suppl. Table 3 Hyperparameters used for training the DeePEST-OS model. The model based on the MACE architecture was trained with the specified parameters, resulting in a total of 8.23 million trainable parameters.

Parameter	Value
E0s	average
forces_weight	10
energy_weight	10
num_interactions	2
num_channels	256
max_L	2
correlation	3
r_max	6
batch_size	40
valid_batch_size	40
eval_interval	2
lr	1e-3
optimizer	adamw
swa	yes
start_swa	100
ema	yes
ema_decay	0.99
patience	10
seed	42
max_num_epochs	400
