# Supplementary material for: Reactive machine learning potential for accelerating transition state search in organic synthesis
Source: Nat Commun. 2026 May 8;17:6253. doi: 10.1038/s41467-026-72945-0 (PMC13376211; doi:10.1038/s41467-026-72945-0)
Supplement: Supplementary file 2 — Transparent Peer Review file [file 41467_2026_72945_MOESM2_ESM.pdf]

nature portfolio

Peer Review File

Reactive Machine Learning Potential for Accelerating Transition State Search in Organic Synthesis

Corresponding Author: Dr Qilei Liu

This file contains all reviewer reports in order by version, followed by all author rebuttals in order by version.

Version 0:

Reviewer comments:

Reviewer #1

(Remarks to the Author)
The authors present DeePEST-OS, a delta-learning MLIP framework for TS application. The novelty of this work lies in two folds. From methodology perspective, it applies delta-learning on 3D TS optimization to correct xTB level of theory. From the dataset perspective, it push the elements out of CNOH for 3D reactive dataset to additionally have P, S, F, Cl, Br, I. Latter is much needed in the field and I am happy that someone starts to push towards this direction. However, I have some major concerns before the manuscript can be published.

Major comments.
1. One potential drawback is the explicit coupling to semi-empirical methods such as xTB. This would influence its performance on meta-containing systems where xTB are bad at and on large systems as xTB has N^3 scaling in computational cost. This is just the feature of this delta-learning approach, which I would like the author to discuss these limitation more in the Discussion section

2.The authors seem to get much lower barrier height errors at a similar structure RMSD (see Table 1 at the NCS paper https://doi.org/10.1038/s43588-023-00563-7). I am quite curious why that is. It would be great to have a scatter plot for RMSD vs. barrier height error to look into this observation.

3.React-OT requires matched RP conformation pairs as input, which is not performed in this work. Therefore, I doubt the comparison in Fig 6b is not fair. In addition, some structures from DFT, DeePEST-OS, and React-OT are very similar by the eye. I am wondering why their energy differs so much?

4. The comparison on time cost is not rigorous. Th author states that there is a 93.9 core·s cost from post-processing DFT calculations. But this post-processing step in required in any methods if they aim for DFT level accuracy in the end. In some work thess DFT calculations are performed explicitly, but its does not mean that this step cannot be replaced by ML, if the goal is simply to make the entire workflow run faster. Nowadays, every code can be run on either cpu or gpu without too much barrier. DFT easily be run on GPU4PySCF, for example. In addition, the time cost depends a lot on engineering efforts. Anyways, I would like the authors to reduce apple-to-banana comparison that would affect the quality of this originally great work.

Minor comments
1. There is a sentence in the introduction saying “More critically, the React-OT model generates … predict reaction kinetic properties”. This is not accurate. Zhao et al. recently tested React-OT with IRC verification and found that React-OT achieves better TSOpt convergence and IRC intended reaction rate compared to MLIP approaches (Adv Sci paper https://advanced.onlinelibrary.wiley.com/doi/full/10.1002/advs.202506240 and preprint at https://arxiv.org/abs/2505.12447). There might be any references that I missed, which I encourage the authors to look deeper into the direction to make sure their statements are accurate.

2.MACE is known to be slow. I am wondering the number of parameters of the MACE model that the author used and the number of step of optimizations.

(Remarks on code availability)
I do not have time to review the code in this round. If the manuscript is much improved during the revision, I will have the time to review the code in the next round of review.

Reviewer #2

(Remarks to the Author)
I co-reviewed this manuscript with one of the reviewers who provided the listed reports. This is part of the Nature Communications initiative to facilitate training in peer review and to provide appropriate recognition for Early Career Researchers who co-review manuscripts.

(Remarks on code availability)

Reviewer #3

(Remarks to the Author)
The paper presents a machine-learning (ML) model targeted at predicting reaction barriers for organic chemical reactions. The ML model generated is based on the widely-used MACE architecture, using a “delta-ML” approach to boost accuracy. Compared to previous ML models, the approach described here seeks to expand the range of element types that can be treated. Overall, the structure of the paper is appropriate to the topic - comments about the writing and figures are given below. The conclusions presented are supported by the available data given.

A first major comment is that the authors should be clearer about the novelty of their approach relative other other ML models, and should also place the predictive capability of their model in the context of actual experiments. The authors have generated a large dataset of organic reactions using textbook organic chemical transformations, then fit a MACE model to the DFT data for these reactions. This fitting works well - one should probably expect so, given that it’s a “delta-ML” model based on a decent baseline. The MACE model is already widely used, and previous work has already demonstrated use of MACE to calculate reaction barriers (J. Chem. Phys. 162, 114122 (2025)). So - being clearer about the novelty of this particular ML strategy is important and requires more discussion. More broadly, the research presented here does nothing to address the problem with ML models that is almost-always overlooked - you’re training against DFT data that will have errors relative to experiment, so how do you know that your ML model is actually any good in making “real world” predictions?

A second key comment: the writing in the paper has a number issues to correct, and is prone to hyperbole (which can very distracting throughout). For example, as noted below, there are some factually incorrect statements, including in the abstract. The authors should make sure to address these throughout.

More detailed comments are as follows:

(1) In Abstract - DFT is not a “method for transition-state searches”. It is a method for calculating energy.

(2) In Abstract, the authors note that DFT has a trade-off between accuracy and cost. A ML model does nothing to address accuracy - but this is a stated target of the author’s work (as noted in the first sentence of the abstract).

(3) A ML potential cannot be both “generic” and yet specifically cover 10 element types - reword.

(4) The level of hyperbole and over-emphasis in the paper is generally distracting - examples include “remarkable accuracy”, “significantly advancing the acceleration of QC calculations”, “expansive realm”, “remarkable diversity”, “As a classic textbook and authoritative reference book in the field of organic chemistry, March’s Advanced Organic Chemistry…”, “… deeply coupling the mechanism driven semi-empirical…”, “This breakthrough in chemical intelligence underscores…”, “This advancement elevates organic reaction kinetics prediction to a new synergistic paradigm…”. These sorts of phrases are not necessary and should be toned down.

(5) Line 33 - “with essential applications in fields.” is a vague sentence that should be replaced.

(6) Line 33/34 - How does reaction kinetics help “control the rates of chemical reactions”? Reword.

(7) Semi-empirical methods like XTB and DFTB can behave extremely poorly for reactions - for example, predicting multiple minima along a reaction path or even predicting transition-states that don’t lead to the expected products/reactants. So, it’s not just the barriers and geometries that can be untrustworthy, but the entire character of the reactions under study. How do the authors address this in their work?

(8) The cartoon characters in Figure 1 are not appropriate and should be removed.

(9) Line 138 - by limiting the generated database to only those previously-known reactions extracted from a textbook, does this limit the scope of the trained model so that it cannot identify truly novel chemistry? Also, the reaction templates/types should be listed somewhere - they don’t seem to be included.

(10) From the description of methodology, it sounds like transition-state geometries are only determined using XTB? Or are they optimised again using DFT?

(11) Line 246 - the relative times of generating datasets with XTB and DFT don't "…highlight the efficiency of the proposed strategy." - they simply reflect that fact that XTB is faster than DFT. Reword.

(12) Line 267 - it would be useful to know where the improvements over the "basic" MACE (without delta-ML) come from? Are there specific reaction types that can be identified that are treated more accurately in the Deepest-OS model? Some further analysis would be interesting.

(13) Line 294 - "which is widely considered one of the best open-source transition state search algorithms currently available." - these sorts of subjective statements should be removed.

(14) Line 298 - "Particularly in IRC path calculations, MACE-deltaL (0.11 core·s·step-1) achieves an efficiency breakthrough of four
orders of magnitude compared to DFT (2,577.2 core·s·step-1) (Fig. 4(d))". Again, the hyperbole here should be removed - in addition, it is worth noting that these time comparisons are not directly a result of the work reported here - MACE and XTB are just faster than DFT, so it's not clear why the authors are emphasising this point.

(15) Line 321 - are the 880 transition-states re-optimized using MACE, Painn, etc.? If not, this comparison does not seem fair, as it's not really comparing TS structures for each model.

(16) Line 335 - "marking a fitting accuracy better than ±1 kcal/mol for transition state energies.". It is important that the authors discuss the fact that this fitting accuracy is only relative to DFT data - it does NOT reflect absolute accuracy relative to experiment. Can the authors say something about how they expect the barrier accuracy to compare to experimental data? Has this been assessed for their ML model?

(17) Line 368 - If comparing timings, please use the same computing resources.

(18) Line 375 - "cannot be accelerated by GPU due to the inherent limitations of DFT." - I believe this is incorrect. There are GPU-accelerated DFT implementations. This should be either removed or updated.

(19) Line 401 - most stable conformer? (not "modest stable")

(20) A general comment is that the transferability of the trained ML models here is not strongly discussed or demonstrated. For example, in Line 345 onwards, reactions from transition1x are studied - but how similar are these to the original deepest-os training set? What if deepest-OS is applied to systems that are dissimilar to the training set? Is it possible to better quantify the transferability here, rather than just looking at a single additional example (in the final section about retrosynthesis)?

(21) Again, Figure 7 only represents a comparison of the ML model against DFT. What about comparison to experimentally-determined barriers?

(22) Line 174, Figure 2b: If the y-axis of this figure is intended to be energy and the x-axis is the reaction coordinate, it is unclear what the two paths passing through F_max_rev and F_max_forw are, and why they each start/end at the energy of the reactants/products but the reaction coordinate position of the TS. Also, while I understand that these names correspond to the two conformations with maximum force, they should be made clearer for less technically inclined audiences.

(23) Line 188: The authors say that the reactions in DORTS-9K were sampled randomly from the wider DORTS dataset, rather than being stratified by reaction classes to ensure there is no bias towards/against any particular reaction types in DORTS-9K. Given the authors already have reaction class labels from the generation of DORTS, do they anticipate that random sampling could leave out or underemphasise any reaction classes when constructing DORTS-9K? To test this, they could check for a uniform distribution of the 294 reaction templates in the reactions that made it into DORTS-9K.

Furthermore, given that the full DORTS dataset already contains 7.5 million conformations from across the whole range of input reactions, why did the authors not sample (with stratification by reaction class) these conformers directly? Surely this would yield a more chemically diverse dataset than DORTS-9K, given that conformers from all 74,837 reactions are present rather than only containing conformers from the 9,017 reactions selected in DORTS-9K?

(24) Line 215, Figure 3b: It appears that many of the transition states from the Transition1x dataset fall outside of the latent chemical space that DORTS encompasses, evidenced by a lack of points around many of the red regions. Does this indicate that models trained on DORTS-9K would fail to accurately predict the reaction paths of the reactions from Transition1x, or is it an artefact of how t-SNE was run on this data? Either way, this should be clarified in the text.

(25) Line 215, Figure 3c: The latent chemical space shown in this figure is not the same as in Figure 3b, so the two are not directly comparable. I assume this is because a different t-SNE map was constructed without the reactions from Transition1x? However, this means the two figures cannot be compared. Instead, the same t-SNE map could be used for both figures, but with the Transition1x points excluded from Figure 3c.

(26) Line 385: The authors state that test cases for the DeePEST-OS/React-OT benchmark are detailed in the supporting information, but this is not present.

(27) While it is clear that DeePEST-OS excels at prediction of reaction energetics, when performing chemical kinetics simulations this is often only half of the problem – additionally, the thermodynamics of reactions must be considered. In conventional transition state theory, this requires accurate vibrational energies of both reactants and transition states. While I appreciate that benchmarking this would be a significant undertaking, it would be valuable and highly relevant to the discussion of the simulation of chemical reaction networks.

(28) The additional dataset zip file that is being provided with the paper has no documentation and is not mentioned in the text. It appears to contain XYZ geometries for Table 2, Figure 6 and Figure 7. This is useful and should be included, it just needs mentioning in e.g. the Data Availability section.

(Remarks on code availability)
I haven't cloned the code and tried to run it, but I have looked at the GitHub repo given and it looks like it is well presented and well documented. For example, there are detailed installation instructions, examples, and so on. Further comments about data availability are in the main review.

Version 1:

Reviewer comments:

Reviewer #1

(Remarks to the Author)
The authors have done substantial work in this revision for the comments from other reviewers and myself. All my comments are addressed. I really like their efforts on pushing datasets towards elements other than CNOH, which is much needed in the field towards real impactful applications.

(Remarks on code availability)
The repo is complete with proper README and model checkpoints. But I have to be transparent and say that I did not spare time to make a full run of reproduction.

Reviewer #2

(Remarks to the Author)
I co-reviewed this manuscript with one of the reviewers who provided the listed reports. This is part of the Nature Communications initiative to facilitate training in peer review and to provide appropriate recognition for Early Career Researchers who co-review manuscripts.

(Remarks on code availability)

Reviewer #3

(Remarks to the Author)
The authors have done a good job in addressing my previous comments. I do not have any further major comments to add. The work should be a useful addition to the growing number of ML models for predicting reaction properties such as activation energies.

The further analysis of sampled chemical reaction space is very useful, as is the further performance and transferability analysis performed for alternative datasets. The new sections and additions help set the context for the work, and provide further insight into the range of applicability of the ML model developed here.

A minor comment is that some of the figures are missing labels on their axes - for example, the new Figure 3 is missing axis labels.

While I have not fully cloned and tested the uploaded software, I have had a look at the GitHub repository and it appears to contains the relevant information and documentation that one would expect (at least on the front page). A further comment here is that some sort of permanent DOI (or deposit somewhere with this facility) would help ensure that this software and data remains available in the longer term - as it is, this software and data appears to be associated with one of the authors personal GitHub repository.

(Remarks on code availability)
See in my main comments.

Open Access This Peer Review File is licensed under a Creative Commons Attribution 4.0 International License, which permits use, sharing, adaptation, distribution and reproduction in any medium or format, as long as you give appropriate credit to the original author(s) and the source, provide a link to the Creative Commons license, and indicate if changes were made.
In cases where reviewers are anonymous, credit should be given to 'Anonymous Referee' and the source.
The images or other third party material in this Peer Review File are included in the article's Creative Commons license, unless indicated otherwise in a credit line to the material. If material is not included in the article's Creative Commons license and your intended use is not permitted by statutory regulation or exceeds the permitted use, you will need to obtain permission directly from the copyright holder.
To view a copy of this license, visit https://creativecommons.org/licenses/by/4.0/

Response to the Reviewers

Dear Editor,

Thank you so much for your letter and the reviewers' comments on our manuscript submitted to *Nature Communications* (Submission title: Reactive Machine Learning Potential for Accelerating Transition State Search in Organic Synthesis; Manuscript ID: NCOMMS-25-69131).

After carefully studying the reviewers' comments, we have made a thorough revision to our manuscript and all the comments have been addressed. The revised manuscripts (clean and marked versions), with the updated Figures, Tables and supporting information (clean and marked versions), have been submitted. We would like to thank you and all the reviewers for their constructive comments and help with this manuscript. In the text below, we are providing a detailed list of changes made to address the reviewers' comments.

Note that reviewers' comments are given in *italics*. Authors' responses to reflect the changes made are indicated as regular text. All changes are marked in yellow in the revised (marked) manuscript and supporting information.

If you have any question about this manuscript, please don't hesitate to let me know. Thank you and best regards!

Yours sincerely,

Qilei Liu

Address: State Key Laboratory of Fine Chemicals, Frontiers Science Center for Smart Materials Oriented Chemical Engineering, Department of Pharmaceutical Sciences, Institute of Chemical Process Systems Engineering, School of Chemical Engineering, Dalian University of Technology, Dalian 116024, China

Email: liuqilei@dlut.edu.cn

Reviewer 1:

Comments to the Author:

The authors present DeePEST-OS, a delta-learning MLIP framework for TS application. The novelty of this work lies in two folds. From methodology perspective, it applies delta-learning on 3D TS optimization to correct xTB level of theory. From the dataset perspective, it push the elements out of CNOH for 3D reactive dataset to additionally have P, S, F, Cl, Br, I. Latter is much needed in the field and I am happy that someone starts to push towards this direction. However, I have some major concerns before the manuscript can be published.

We greatly appreciate your meticulous reading of our manuscript and your insightful comments. Your positive recognition of our work is a great encouragement to us. In the following we respond to each of the comments.

Major comments：

1. One potential drawback is the explicit coupling to semi-empirical methods such as xTB. This would influence its performance on meta-containing systems where xTB are bad at and on large systems as xTB has N^3 scaling in computational cost. This is just the feature of this delta-learning approach, which I would like the author to discuss these limitation more in the Discussion section.

Clarification:

We greatly appreciate your comments and fully agree with your concerns regarding the limitations of the GFN2-xTB method.

To enrich the Discussion section with additional insights into the computational efficiency of GFN2-xTB for large systems, we have conducted supplementary studies to evaluate the inference times of GFN2-xTB and MACE_deltaL on DORTS-1K (1,000 novel reactions not included in DORTS-9K) for transition state geometry optimizations. **Fig. 5(d)** presents a comparison of these inference times across reaction systems with varying numbers of atoms. Both methods exhibit an increase in computation time as the number of atoms in the reaction system grew, among which GFN2-xTB demonstrates the faster speed, while MACE_deltaL shows the slower performance due to its coupling of GFN2-xTB and MACE. As shown in **Fig. 5(d)**, the computational cost of GFN2-xTB scales approximately as O(N^3) with respect to the number of atoms,

which aligns with the concerns you raised and the results reported in the literature [arXiv:2505.09606]. Although GFN2-xTB exhibits $O(N^3)$ scaling that limits its efficiency for large systems, the reaction systems studied in this work are not extremely large (typically containing 6 to 50 atoms), thus its computational efficiency remains acceptable. The average inference time of MACE_deltaL (specifically 0.084 core·s·step^{-1}) was approximately 2.53 times that of GFN2-xTB (0.033 core·s·step^{-1}), yet remained below 0.1 core·s·step^{-1}. Therefore, the developed MACE_deltaL model still satisfies the requirements of high-throughput transition state geometry optimizations for the currently studied system sizes. However, extending its application to significantly larger systems in the future will raise the computational-efficiency concern you mentioned, which we have discussed in the Discussion section of the revised manuscript.

Another point worthy of note is that MACE_deltaL employs Δ-learning coupled with the GFN2-xTB semi-empirical method for both training and inference. Therefore, its applicability is inherently constrained by the limitations of GFN2-xTB. Although GFN2-xTB exhibits relatively excellent performance in organic systems (the mean RMSD of transition state geometries optimized by GFN2-xTB in **Fig. 5(a)** is 0.31 Å), it may have accuracy limitations in metal-containing reaction systems. For future training of Δ-learning models for metal-containing systems, attempts can be made to couple the g-xTB method [Preprint at ChemRxiv https://doi.org/10.26434/chemrxiv-2025-bjxvt], which performs better in metal systems, or pre-trained machine learning potential models for metal-containing systems (e.g., mace-OMAT-0). We have discussed this limitation raised by the reviewer in the Discussion section of the revised manuscript.

Action taken:

- The paragraph "*As a semi-empirical method, GFN2-xTB exhibits a computational cost scaling approximately as $O(N^3)$ with respect to the number of atoms[54], leading to high computational demands for MACE_deltaL when applied to large molecular systems. To evaluate practical performance, the inference times of GFN2-xTB and MACE_deltaL for transition state geometry optimization were assessed on DORTS-1K.* ***Fig. 5(d)*** *compares the inference times across reactions with varying atom counts. Both methods demonstrate increased computation times with growing system size, with MACE_deltaL being slower than GFN2-xTB due to its coupling of GFN2-xTB and MACE. Although GFN2-xTB exhibits an approximate $O(N^3)$*

scaling that limits its efficiency for large systems, the reaction systems studied in this work were moderate in size (typically 6-50 atoms), thus its computational efficiency remained acceptable. The average inference time of MACE_deltaL (0.084 core·s·step^{-1}) was approximately 2.53-fold higher than that of GFN2-xTB (0.033 core·s·step^{-1}), yet remained below 0.1 core·s·step^{-1}. Consequently, MACE_deltaL maintains sufficient efficiency to satisfy the requirements of high-throughput transition state geometry optimizations for the currently studied system sizes. However, future extension to substantially larger systems will face significant computational-efficiency challenges." **has been added** (see **Lines 563-575** in the revised manuscript).

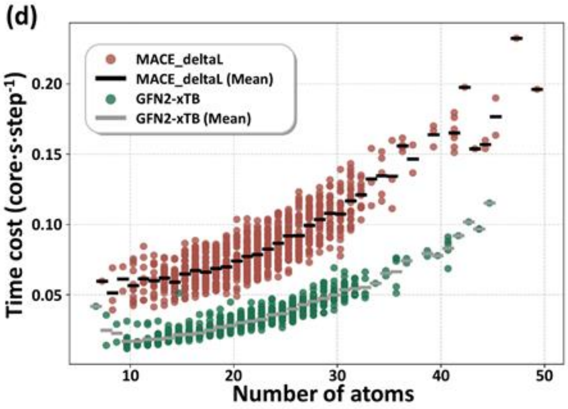

Fig. 5 Accuracy and computational cost evaluations based on the test set. (**d**) Comparison of computational costs for transition state geometry optimization across systems with different numbers of atoms for GFN2-xTB and MACE_deltaL based on DORTS-1K.

- The sentence "*Another point worthy of note is that MACE_deltaL employs Δ-learning coupled with the GFN2-xTB semi-empirical method for both training and inference. Therefore, its applicability is inherently constrained by the limitations of GFN2-xTB. Although GFN2-xTB exhibits relatively excellent performance in organic systems (the mean RMSD of transition state geometries optimized by GFN2-xTB in **Fig. 5(a)** is 0.31 Å), it may have accuracy limitations in metal-containing reaction systems. For future training of Δ-learning models for metal-containing systems, attempts can be made to couple the g-xTB method[64], which performs better in metal systems, or pre-trained MLP models for metal-containing systems[65].*" **has been added** (see **Lines 576-582** in the revised manuscript).

2. The authors seem to get much lower barrier height errors at a similar structure RMSD (see Table 1 at the NCS paper https://doi.org/10.1038/s43588-023-00563-7). I am quite curious why that is. It would be great to have a scatter plot for RMSD vs. barrier height error to look into this observation.

Clarification:

Thank you for your valuable and constructive comments. To enable a direct comparison between transition states optimized by the machine learning potential model (DeePEST-OS-T1x) and those generated by the end-to-end model (OA-ReactDiff), the same correlation analysis between transition state geometric RMSDs and energy absolute errors was conducted. For consistency with the OA-ReactDiff setting, the identical test split used for OA-ReactDiff evaluation was adopted, and a log-log linear fit was performed (**Fig. S5**). The regression equation obtained in this study is $\log(y) = 0.45\log(x) + 0.18$ with the Pearson correlation coefficient (r) value of 0.35, whereas the equation reported by Duan et al. is $\log(y) = 0.63\log(x) + 0.99$ with the r value of 0.56. On the one hand, both low r values indicate only a moderate-to-weak linear relationship between transition state geometric RMSDs and energy absolute errors. On the other hand, the smaller slope and intercept of DeePEST-OS-T1x indicate that, for a given transition state RMSD, its fitted relationship implies a lower energy absolute error compared to OA-ReactDiff. A plausible explanation is that DeePEST-OS-T1x is trained directly on energies and forces, which can reduce energy-related deviations, whereas OA-ReactDiff is primarily optimized for minimizing transition state geometric RMSDs. Similar observations have been reported by King et al [arXiv:2512.06592], who demonstrated that although models achieve high structural precision with low geometric RMSDs, sole optimization for this metric fails to ensure accurate energy predictions. This limitation arises from the sensitivity of energy to minor geometric deviations, highlighting the critical need to incorporate energy constraints during model training.

Action taken:

- The paragraph "*Furthermore, to investigate the relationship between energy absolute errors and geometric RMSDs of transition states, DeePEST-OS-T1x was compared with OA-ReactDiff—an end-to-end generative model for transition state geometry prediction developed by Duan et al. and trained on the Transition1x database*[55]*. To enable a consistent comparison, a similar correlation analysis was*

*performed using the same test split and a log-log linear fit (**Fig. S5**). The regression equation of DeePEST-OS-T1x is $log(y) = 0.45log\,(x) + 0.18$ with a Pearson correlation coefficient (r) value of 0.35, whereas the regression equation of OA-ReactDiff is $log(y) = 0.63log\,(x) + 0.99$ with r = 0.56. Both low r values indicate only a moderate-to-weak linear relationship between transition state geometric RMSDs and energy absolute errors. Notably, the smaller slope and intercept of DeePEST-OS-T1x indicate that, for a given transition state RMSD, its fitted relationship implies a lower energy absolute error compared to OA-ReactDiff. A plausible explanation is that DeePEST-OS-T1x is trained directly on energies and forces (mitigating energy-related discrepancies), whereas OA-ReactDiff is primarily optimized for minimizing transition state geometric RMSDs. This observation aligns with the findings by King et al.[56], who demonstrated that although models achieve high structural precision with low geometric RMSD, sole optimization for this metric fails to ensure accurate energy predictions. This limitation arises from the sensitivity of energy to minor geometric deviations, highlighting the critical need to incorporate energy constraints during model training.*" **has been added** (see **Lines 429-443** in the revised manuscript).

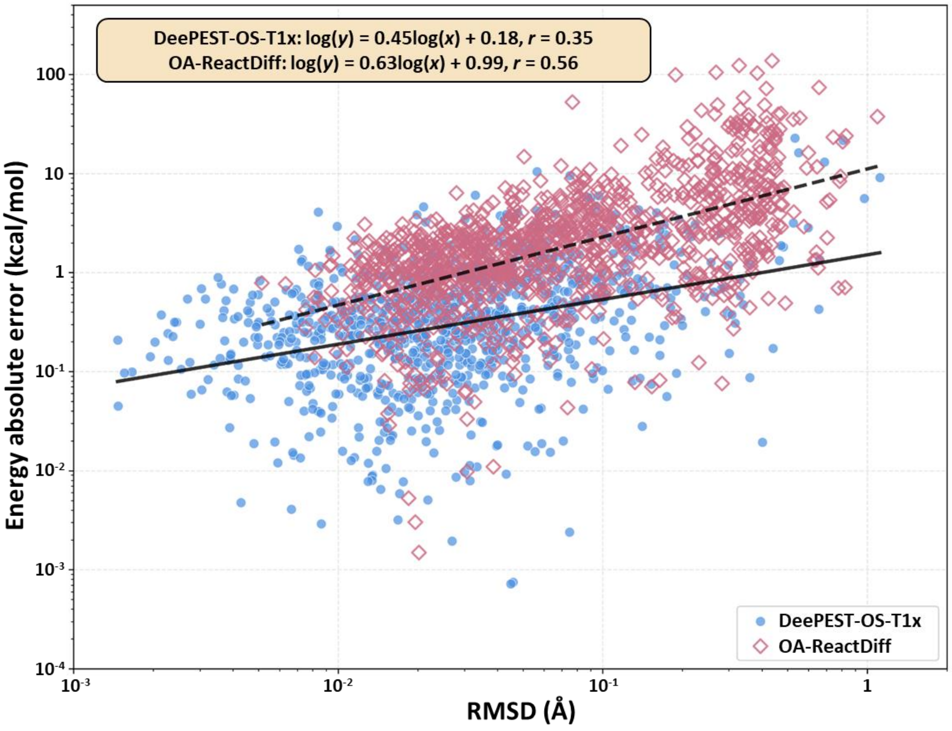

Fig. S5 Scatter plot of transition state energy absolute errors versus geometric RMSDs on a log-log scale, comparing DeePEST-OS-T1x (blue) and OA-ReactDiff (red) using data from the Transition1x test set (1,073 reactions).

3. React-OT requires matched RP conformation pairs as input, which is not performed in this work. Therefore, I doubt the comparison in Fig 6b is not fair. In addition, some structures from DFT, DeePEST-OS, and React-OT are very similar by the eye. I am wondering why their energy differs so much?

Clarification:

Thank you so much for this valuable suggestion.

React-OT requires both reactant and product structures to generate transition states, thereby classifying it as a double-ended transition state search algorithm. In contrast, the algorithm employed in this work to generate initial guesses of transition states (GENiniTS-RS) requires only a single input, either a reactant or a product structure, thus classifying it as a single-ended transition state search algorithm. As you rightly noted, this comparison in **Fig. 6(b)** is inherently unfair. Given that the primary goal of this section is to demonstrate that DeePEST-OS is able to identify the most stable transition state conformation when integrated with GENConf-TS—rather than to benchmark against other methods—we have therefore removed the comparative data for React-OT in **Fig. 6(b)**, along with all associated descriptions in the manuscript.

Additionally, visual similarity does not guarantee structural identity. As shown in **Fig. R1**, three transition state structures generated by DFT, DeePEST-OS, and React-OT, respectively, appear nearly identical. However, upon molecular alignment, the overlap between the React-OT and DFT transition state structures is significantly less than that between the DeePEST-OS and DFT structures. Further analysis using the root mean square deviation (RMSD) metric quantifies this difference, showing that the React-OT/DFT structural error (RMSD = 0.306 Å) is substantially greater than the DeePEST-OS/DFT error (RMSD = 0.024 Å). This demonstrates that even visually similar molecules can exhibit significant structural discrepancies, highlighting the necessity for quantitative measures like RMSD. The large React-OT/DFT structural error (0.306 Å) also accounts for the considerably larger ΔE_{TS} value compared to DeePEST-OS. Given that **Fig. 6(b)** has been removed, the discussion here has not been included in the revised manuscript and supporting information.

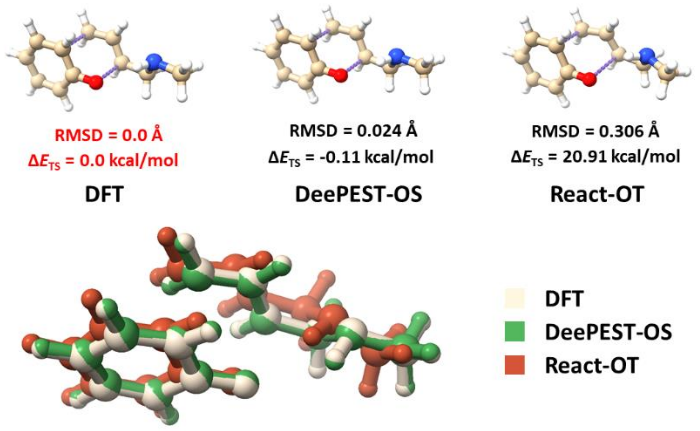

Fig. R1 Comparison of the transition state structures obtained by DFT, DeePEST-OS, and React-OT, and the corresponding energy errors calculated by DFT for these structures.

Action taken:

➢ The comparative data for React-OT in **Fig. 6(b)**, along with all associated descriptions in the manuscript, have been removed.

4. The comparison on time cost is not rigorous. Th author states that there is a 93.9 core·s cost from post-processing DFT calculations. But this post-processing step in required in any methods if they aim for DFT level accuracy in the end. In some work thess DFT calculations are performed explicitly, but its does not mean that this step cannot be replaced by ML, if the goal is simply to make the entire workflow run faster. Nowadays, every code can be run on either cpu or gpu without too much barrier. DFT easily be run on GPU4PySCF, for example. In addition, the time cost depends a lot on engineering efforts. Anyways, I would like the authors to reduce apple-to-banana comparison that would affect the quality of this originally great work.

Clarification:

Thank you so much for your valuable comments. We acknowledge that the time comparison for energy calculations in the original manuscript is not sufficiently rigorous, and as such, we have removed all related content from the revised version. In addition, to address comments from other reviewers, we have adjusted the inference processes of the React-OT and DeePEST-OS-T1x models to run on the same GPU. For React-OT, switching the GPU from an NVIDIA V100 to a GeForce RTX 4060 Laptop reduced the average transition state optimization time from 5.8 seconds to 1.8 seconds.

For DeePEST-OS-T1x, the initial inference time had included both transition state optimization and frequency calculation. To ensure a fair comparison, we have now excluded the frequency calculation step, which brought the average transition state optimization time down from 3.4 seconds to 1.4 seconds. Detailed calculation information is available on GitHub at https://github.com/kaipai-ren/DeePEST-OS/tree/main/dataset/ for your review.

Action taken:

- The sentence "*Among the 980 reactions that are successfully optimized by DeePEST-OS-T1x, React-OT, and DFT approaches (see **Tab. 2**), DeePEST-OS-T1x demonstrates significantly superior performance, exhibiting lower mean geometric RMSD (0.051 Å vs. 0.082 Å) and energy absolute error (0.690 kcal/mol vs. 0.999 kcal/mol) compared to React-OT (Violin plot see **Fig. S2**). Notably, DeePEST-OS-T1x achieves dramatically higher computational efficiency, requiring only 3.4 core·s per reaction on average with NVIDIA GeForce RTX 4060 Laptop GPU for transition state optimization without additional energy computation steps while maintaining DFT-level accuracy. In contrast, React-OT necessitates GPU acceleration for transition state optimization (5.8 s per reaction on average on NVIDIA V100) but incurs substantial post-processing overhead due to DFT energy calculations (93.9 core·s per reaction on average).*" **has been revised to** "*Among the 995 reactions for which transition states were successfully optimized using DeePEST-OS-T1x, React-OT, and DFT (**Tab. 2**), DeePEST-OS-T1x demonstrates superior accuracy over React-OT, with a lower mean geometric RMSD (0.050 Å vs. 0.077 Å) and a smaller MAE of reaction energy barriers (0.692 kcal/mol vs. 1.038 kcal/mol) (see the violin plots in **Fig. S4**). Notably, DeePEST-OS-T1x was also more efficient, requiring an average of only 1.4 s per reaction for transition state optimization on an NVIDIA GeForce RTX 4060 Laptop GPU, whereas React-OT required 1.8 s per reaction. In addition, a distinct advantage of DeePEST-OS-T1x is its ability to simultaneously output a near-DFT-level predicted energy during transition state optimization. In contrast, React-OT focuses solely on geometry and does not provide energy outputs during transition state generation.*" (see **Lines 416-424** in the revised manuscript).
- **Tab. 2 has been revised** (see **Line 426** in the revised manuscript).

Tab. 2 Comparison of transition state geometric RMSDs, reaction energy barrier absolute errors, and computational costs between DeePEST-OS-T1x and React-OT models on the Transition1x external test set.

**Model**	**Geometric RMSD (Å)**		**Energy barrier absolute error (kcal/mol)**		**Computational cost (s)**
	Mean	Median	Mean	Median	Transition state optimization
React-OT	0.077	0.030	1.038	0.565	1.8
**DeePEST-OS-T1x**	**0.050**	**0.026**	**0.692**	**0.334**	**1.4**

Minor comments:

1. There is a sentence in the introduction saying "More critically, the React-OT model generates ... predict reaction kinetic properties". This is not accurate. Zhao et al. recently tested React-OT with IRC verification and found that React-OT achieves better TSOpt convergence and IRC intended reaction rate compared to MLIP approaches (Adv Sci paper https://advanced.onlinelibrary.wiley.com/doi/full/10.1002/advs.202506240 and preprint at https://arxiv.org/abs/2505.12447). There might be any references that I missed, which I encourage the authors to look deeper into the direction to make sure their statements are accurate.

Clarification:

We deeply apologize for overlooking this reference and also for making inappropriate comments on the work of Zhao et al. [*Adv. Sci.*, 12(34), e06240, 2025]. The inappropriate comment on Zhao et al. has been deleted. An additional comment on Zhao et al. has been supplemented.

Action taken:

- The sentence "*In 2025, Zhao et al.*[36] *established an end-to-end workflow for transition state search to benchmark seven MLP models against the React-OT generative model. The results demonstrated that React-OT outperformed MLP-based transition state search, achieving a higher success rate in locating transition states. The poor performance of these MLP models may be attributed to their lack*

of training on transition state structures and reactive PES." **has been added** (see **Lines 87-91** in the revised manuscript).

- The inappropriate comment "*More critically, the React-OT model generates transition state structures that lack rigorous validation through imaginary frequency analysis and Intrinsic Reaction Coordinate (IRC) verification, and cannot directly predict transition state energies, thereby restricting its ability to predict reaction kinetic properties.*" **has been deleted** (see **Line 71** in the revised manuscript).

2. MACE is known to be slow. I am wondering the number of parameters of the MACE model that the author used and the number of step of optimizations.

Clarification:

To improve the training and inference efficiency of MACE, acceleration via cuEquivariance (an NVIDIA-developed CUDA-accelerated library for equivariant neural networks) was utilized. This library provides custom CUDA kernels that speed up key equivariant operations in MACE, thereby reducing the computational overhead during both training and inference.

The number of parameters of DeePEST-OS is 8.23M. For the DORTS-1K dataset (1,000 novel reactions not included in DORTS-9K), transition state optimization converges in an average of 119 steps. More details on computational cost, convergence steps, and transition state energy for each sample in DORTS-1K are available at https://github.com/kaipai-ren/DeePEST-OS/tree/main/dataset/.

Action taken:

- The sentence "*Note that the NVIDIA cuEquivariance library, a CUDA-accelerated library tailored for equivariant neural networks, was used to accelerate MACE training and inference in this work.*" **has been added** (see **Lines 661-664** in the revised manuscript).

Reviewer 2:

I co-reviewed this manuscript with one of the reviewers who provided the listed reports. This is part of the Nature Communications initiative to facilitate training in peer review and to provide appropriate recognition for Early Career Researchers who co-review manuscripts.

Clarification:

We sincerely appreciate your valuable contribution as a co-reviewer. We are also grateful for your constructive and insightful comments, all of which have been carefully addressed in the revised manuscript.

Reviewer 3:

Comments to the Author:

The paper presents a machine-learning (ML) model targeted at predicting reaction barriers for organic chemical reactions. The ML model generated is based on the widely-used MACE architecture, using a "delta-ML" approach to boost accuracy. Compared to previous ML models, the approach described here seeks to expand the range of element types that can be treated. Overall, the structure of the paper is appropriate to the topic - comments about the writing and figures are given below. The conclusions presented are supported by the available data given.

A first major comment is that the authors should be clearer about the novelty of their approach relative other other ML models, and should also place the predictive capability of their model in the context of actual experiments. The authors have generated a large dataset of organic reactions using textbook organic chemical transformations, then fit a MACE model to the DFT data for these reactions. This fitting works well - one should probably expect so, given that it's a "delta-ML" model based on a decent baseline. The MACE model is already widely used, and previous work has already demonstrated use of MACE to calculate reaction barriers (J. Chem. Phys. 162, 114122 (2025)). So - being clearer about the novelty of this particular ML strategy is important and requires more discussion. More broadly, the research presented here does nothing to address the problem with ML models that is almost-always overlooked - you're training against DFT data that will have errors relative to experiment, so how do you know that your ML model is actually any good in making "real world" predictions?

Clarification:

Thank you so much for your valuable and constructive comments.

This work presents two pivotal contributions. First, we have developed a hybrid data preparation strategy that cuts computational costs by reducing exhaustive conformational sampling to merely 0.01% of full DFT workflows, thereby constructing DORTS, a novel database containing approximately 75,000 diverse reactions and their transition states. When compared to the commonly-used Transition1x database, which includes around 10,000 reactions across 4 elements, DORTS offers expanded coverage with 75,000 versus 10,000 reactions and 10 versus 4 elements, along with richer reaction diversity that incorporates previously unconsidered A+B→C+D reaction types. Second, DeePEST-OS achieves high accuracy by integrating physical priors from semi-empirical quantum chemistry with the MACE architecture within a Δ-learning framework, yielding a root mean square deviation (RMSD) of 0.12 Å for transition state geometries and a mean absolute error (MAE) of 0.60 kcal/mol for reaction energy barriers across DORTS-1K (1,000 novel reactions not included in DORTS-9K). The novelty of this work is explicitly stated in the Introduction.

Regarding the literature [J. Chem. Phys., 162, 114122, 2025], its MACE models were trained on the QMrxn20 E2/SN2 dataset, comprising reactions with anionic nucleophiles/bases. The authors validated these MACE models on an SN2 example and a more complex reaction involving nucleophilic aromatic substitution and proton transfer. However, the reaction types explicitly demonstrated in this literature are limited. As noted by the authors themselves, extending their MACE models to more chemically diverse reaction classes remains an open question. In contrast, our DeePEST-OS exhibits broader applicability across multiple reaction types. It covers four common reaction categories of substitution, addition, ring-opening/closing, and rearrangement reactions, thus expanding the model application scope and enabling predictions for a wider range of reaction types. For architecture comparison, we introduced a delta-learning strategy that integrates the prior physical knowledge from the semi-empirical quantum chemistry method (GFN2-xTB) with the MACE architecture. Evaluated on DORTS-1K (1,000 novel reactions not included in DORTS-9K), our MACE_deltaL variant delivered superior performance in optimizing transition state geometries and predicting reaction energy barriers. Specifically, it reduced the mean geometric RMSD from 0.19 Å (pure MACE) to 0.12 Å, and decreases the MAE in reaction energy barriers from 0.93 (pure MACE) to 0.60 kcal/mol, representing

respective reductions of 36.8% and 35.5%. The integration of prior physical knowledge (GFN2-xTB) effectively mitigates the overfitting risk inherent to the pure MACE model and improves extrapolation ability. Therefore, compared to the work reported in [J. Chem. Phys., 162, 114122, 2025], our study achieves advancements in both model application scope and extrapolation performance.

Regarding the capability of our DeePEST-OS to predict real experimental data, we first acknowledge that achieving quantitative agreement between DFT calculations and experimental results is often challenging, representing a common issue in computational chemistry. Notably, prior research ([Cell Rep. Phys. Sci., 5, 102043, 2024] and [Sep. Purif. Technol., 360, 130984, 2025]) has demonstrated that qualitative agreement with experimental values is attainable when conformational isomerism is rigorously considered and appropriate DFT levels are selected. In this work, transition state geometry optimization and energy calculations via DeePEST-OS have considered conformational isomerism and utilized a high-precision DFT level (ωB97M-V/def2-TZVP, [J. Chem. Phys., 144, 214110, 2016]). Consequently, qualitative consistency with experimental observations is anticipated. Furthermore, incorporating correction strategies, such as linear calibration, can facilitate quantitative alignment with experiments. To validate this perspective, a Diels-Alder reaction involving endo/exo diastereoselectivity is taken as an example. After considering transition state conformational isomers, the Gibbs free energy barrier difference ($\Delta\Delta G$) between endo and exo transition states was calculated for 11 reactants. Least-squares linear regression was then applied to correlate these calculated $\Delta\Delta G$ values with experimental diastereomeric excess (*de*) values. As illustrated in **Fig. 8(b)**, the coefficient of determination (R^2) for the correlation between DFT-calculated $\Delta\Delta G$ and experimental *de* reached 0.82, indicating that this DFT benchmark reliably predicts the diastereoselectivity of these Diels-Alder reactions. To further evaluate the DeePEST-OS, **Fig. 8(c)** displays the correlation between DeePEST-OS-predicted $\Delta\Delta G$ values and experimental *de* values. The resulting R^2 of 0.76 demonstrates a reasonably strong quantitative correlation. Collectively, these results indicate that both DFT and our DeePEST-OS model are able to make "real world" predictions.

Action taken:

- The sentence "*Two pivotal contributions are presented in this work to address the limitations in existing studies. First, a hybrid data preparation strategy is developed to construct a large-scale Database of Organic Reaction Transition*

States (DORTS) in a cost-effective manner. Compared to the Transition1x dataset, DORTS provides substantially broader chemical diversity in organic reactions. Second, DeePEST-OS (Deep learning-based molecular Potential Energy Surface prediction Tool for Organic Synthesis) is proposed by integrating prior physical knowledge from GFN2-xTB into the MACE architecture via a Δ-learning strategy[43]*, enabling accurate predictions of transition state geometries and energy barriers with robust extrapolation capabilities across diverse reaction scenarios.*" **has been added** (see **Lines 107-113** in the revised manuscript).

- The part "*Experimental validation of endo and exo diastereoselectivity in Diels-Alder reactions*" **has been added** (see **Line 517** in the revised manuscript).

- The paragraph "*To evaluate the error between DeePEST-OS predictions and experimental data, the endo/exo diastereoselectivity of the Diels-Alder reaction was tested as a case study. Experimental diastereomeric excess (de) values were sourced from the literature*[60]*, and theoretically, de is fundamentally correlated with the Gibbs free energy barrier difference (ΔΔG) between the endo and exo insertions. To predict these ΔΔG values, DFT calculations were employed at the ωB97M-V/def2-TZVP level, as this DFT level maintains consistency with the DeePEST-OS training database (DORTS-9K) and exhibits excellence in predicting thermodynamic properties*[52]*. Furthermore, conformational isomerism was explicitly considered, since it has been shown to improve the quantitative agreement between theoretical calculations and experimental observations*[61,62]*. After evaluating the ΔΔG values for 11 distinct reactants, a least-squares linear regression was performed to correlate the DFT-calculated values with the experimental de values, as depicted in* ***Fig. 8(b)****. The resulting coefficient of determination (R^2) was 0.82, demonstrating that this benchmark DFT methodology has the capacity to provide reliable predictions of experimental de trends for the endo/exo diastereoselectivity of the Diels-Alder reaction.*" **has been added** (see **Lines 518-529** in the revised manuscript).

- The paragraph "*To further evaluate DeePEST-OS,* ***Fig. 8(c)*** *displays the correlation between DeePEST-OS-predicted ΔΔG values and experimental de values. The resulting R^2 of 0.76 demonstrated a reasonably strong quantitative correlation. Although this R^2 was slightly lower than that achieved by the DFT method, DeePEST-OS preserves the DFT-inherent predictive capacity for de*

values to facilitate experimental exploration." **has been added** (see **Lines 530-533** in the revised manuscript).

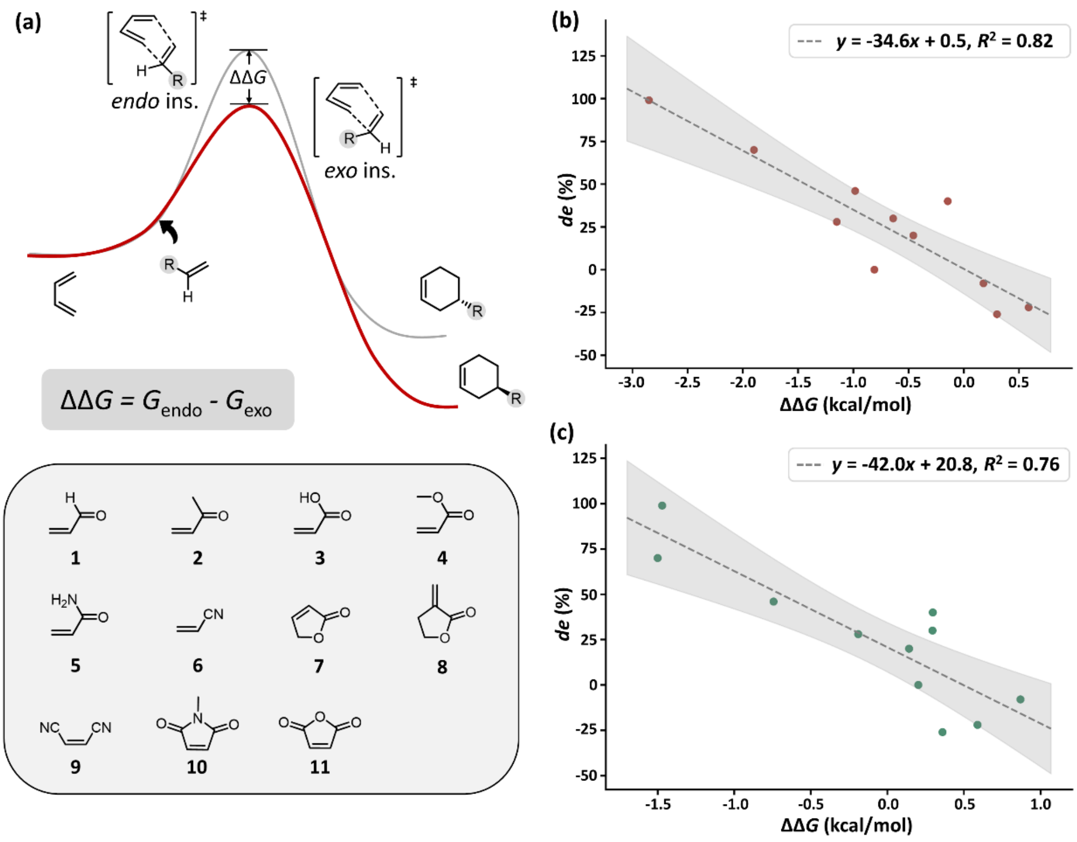

Fig. 8 Linear fitting between ΔΔ*G* and *de* values for endo and exo insertions of different reactants in Diels-Alder reactions. (a) ΔΔ*G* definition and reactant structures; **(b)** Linear fitting between DFT-calculated ΔΔ*G* and experimental *de* values; **(c)** Linear fitting between DeePEST-OS-calculated ΔΔ*G* and experimental *de* values.

A second key comment: the writing in the paper has a number issues to correct, and is prone to hyperbole (which can very distracting throughout). For example, as noted below, there are some factually incorrect statements, including in the abstract. The authors should make sure to address these throughout.

Clarification:

We greatly appreciate your careful reading of our manuscript, as well as your valuable comments. In the following we respond to each of the comments.

More detailed comments are as follows:

1. In Abstract - DFT is not a "method for transition-state searches". It is a method for calculating energy.

Clarification:

Thank you so much for this suggestion. We have revised the inaccurate statement in the Abstract and conducted a systematic review of the entire manuscript to ensure no similar issues remain.

Action taken:

- The sentence "*Precise understanding of reaction kinetics, requiring accurate transition states and energy barriers, is central to organic synthesis. While Density Functional Theory (DFT) remains the mainstream method for transition state searches, its inherent trade-offs between accuracy and computational cost persist.*" **has been revised to** "*Precise understanding of reaction kinetics is fundamental to organic synthesis, yet it is often hindered by the high computational cost of performing transition state searches at the Density Functional Theory (DFT) level.*" (see **Lines 14-16** in the revised manuscript).

2. In Abstract, the authors note that DFT has a trade-off between accuracy and cost. A ML model does nothing to address accuracy - but this is a stated target of the author's work (as noted in the first sentence of the abstract).

Clarification:

Thank you so much for your valuable correction. The inappropriate statement has been corrected.

Action taken:

- The sentence "*Precise understanding of reaction kinetics, requiring accurate transition states and energy barriers, is central to organic synthesis. While Density Functional Theory (DFT) remains the mainstream method for transition state searches, its inherent trade-offs between accuracy and computational cost persist.*" **has been revised to** "*Precise understanding of reaction kinetics is fundamental to organic synthesis, yet it is often hindered by the high computational cost of performing transition state searches at the Density Functional Theory (DFT) level.*" (see **Lines 14-16** in the revised manuscript).

3. A ML potential cannot be both "generic" and yet specifically cover 10 element types - reword.

Clarification:

Thank you so much for your correction. We have removed the inappropriate term "generic" throughout the revised manuscript.

Action taken:

- The title "*Generic Machine Learning Potential for Accelerating Transition State Search in Organic Synthesis*" **has been revised to** "*Reactive Machine Learning Potential for Accelerating Transition State Search in Organic Synthesis*".

- The sentence "*To bridge this gap, a generic machine learning potential (DeePEST-OS) covering ten element types is developed for rapid and precise transition state searches in organic synthesis.*" **has been revised to** "*This work introduces DeePEST-OS, a reactive machine learning potential designed for rapid and precise prediction of potential energy surfaces and transition state optimization across ten elements.*" (see **Line 16** in the revised manuscript).

4. The level of hyperbole and over-emphasis in the paper is generally distracting - examples include "remarkable accuracy", "significantly advancing the acceleration of QC calculations", "expansive realm", "remarkable diversity", "As a classic textbook and authoritative reference book in the field of organic chemistry, March's Advanced Organic Chemistry…", "…deeply coupling the mechanism driven semi-empirical…", "This breakthrough in chemical intelligence underscores…", "This advancement elevates organic reaction kinetics prediction to a new synergistic paradigm…". These sorts of phrases are not necessary and should be toned down.

Clarification:

Thank you so much for your careful reading and valuable suggestion. A thorough review of the manuscript has been conducted, and all necessary revisions have been implemented.

Action taken:

- The sentence "*DeePEST-OS delivers remarkable accuracy…*" **has been revised to** "*DeePEST-OS demonstrated high accuracy…*" (see **Line 23** in the revised manuscript).
- The sentence "*while significantly reducing computational costs*" **has been revised to** "*while reducing computational costs*" (see **Line 55** in the revised manuscript).
- The sentence "*fundamentally transform the discovery of new reactions…*" **has been revised to** "*transform the discovery of new reactions…*" (see **Line 58** in the revised manuscript).
- The sentence "*significantly advancing the acceleration of QC calculations…*" **has been revised to** "*advancing the acceleration of QC calculations…*" (see **Line 76** in the revised manuscript).
- The sentence "*achieving remarkable accuracy across various benchmark datasets…*" **has been revised to** "*achieving high accuracy across various benchmark datasets…*" (see **Line 82** in the revised manuscript).
- The sentence "*significantly outperforming semi-empirical QC methods…*" **has been revised to** "*outperforming semi-empirical QC methods…*" (see **Line 116** in the revised manuscript).
- The sentence "*Within the expansive realm of organic chemistry, organic reactions exhibit remarkable diversity…*" **has been revised to** "*Organic reactions exhibit high diversity…*" (see **Line 136** in the revised manuscript).
- The sentence "*A thorough investigation of any single organic reaction network invariably yields tens of thousands of distinct reactions.*" **has been revised to** "*A thorough investigation of any single organic reaction network may yield tens of thousands of distinct reactions.*" (see **Line 136** in the revised manuscript).
- The sentence "*…through network exploration fundamentally impractical.*" **has been revised to** "*…through network exploration impractical.*" (see **Line 138** in the revised manuscript).
- The sentence "*which significantly dilute the overall quality of the dataset.*" **has been revised to** "*which dilute the overall quality of the dataset.*" (see **Line 139** in the revised manuscript).

- The sentence "*As a classic textbook and authoritative reference book in the field of organic chemistry, March's Advanced Organic Chemistry…*" **has been revised to** "*March's Advanced Organic Chemistry…*" (see **Line 145** in the revised manuscript).
- The sentence "*To systematically investigate substituent effects*" **has been revised to** "*To investigate substituent effects*" (see **Line 154** in the revised manuscript).
- The sentence "*along with significantly increased data volume.*" **has been revised to** "*along with increased data volume.*" (see **Line 210** in the revised manuscript).
- The sentence "*The resultant energy landscape demonstrates uniform continuity with systematically aligned energy progression gradients*" **has been revised to** "*The resultant energy landscape demonstrated uniform continuity with aligned energy progression gradients*" (see **Line 220** in the revised manuscript).
- The sentence "*Reference energy baselines for individual atomic types are systematically tabulated in supporting information Table S1*" **has been revised to** "*Reference energy baselines for individual atomic types are tabulated in supporting information **Tab. S2***" (see **Line 222** in the revised manuscript).
- The sentence "*...show significantly broader atomic force distributions…*" **has been revised to** "*...showed broader atomic force distributions…*" (see **Line 235** in the revised manuscript).
- The sentence "*The proposed hybrid data preparation strategy significantly reduces the computational costs…*" **has been revised to** "*The proposed hybrid data preparation strategy reduces the computational costs…*" (see **Line 253** in the revised manuscript).
- The sentence "*offers outstanding computational efficiency advantages…*" **has been revised to** "*offers computational efficiency advantages…*" (see **Line 273** in the revised manuscript).
- The sentence "*deeply coupling the mechanism-driven semi-empirical QC methods…*" **has been revised to** "*coupling the mechanism-driven semi-empirical QC methods…*" (see **Line 274** in the revised manuscript).
- The sentence "*This breakthrough stems from the unique generalizability advantage of GFN2-xTB*" **has been revised to** "*This breakthrough stems from the*

generalizability advantage of GFN2-xTB" (see **Line 294** in the revised manuscript).

- The sentence "*This leads to a significant reduction in the RMSE of PES prediction…*" **has been revised to** "*This led to a reduction in the RMSE of PES prediction…*" (see **Line 300** in the revised manuscript).
- The sentence "*This improvement in accuracy fundamentally reflects the complementarity of…*" **has been revised to** "*This improvement in accuracy reflects the complementarity of…*" (see **Line 302** in the revised manuscript).
- The sentence "*MACE accurately fits the local fine-grained features of the DFT-calculated PES*" **has been revised to** "*MACE fits the local fine-grained features of the DFT-calculated PES*" (see **Line 303** in the revised manuscript).
- The sentence "*The synergy between the two significantly enhances the model…*" **has been revised to** "*The synergy between the two enhances MACE_deltaL…*" (see **Lines 304** in the revised manuscript).
- The sentence "*significantly outperforming the GFN2-xTB method*" **has been revised to** "*outperforming the GFN2-xTB method*" (see **Line 316** in the revised manuscript).
- The sentence "*the critical role of conformational isomers on reaction energy barriers is decisive*" **has been revised to** "*conformational isomers play a critical role in determining reaction energy barriers*" (see **Line 445** in the revised manuscript).
- The sentence "*Both methods demonstrate remarkable consistency in conformational rankings*" **has been revised to** "*Both methods demonstrated consistency in conformational rankings*" (see **Line 463** in the revised manuscript).
- The sentence "*This breakthrough in chemical intelligence underscores the potential for applications in complex organic synthesis*" **has been revised to** "*DeePEST-OS shows potential for applications in complex organic synthesis*" (see **Line 504** in the revised manuscript).
- The sentence "*...accuracy and computational efficiency has fundamentally limited…*" **has been revised to** "*...accuracy and computational efficiency has limited…*" (see **Line 688** in the revised manuscript).

- The sentence "*DeePEST-OS leverages unprecedented coverage of ten elements to…*" **has been revised to** "*DeePEST-OS leverages coverage of ten elements to…*" (see **Line 705** in the revised manuscript).

- The sentence "*This uniquely broad applicability delivers DFT-level accuracy…*" **has been revised to** "*This broad applicability delivers DFT-level accuracy…*" (see **Line 707** in the revised manuscript).

- The sentence "*By definitively resolving the accuracy-efficiency dilemma…*" **has been revised to** "*By addressing the accuracy-efficiency dilemma…*" (see **Line 711** in the revised manuscript).

5. Line 33 - "with essential applications in fields." is a vague sentence that should be replaced.

Clarification:

We greatly appreciate your suggestions. We have revised this sentence to provide specific examples and clarify the importance of organic synthesis applications.

Action taken:

- The sentence "*Organic synthesis is a fundamental aspect of modern chemistry, enabling the creation of a wide variety of molecules with essential applications in fields.*" **has been revised to** "*Organic synthesis is a fundamental aspect of modern chemistry, enabling the creation of a wide variety of molecules with essential applications in medicine, materials science, and so on*[1-3]." (see **Line 35** in the revised manuscript).

6. Line 33/34 - How does reaction kinetics help "control the rates of chemical reactions"? Reword.

Clarification:

Thank you so much for this valuable comment. We have rephrased this sentence.

Action taken:

- The sentence "*The role of reaction kinetics in organic synthesis is crucial, as it helps explain and control the rates of chemical reactions*" **has been revised to** "*The role of reaction kinetics in organic synthesis is crucial, as it helps explain*

reaction rates, predict their dependence on reaction conditions, and thereby optimize conditions for greater efficiency" (see **Line 36** in the revised manuscript).

7. Semi-empirical methods like XTB and DFTB can behave extremely poorly for reactions - for example, predicting multiple minima along a reaction path or even predicting transition-states that don't lead to the expected products/reactants. So, it's not just the barriers and geometries that can be untrustworthy, but the entire character of the reactions under study. How do the authors address this in their work?

Clarification:

We agree with the comment that semi-empirical methods such as xTB and DFTB exhibit inferior performance to DFT in certain scenarios, including the prediction of spurious minima on reaction paths or transition states that deviate from expected reaction coordinates. While the accuracy of xTB is indeed lower than that of DFT, its reliability and generalizability in preliminary computational studies are well-documented in the relevant literature ([Commun. Chem., 8, 255,)2025] and [J. Chem. Theory Comput., 22(1), 422-440, 2026]). Specifically, xTB has been recognized as a valuable tool for pre-optimizing reaction geometries and conducting initial explorations of reaction pathways.

Consistent with these findings, the applicability of xTB for pre-optimization has also been validated in our work. We compared xTB-derived pre-optimized geometries with the high-level DFT-optimized structures (**Fig. 5(a)**). The mean RMSD of transition state geometries optimized by GFN2-xTB was 0.31 Å, demonstrating that this method achieves reasonable accuracy for organic systems.

To further enhance the accuracy of xTB with precision approaching that of DFT methods while preserving its computational efficiency, this study employs a Δ-learning strategy. Specifically, the deep learning-based MACE model is trained to learn the deviation between DFT and GFN2-xTB predictions. This learned deviation is subsequently added to the GFN2-xTB baseline predictions, refining the final outputs to closely match DFT outcomes. By leveraging this approach, we obtained an efficient machine learning potential model (i.e., MACE_deltaL model) with DFT-level accuracy, where the error relative to DFT predictions is illustrated in **Fig. 4(a)**. Importantly, compared to a standalone MACE model trained directly on DFT data, the proposed MACE_deltaL model demonstrates superior extrapolation performance on DORTS-1K (1,000 novel reactions not included in the training set), as shown in **Fig. 5(a-b)**. This

enhancement is primarily attributed to the physical knowledge embedded in GFN2-xTB, which strengthens the generalization capability of the MACE model.

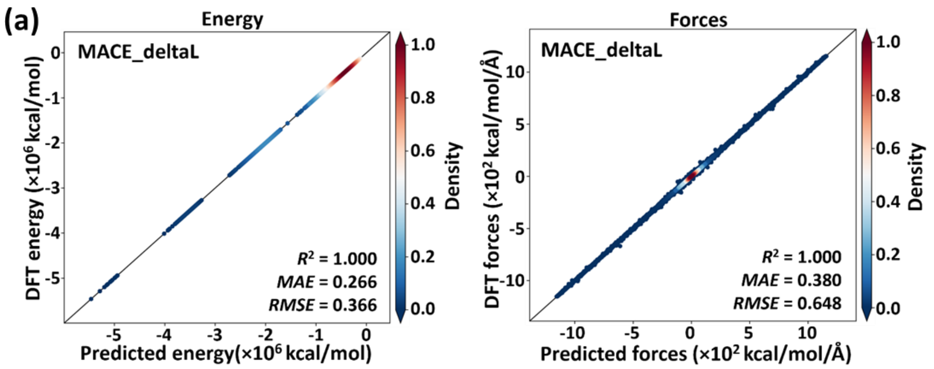

Fig. 4 Model training results and comparisons. (a) Performance of the MACE_deltaL model.

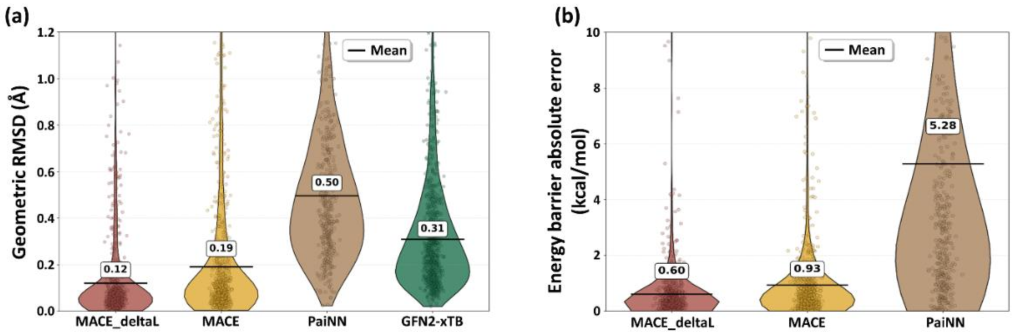

Fig. 5 Accuracy and computational cost evaluations based on the test set. (a) Violin plot illustrating the distribution of transition state geometric RMSDs for various methods based on DORTS-1K. **(b)** Violin plot depicting the distribution of absolute errors in predicted reaction energy barriers across different methods based on DORTS-1K.

8. The cartoon characters in Figure 1 are not appropriate and should be removed.

Clarification:

Thank you for your kind reminder. The cartoon characters in **Fig. 1** have been removed.

Action taken:

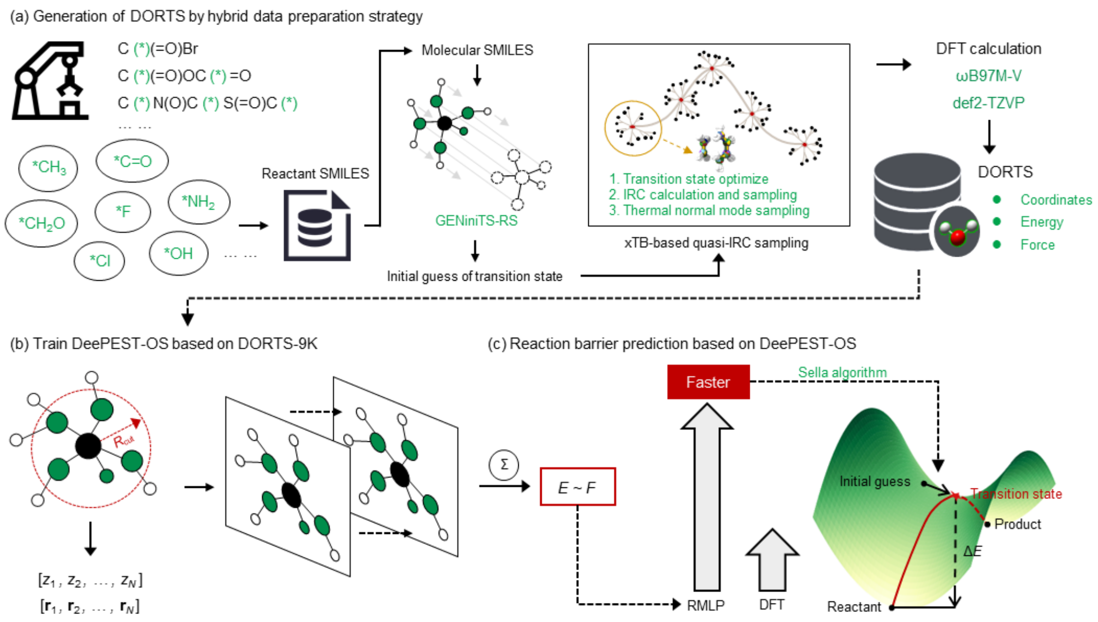

Fig. 1 Workflow of developing DeePEST-OS. (a) Generation of the DORTS database using a hybrid data preparation strategy. (b) Training DeePEST-OS on the DORTS-9K database. (c) Prediction of reaction energy barriers using DeePEST-OS.

9. Line 138 - by limiting the generated database to only those previously-known reactions extracted from a textbook, does this limit the scope of the trained model so that it cannot identify truly novel chemistry? Also, the reaction templates/types should be listed somewhere - they don't seem to be included.

Clarification:

First, to address concerns regarding the model capacity to identify novel chemistry, we have supplemented an Out-Of-Distribution (OOD) test to evaluate the extrapolation capability of our MACE_deltaL model (trained on the DORTS-9K dataset) using reactions that are never seen during training and chemically distinct from known reaction categories in DORTS-9K. The OOD dataset comprises 1,073 reactions selected from Transition1x, a widely used machine learning potential training database that differs from DORTS-9K. This distinction is visualized in the chemical space plot (**Fig. 3(c)**). The OOD test results showed a mean RMSD of 0.11 Å for transition state geometries and an MAE of 3.80 kcal/mol for reaction energy barriers. While these metrics indicate a modest decline in accuracy relative to the performance on the DORTS-1K test set—attributable to the notable structural differences between the Transition1x and DORTS-9K datasets that pose challenges for extrapolation—the predictive accuracy of our MACE_deltaL model remains highly competitive with that

of the OA-ReactDiff model (a mean geometric RMSD of 0.129 Å and an MAE of 4.4 kcal/mol for reaction energy barriers) [Nat. Comput. Sci., 3, 1045-1055, 2023]. Note that OA-ReactDiff was specifically trained on the Transition1x dataset, whereas our MACE_deltaL model has not trained on it. Collectively, these results demonstrate the robust extrapolation ability of our MACE_deltaL model.

Second, detailed reaction template information, represented by SMARTS strings, is now provided in **Tab. S1** of the revised supporting information. In addition, we have corrected the stated number of templates from 294 to 255 and sincerely apologize for this numerical error in the original manuscript. All computational data in the previous submission (including the database, models, etc.) were generated using these 255 reaction templates. Therefore, this correction does not affect any reported results in the original manuscript. We have also reviewed the entire manuscript to avoid similar issues.

Action taken:

- The sentence "*In this study, 294 fundamental reactions are extracted from this book and encoded as the SMARTS...*" **has been revised to** "*In this study, 255 fundamental reactions were extracted from this book and encoded as the SMARTS...*" (see **Line 146** in the revised manuscript).
- The sentence "*with the reaction templates provided in **Tab. S1** of the supporting information*" **has been added** (see **Line 151** in the revised manuscript).
- The sentence "*To validate the extrapolation ability of the MACE_deltaL model, it is essential to test its performance on Out-Of-Distribution (OOD) reactions. As illustrated in the chemical space in **Fig. 3(c)**, the reactions in the Transition1x (red dots) and DORTS-9K training set (blue dots) exhibit limited overlap and occupy distinct regions, indicating reaction dissimilarity.*" **has been added** (see **Lines 373-375** in the revised manuscript).
- The sentence "*In this rigorous OOD test, a mean RMSD of 0.11 Å for transition state geometries and an MAE of 3.80 kcal/mol for reaction energy barriers were achieved by the MACE_deltaL model (**Fig. 5(f)**). While these metrics indicate a modest decline in accuracy relative to the performance on the DORTS-1K test set—attributable to the notable structural differences between the Transition1x and DORTS-9K datasets that pose challenges for extrapolation—the predictive*

accuracy of our MACE_deltaL model remains highly competitive with that of the OA-ReactDiff model (a mean geometric RMSD of 0.129 Å and an MAE of 4.4 kcal/mol for reaction energy barriers)[55], which was specifically trained on the Transition1x dataset. These results demonstrate the robust extrapolation ability of the MACE_deltaL model." **has been added** (see **Lines 379-386** in the revised manuscript).

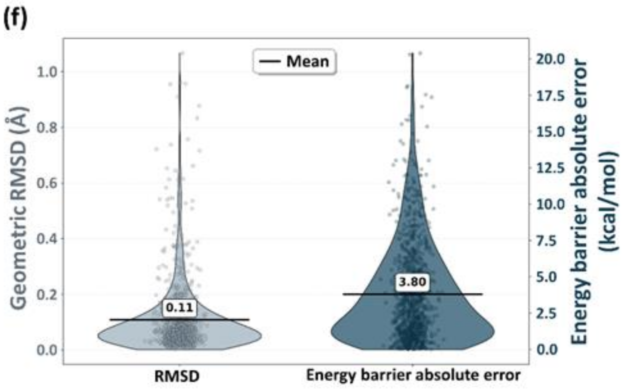

Fig. 5 Accuracy and computational cost evaluations based on the test set. (f) Violin plot depicting the distribution of transition state geometric RMSDs and absolute errors in predicted reaction energy barriers for 1,073 reactions in Transition1x by MACE_deltaL.

10. From the description of methodology, it sounds like transition-state geometries are only determined using XTB? Or are they optimised again using DFT?

Clarification:

During dataset preparation, we utilized the GFN2-xTB method for transition state searches, IRC calculations, and normal mode sampling, without using DFT methods to search transition states. Considering that GFN2-xTB struggles to identify transition states with DFT-level accuracy during sampling, we validated all xTB-derived transition states via IRC calculations and rejected any that failed validation. Meanwhile, the normal mode sampling generates molecular conformations around the IRC pathways, enriching the reaction pathway information for machine learning potential model development. In contrast to full DFT-based workflows that demand substantial computational resources for data preparation, this xTB-based quasi-IRC sampling strategy reduces the cost of data preparation while enabling the development of accurate machine learning potential models, with their performance demonstrated in **Fig. 4(a)**.

In the subsequent data labeling stage, we performed single-point energy calculations on the sampled molecular conformations using DFT (ωB97M-V/def2-TZVP) to generate high-precision labels (molecular energies and atomic force matrices) for training machine learning potential models.

For model performance evaluation, we optimized transition states in the test set samples using both our machine learning potential model and the benchmark DFT method (ωB97M-V/def2-TZVP) to rigorously evaluate the predictive accuracy of our developed machine learning potential model **(Fig. 5(a-b)**).

11. Line 246 - the relative times of generating datasets with XTB and DFT don't "…highlight the efficiency of the proposed strategy." - they simply reflect that fact that XTB is faster than DFT. Reword.

Clarification:

We appreciate your insightful comment. The time-cost advantage of this hybrid data preparation strategy stems from replacing DFT with the GFN2-xTB method to generate rich conformations along the reaction path. The revised manuscript now clarifies that this advantage stems from the inherent computational efficiency of GFN2-xTB.

Action taken:

- The sentence "*The time with the strategy accounts for approximately 0.01% of that without the strategy, highlighting the efficiency of the proposed strategy*" **has been revised to** "*The inherent computational efficiency of GFN2-xTB itself enabled the time with the strategy to account for approximately 0.01% of that without the strategy, highlighting the efficiency of this hybrid data preparation strategy*" (see **Line 261** in the revised manuscript).

12. Line 267 - it would be useful to know where the improvements over the "basic" MACE (without delta-ML) come from? Are there specific reaction types that can be identified that are treated more accurately in the Deepest-OS model? Some further analysis would be interesting.

Clarification:

We greatly appreciate your insightful comments. To elucidate the origins of the improvements over the basic MACE (without Δ-learning), we analyzed the prediction

errors for transition state geometric RMSDs and reaction energy barriers on the DORTS-1K dataset with respect to the number of atoms and four reaction classes (substitution, addition, ring-opening/closing, and molecular rearrangement).

Specifically, consistent performance gains were observed across all four reaction classes after incorporating Δ-learning, indicating that it provides a broadly positive effect rather than benefiting only a narrow subset of reactions (**Fig. S2**).

Fig. 5(c) presents the comparison of MACE and MACE_deltaL prediction errors (mean geometric RMSD and the MAE of reaction energy barriers) on DORTS-1K with respect to the number of atoms. After incorporating Δ-learning, both the mean geometric RMSD and the MAE of reaction energy barriers exhibited a general decrease, with the magnitude of this error reduction increasing as the number of atoms grew. This result likely stems from the tendency of larger molecular systems, with increasing atomic count, to exhibit more long-range interactions. Incorporating the prior physical knowledge of GFN2-xTB effectively compensates for the inherent limitations of pure machine learning potential models (e.g., MACE) in capturing such long-range interactions.

Action taken:

- The sentence "*To investigate how MACE_deltaL achieves its performance gains over pure MACE, its prediction errors for transition state geometric RMSDs and reaction energy barrier absolute errors on DORTS-1K were systematically analyzed and stratified by both reaction class and atom count. Specifically, for reaction class incorporating Δ-learning yielded consistent gains across all four reaction classes (substitution, addition, ring-opening/closing, and molecular rearrangement), indicating a broad rather than niche benefit **(Fig. S2)**. For atom count, **Fig. 5(c)** presents the comparison of MACE and MACE_deltaL prediction errors (mean geometric RMSD and the MAE of reaction energy barriers) on DORTS-1K with respect to the number of atoms. After incorporating Δ-learning, both the mean geometric RMSD and the MAE of reaction energy barriers exhibited a general decrease, with the magnitude of this error reduction increasing as the number of atoms grew. This result likely stems from the tendency of larger molecular systems, with increasing atomic count, to exhibit more long-range interactions. Incorporating the prior physical knowledge of GFN2-xTB effectively compensates for the inherent limitations of pure MLP models (e.g.,*

MACE) in capturing such long-range interactions." **has been added** (see **Lines 348-359** in the revised manuscript).

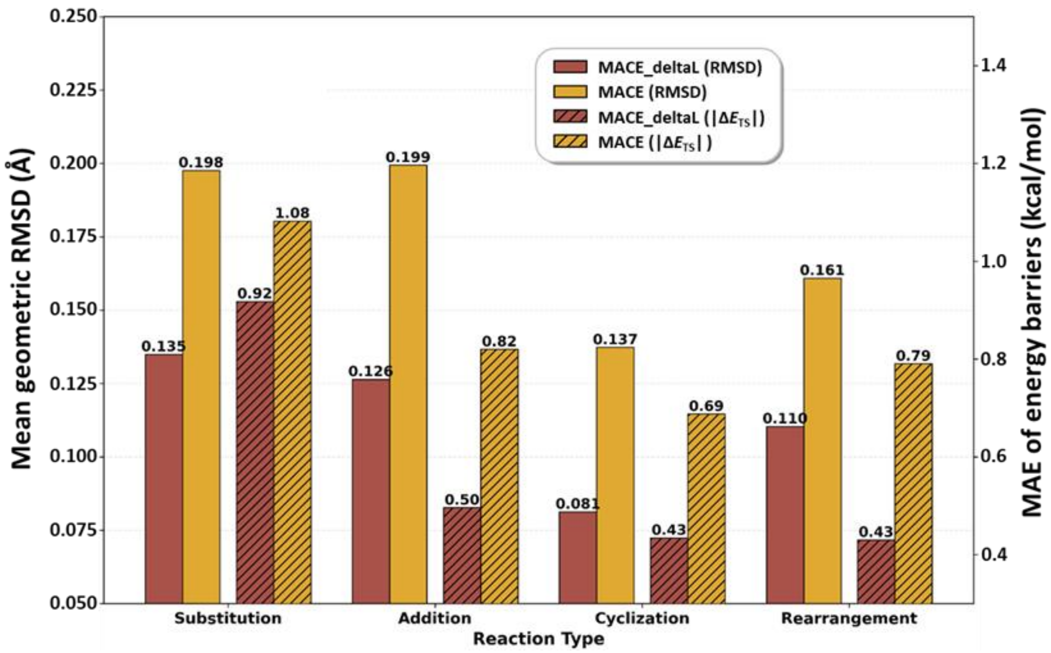

Fig. S2 Comparison of MACE and MACE_deltaL prediction errors (mean geometric RMSD and the MAE of reaction energy barriers) on DORTS-1K with respect to four reaction classes (substitution, addition, ring-opening/closing, and molecular rearrangements).

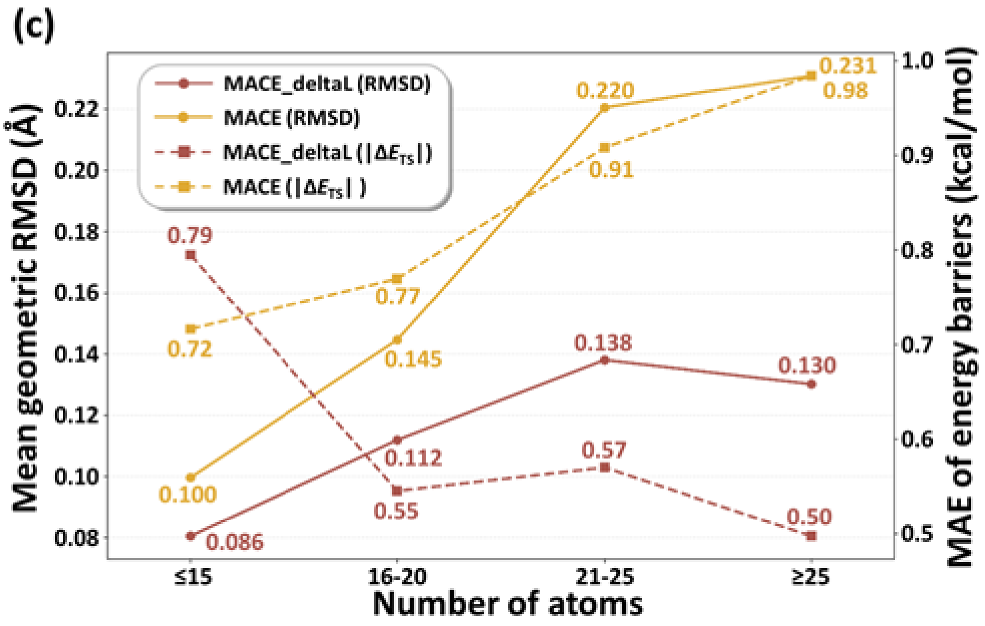

Fig. 5 Accuracy and computational cost evaluations based on the test set. (c) Comparison of MACE and MACE_deltaL prediction errors (mean geometric RMSD

and the MAE of reaction energy barriers) on DORTS-1K with respect to the number of atoms.

13. Line 294 - "which is widely considered one of the best open-source transition state search algorithms currently available." - these sorts of subjective statements should be removed.

Clarification:

Thank you so much for this suggestion. We have removed this subjective statement from the revised manuscript.

Action taken:

- The sentence "*which is widely considered one of the best open-source transition state search algorithms currently available.*" **has been deleted**. (see **Line 308** in the revised manuscript).

14. Line 298 - "Particularly in IRC path calculations, MACE-deltaL (0.11 core·s·step-1) achieves an efficiency breakthrough of four orders of magnitude compared to DFT (2,577.2 core·s·step-1) (Fig. 4(d))". Again, the hyperbole here should be removed - in addition, it is worth noting that these time comparisons are not directly a result of the work reported here - MACE and XTB are just faster than DFT, so it's not clear why the authors are emphasising this point.

Clarification:

We greatly appreciate your insightful suggestion. In the revised manuscript, we have removed the hyperbole "breakthrough" and objectively presented the model speed comparison results. The reason we highlight the speed advantage of our DeePEST-OS model over DFT in transition state geometry optimization is that the key strength of machine learning potential models lies in their ability to achieve computational efficiency comparable to GFN2-xTB, while maintaining accuracy close to DFT. Although MACE and GFN2-xTB are widely recognized as computationally efficient methods, the inference speed of their Δ-learning coupled model (MACE_deltaL) remains unknown. To address this, we evaluated the inference speed of MACE_deltaL and present the corresponding results in the manuscript. Our emphasis is not to claim novelty in achieving fast speeds, but rather to provide validation for the speed performance of the MACE_deltaL model.

Action taken:

- The sentence "*MACE_deltaL (0.11 core·s·step^{-1}) achieves an efficiency breakthrough of four orders of magnitude compared to DFT (2,577.2 core·s·step^{-1}) **(Fig. 4(d))***" **has been revised to** "*MACE_deltaL (0.11 core·s·step^{-1}) achieved four orders of magnitude of acceleration compared to DFT (2,577.2 core·s·step^{-1}) **(Fig. 4(d))**, demonstrating the promising inference efficiency of MACE_deltaL that couples MACE with GFN2-xTB*" (see **Line 311** in the revised manuscript).

15. Line 321 - are the 880 transition-states re-optimized using MACE, Painn, etc.? If not, this comparison does not seem fair, as it's not really comparing TS structures for each model.

Clarification:

Thank you very much for your question. In our original manuscript, we conducted a comparative study by independently re-optimizing 880 initial guesses of transition states using PaiNN, MACE, and GFN2-xTB. We apologize that the original statement was unclear and caused confusion. In the revised manuscript, we have rewritten this statement to improve its clarity.

Action taken:

- The sentence "*These 880 transition states are subsequently re-evaluated using MACE, PaiNN, and GFN2-xTB methods, with DFT-optimized transition state geometries and energy barriers serving as the benchmark.*" **has been revised to** "*The same initial guesses corresponding to these 880 transition states were subsequently re-optimized using MACE, PaiNN, and GFN2-xTB, with DFT-derived transition state geometries and reaction energy barriers serving as the benchmark.*" (see **Line 334** in the revised manuscript).

16. Line 335 - "marking a fitting accuracy better than ±1 kcal/mol for transition state energies.". It is important that the authors discuss the fact that this fitting accuracy is only relative to DFT data - it does NOT reflect absolute accuracy relative to experiment. Can the authors say something about how they expect the barrier accuracy to compare to experimental data? Has this been assessed for their ML model?

Clarification:

We greatly appreciate your valuable suggestion. Regarding the capability of our DeePEST-OS to predict real experimental data, we first acknowledge that achieving quantitative agreement between DFT calculations and experimental results is often challenging, representing a common issue in computational chemistry. Notably, prior research ([Cell Rep. Phys. Sci., 5, 102043, 2024] and [Sep. Purif. Technol., 360, 130984, 2025]) has demonstrated that qualitative agreement with experimental values is attainable when conformational isomerism is rigorously considered and appropriate DFT levels are selected. In this work, transition state geometry optimization and energy calculations via DeePEST-OS have considered conformational isomerism and utilized a high-precision DFT level (ωB97M-V/def2-TZVP, [J. Chem. Phys., 144, 214110, 2016]). Consequently, qualitative consistency with experimental observations is anticipated. Furthermore, incorporating correction strategies, such as linear calibration, can facilitate quantitative alignment with experiments. To validate this perspective, a Diels-Alder reaction involving endo/exo diastereoselectivity is taken as an example. After considering transition state conformational isomers, the Gibbs free energy barrier difference ($\Delta\Delta G$) between endo and exo transition states was calculated for 11 reactants. Least-squares linear regression was then applied to correlate these calculated $\Delta\Delta G$ values with experimental diastereomeric excess (*de*) values. As illustrated in **Fig. 8(b)**, the coefficient of determination (R^2) for the correlation between DFT-calculated $\Delta\Delta G$ and experimental *de* reached 0.82, indicating that this DFT benchmark reliably predicts the diastereoselectivity of these Diels-Alder reactions. To further evaluate the DeePEST-OS, **Fig. 8(c)** displays the correlation between DeePEST-OS-predicted $\Delta\Delta G$ values and experimental *de* values. The resulting R^2 of 0.76 demonstrates a reasonably strong quantitative correlation. Collectively, these results indicate that both DFT and our DeePEST-OS model are able to make "real world" predictions.

Action taken:

- The part "*Experimental validation of endo and exo diastereoselectivity in Diels-Alder reactions*" **has been added** (see **Line 517** in the revised manuscript).

- The paragraph "*To evaluate the error between DeePEST-OS predictions and experimental data, the endo/exo diastereoselectivity of the Diels-Alder reaction was tested as a case study. Experimental diastereomeric excess (de) values were sourced from the literature[60], and theoretically, de is fundamentally correlated with the Gibbs free energy barrier difference ($\Delta\Delta G$) between the endo and exo insertions. To predict these $\Delta\Delta G$ values, DFT calculations were employed at the*

ωB97M-V/def2-TZVP level, as this DFT level maintains consistency with the DeePEST-OS training database (DORTS-9K) and exhibits excellence in predicting thermodynamic properties[52]. Furthermore, conformational isomerism was explicitly considered, since it has been shown to improve the quantitative agreement between theoretical calculations and experimental observations[61,62]. After evaluating the ΔΔG values for 11 distinct reactants, a least-squares linear regression was performed to correlate the DFT-calculated values with the experimental de values, as depicted in ***Fig. 8(b)****. The resulting coefficient of determination (R^2) was 0.82, demonstrating that this benchmark DFT methodology has the capacity to provide reliable predictions of experimental de trends for the endo/exo diastereoselectivity of the Diels-Alder reaction.*" **has been added** (see **Lines 518-529** in the revised manuscript).

- The paragraph "*To further evaluate DeePEST-OS,* ***Fig. 8(c)*** *displays the correlation between DeePEST-OS-predicted ΔΔG values and experimental de values. The resulting R^2 of 0.76 demonstrated a reasonably strong quantitative correlation. Although this R^2 was slightly lower than that achieved by the DFT method, DeePEST-OS preserves the DFT-inherent predictive capacity for de values to facilitate experimental exploration.*" **has been added** (see **Lines 530-533** in the revised manuscript).

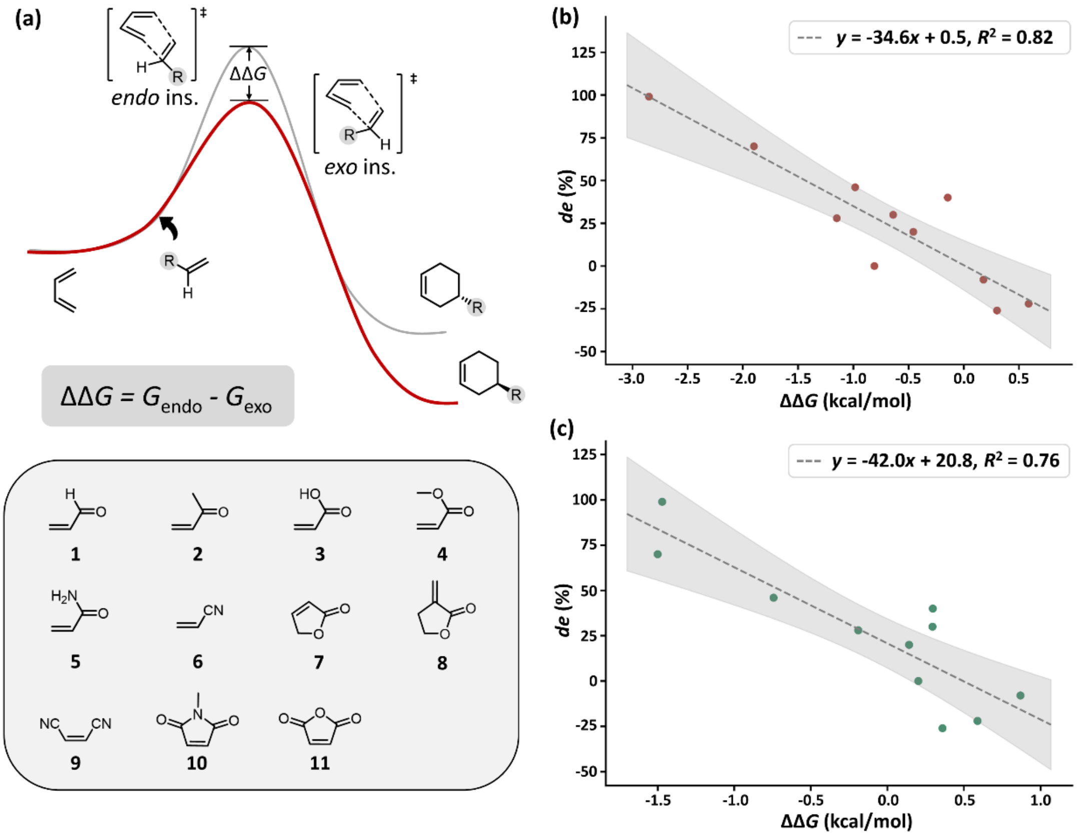

Fig. 8 Linear fitting between ΔΔ*G* and *de* values for endo and exo insertions of different reactants in Diels-Alder reactions. (a) ΔΔ*G* definition and reactant structures; **(b)** Linear fitting between DFT-calculated ΔΔ*G* and experimental *de* values; **(c)** Linear fitting between DeePEST-OS-calculated ΔΔ*G* and experimental *de* values.

17. Line 368 - If comparing timings, please use the same computing resources.

Clarification:

We greatly appreciate your valuable suggestion. To ensure a fair comparison of computational efficiency, we have re-evaluated the inference speeds of React-OT and DeePEST-OS-T1x on identical hardware (NVIDIA GeForce RTX 4060 Laptop GPU) and updated the comparative data accordingly in the revised manuscript.

Action taken:

- The sentence "*Among the 980 reactions that are successfully optimized by DeePEST-OS-T1x, React-OT, and DFT approaches (see* ***Tab. 2****), DeePEST-OS-T1x demonstrates significantly superior performance, exhibiting lower mean geometric RMSD (0.051 Å vs. 0.082 Å) and energy absolute error (0.690 kcal/mol*

*vs. 0.999 kcal/mol) compared to React-OT (Violin plot see **Fig. S2**). Notably, DeePEST-OS-T1x achieves dramatically higher computational efficiency, requiring only 3.4 core·s per reaction on average with NVIDIA GeForce RTX 4060 Laptop GPU for transition state optimization without additional energy computation steps while maintaining DFT-level accuracy. In contrast, React-OT necessitates GPU acceleration for transition state optimization (5.8 s per reaction on average on NVIDIA V100) but incurs substantial post-processing overhead due to DFT energy calculations (93.9 core·s per reaction on average).*" **has been revised to** "*Among the 995 reactions for which transition states were successfully optimized using DeePEST-OS-T1x, React-OT, and DFT (**Tab. 2**), DeePEST-OS-T1x demonstrates superior accuracy over React-OT, with a lower mean geometric RMSD (0.050 Å vs. 0.077 Å) and a smaller MAE of reaction energy barriers (0.692 kcal/mol vs. 1.038 kcal/mol) (see the violin plots in **Fig. S4**). Notably, DeePEST-OS-T1x was also more efficient, requiring an average of only 1.4 s per reaction for transition state optimization on an NVIDIA GeForce RTX 4060 Laptop GPU, whereas React-OT required 1.8 s per reaction. In addition, a distinct advantage of DeePEST-OS-T1x is its ability to simultaneously output a near-DFT-level predicted energy during transition state optimization. In contrast, React-OT focuses solely on geometry and does not provide energy outputs during transition state generation.*" (see **Lines 416-424** in the revised manuscript).

- **Tab. 2 has been revised** (see **Line 426** in the revised manuscript).

Tab. 2 Comparison of transition state geometric RMSDs, reaction energy barrier absolute errors, and computational costs between DeePEST-OS-T1x and React-OT models on the Transition1x external test set.

**Model**	**Geometric RMSD (Å)**		**Energy barrier absolute error (kcal/mol)**		**Computational cost (s)**
	Mean	Median	Mean	Median	Transition state optimization
React-OT	0.077	0.030	1.038	0.565	1.8
**DeePEST-OS-T1x**	**0.050**	**0.026**	**0.692**	**0.334**	**1.4**

18. Line 375 - "cannot be accelerated by GPU due to the inherent limitations of DFT." - I believe this is incorrect. There are GPU-accelerated DFT implementations. This should be either removed or updated.

Clarification:

Thank you so much for your professional comment. We have removed this statement in the revised manuscript.

Action taken:

- The sentence "*The calculation uses only a single CPU core (AMD Ryzen 9 7945HX) and cannot be accelerated by GPU due to the inherent limitations of DFT.*" **has been deleted**. (see **Line 428** in the revised manuscript).

19. Line 401 - most stable conformer? (not "modest stable")

Clarification:

Thank you for your careful reading. This typo has been corrected in the revised manuscript.

Action taken:

- The sentence "*...DFT optimizations to confirm the modest stable conformer...*" **has been revised to** "*...DFT optimizations to confirm the most stable conformer...*" (see **Line 454** in the revised manuscript).

20. A general comment is that the transferability of the trained ML models here is not strongly discussed or demonstrated. For example, in Line 345 onwards, reactions from transition1x are studied - but how similar are these to the original deepest-os training set? What if deepest-OS is applied to systems that are dissimilar to the training set? Is it possible to better quantify the transferability here, rather than just looking at a single additional example (in the final section about retrosynthesis)?

Clarification:

We appreciate your valuable suggestion to explicitly quantify the transferability of our MACE_deltaL model beyond a single case study. To address this, we established an "applicability domain" metric based on the Euclidean distance and 512-dimensional embedding space, which was derived from the embedding layer of the MACE_deltaL

model during inference. This metric allows us to filter out potentially unreliable predictions and prevent unreasonable extrapolation. Specifically, each sample in DORTS-1K calculated its Euclidean distance to the nearest neighbor in the training set (DORTS-9K) using the 512-dimensional embedding. The smaller the Euclidean distance to the nearest neighbor, the more similar the test set sample is to the training set. **Fig. 5(e)** presents the relationship between the nearest-neighbor Euclidean distance and the MAE of reaction energy barriers, demonstrating a clear positive correlation where the MAE increases as the nearest-neighbor distance increases. Notably, when the distance exceeds 0.7, the MAE of predicted reaction energy barriers surpasses the chemical accuracy threshold of 1 kcal/mol. Consequently, we defined 0.7 as the cutoff for the “applicability domain”, and reactions with distances within this range are considered to fall within the reliable prediction scope of our MACE_deltaL model.

Then, to address concerns regarding MACE_deltaL capacity to identify novel chemistry, we have supplemented an Out-Of-Distribution (OOD) test to evaluate the extrapolation capability of this model using reactions that are never seen during training and chemically distinct from known reaction categories in DORTS-9K (the train data for MACE_deltaL). The OOD dataset comprises 1,073 reactions selected from Transition1x, and the structural difference between DORTS-9K and Transition1x is visualized in the chemical space plot (**Fig. 3(c)**). This difference was further quantified using the “applicability domain” analysis. The analysis revealed that 1,038 reactions (~97%) in the Transition1x set exhibit the nearest-neighbor Euclidean distance greater than 0.7 relative to the training data (DORTS-9K), confirming that Transition1x represents a rigorous OOD for MACE_deltaL.

In this OOD test, a mean RMSD of 0.11 Å for transition state geometries and an MAE of 3.80 kcal/mol for reaction energy barriers were achieved by the MACE_deltaL model (**Fig. 5(f)**). While these metrics indicate a modest decline in accuracy relative to the performance on the DORTS-1K test set—attributable to the notable structural differences between the Transition1x and DORTS-9K datasets that pose challenges for extrapolation—the predictive accuracy of our MACE_deltaL model remains highly competitive with that of the OA-ReactDiff model (a mean geometric RMSD of 0.129 Å and an MAE of 4.4 kcal/mol for reaction energy barriers) [Nat. Comput. Sci., 3, 1045-1055, 2023]. Note that OA-ReactDiff was specifically trained on the Transition1x dataset, whereas our MACE_deltaL model has not trained on it. Collectively, these results demonstrate the robust extrapolation ability of our MACE_deltaL model.

Action taken:

- The paragraph "*To quantify the transferability of MACE_deltaL and mitigate the risks of unreliable extrapolation, an "applicability domain" metric was established based on the Euclidean distance and a 512-dimensional embedding space, which was derived from the embedding layer of MACE_deltaL during inference. Utilizing this metric allows potentially unreliable predictions to be filtered out and unreasonable extrapolation to be prevented. Specifically, the Euclidean distance to the nearest neighbor in the training set (DORTS-9K) was calculated for each sample in the DORTS-1K set using the 512-dimensional embeddings. A smaller Euclidean distance to the nearest neighbor indicates a higher similarity between the test sample and the training set. The relationship between the nearest-neighbor Euclidean distance and the MAE of reaction energy barriers is presented in **Fig. 5(e)**, revealing a clear positive correlation where the MAE rises with increasing nearest-neighbor Euclidean distance. Notably, when the nearest-neighbor Euclidean distance exceeds 0.7 Å, the MAE of predicted reaction energy barriers surpasses the chemical accuracy threshold of 1 kcal/mol. Consequently, a value of 0.7 was defined as the cutoff for the "applicability domain". Reactions with nearest-neighbor Euclidean distances within this threshold are considered to fall within the reliable prediction scope of the MACE_deltaL model.*" **has been added** (see **Lines 360-372** in the revised manuscript).

- The paragraph "*To validate the extrapolation ability of the MACE_deltaL model, it is essential to test its performance on Out-Of-Distribution (OOD) reactions. As illustrated in the chemical space in **Fig. 3(c)**, the reactions in the Transition1x (red dots) and DORTS-9K training set (blue dots) exhibit limited overlap and occupy distinct regions, indicating reaction dissimilarity. This dissimilarity was further quantified using the "applicability domain" analysis, revealing that 1,038 reactions (~97%) in the Transition1x set exhibited a nearest-neighbor Euclidean distance greater than 0.7 relative to the training data in DORTS-9K. This confirms that Transition1x serves as a representative OOD dataset for testing MACE_deltaL. In this rigorous OOD test, a mean RMSD of 0.11 Å for transition state geometries and an MAE of 3.80 kcal/mol for reaction energy barriers were achieved by the MACE_deltaL model (**Fig. 5(f)**). While these metrics indicate a modest decline in accuracy relative to the performance on the DORTS-1K test set—attributable to the notable structural differences between the Transition1x and DORTS-9K*

datasets that pose challenges for extrapolation—the predictive accuracy of our MACE_deltaL model remains highly competitive with that of the OA-ReactDiff model (a mean geometric RMSD of 0.129 Å and an MAE of 4.4 kcal/mol for reaction energy barriers)[55], which was specifically trained on the Transition1x dataset. These results demonstrate the robust extrapolation ability of the MACE_deltaL model." **has been added** (see **Lines 373-386** in the revised manuscript).

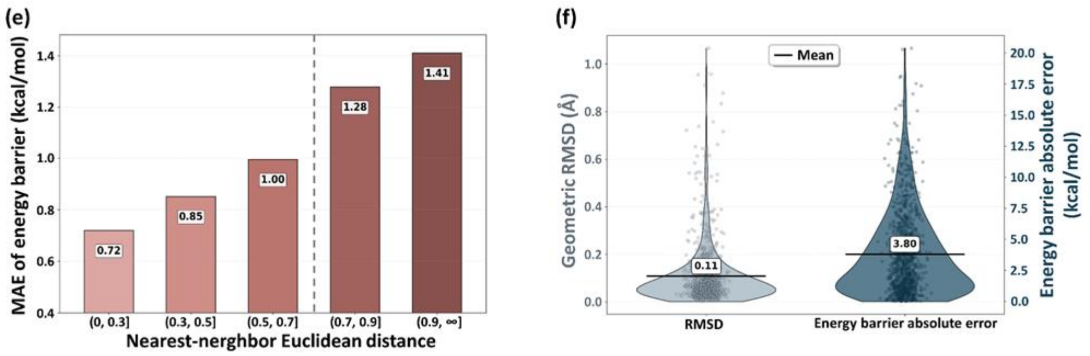

Fig. 5 Accuracy and computational cost evaluations based on the test set. (e) Bar chart illustrating the trend between the nearest-neighbor Euclidean distance and the MAE of predicted reaction energy barriers for DORTS-1K (the grey dashed line at a distance of 0.7 marks the defined cutoff for the applicability domain of MACE_deltaL. **(f)** Violin plot depicting the distribution of transition state geometric RMSDs and absolute deviations in predicted reaction energy barriers for the 1,073 reactions in Transition1x by the MACE_deltaL model.

21. Again, Figure 7 only represents a comparison of the ML model against DFT. What about comparison to experimentally-determined barriers?

Clarification:

We greatly appreciate your valuable suggestion to validate the retrosynthesis case study with experimental data. Despite an exhaustive literature search, we failed to find reported experimental kinetic data or energy barriers for the specific reactions generated in the retrosynthesis case study. Nevertheless, as demonstrated in our response to **Question [16]** regarding the experimental validation of Diels-Alder reactions, we have verified that our DeePEST-OS model achieves a reasonable agreement with experimental observations, achieving an R^2 of 0.76 against experimental diastereomeric excess (*de*) values. This reliability depends on both the comprehensiveness of conformational isomer sampling and the accuracy of the chosen DFT method. Note that

we followed to this identical protocol in the retrosynthesis case study by explicitly considering conformational isomerism and employing the same DeePEST-OS model. Consequently, we conclude that the retrosynthesis results are qualitatively reliable.

Action taken:

- The part "*Experimental validation of endo and exo diastereoselectivity in Diels-Alder reactions*" **has been added** (see **Line 517** in the revised manuscript).

- The paragraph "*To evaluate the error between DeePEST-OS predictions and experimental data, the endo/exo diastereoselectivity of the Diels-Alder reaction was tested as a case study. Experimental diastereomeric excess (de) values were sourced from the literature[60], and theoretically, de is fundamentally correlated with the Gibbs free energy barrier difference (ΔΔG) between the endo and exo insertions. To predict these ΔΔG values, DFT calculations were employed at the ωB97M-V/def2-TZVP level, as this DFT level maintains consistency with the DeePEST-OS training database (DORTS-9K) and exhibits excellence in predicting thermodynamic properties[52]. Furthermore, conformational isomerism was explicitly considered, since it has been shown to improve the quantitative agreement between theoretical calculations and experimental observations[61,62]. After evaluating the ΔΔG values for 11 distinct reactants, a least-squares linear regression was performed to correlate the DFT-calculated values with the experimental de values, as depicted in* ***Fig. 8(b)****. The resulting coefficient of determination (R^2) was 0.82, demonstrating that this benchmark DFT methodology has the capacity to provide reliable predictions of experimental de trends for the endo/exo diastereoselectivity of the Diels-Alder reaction.*" **has been added** (see **Lines 518-529** in the revised manuscript).

- The paragraph "*To further evaluate DeePEST-OS,* ***Fig. 8(c)*** *displays the correlation between DeePEST-OS-predicted ΔΔG values and experimental de values. The resulting R^2 of 0.76 demonstrated a reasonably strong quantitative correlation. Although this R^2 was slightly lower than that achieved by the DFT method, DeePEST-OS preserves the DFT-inherent predictive capacity for de values to facilitate experimental exploration.*" **has been added** (see **Lines 530-533** in the revised manuscript).

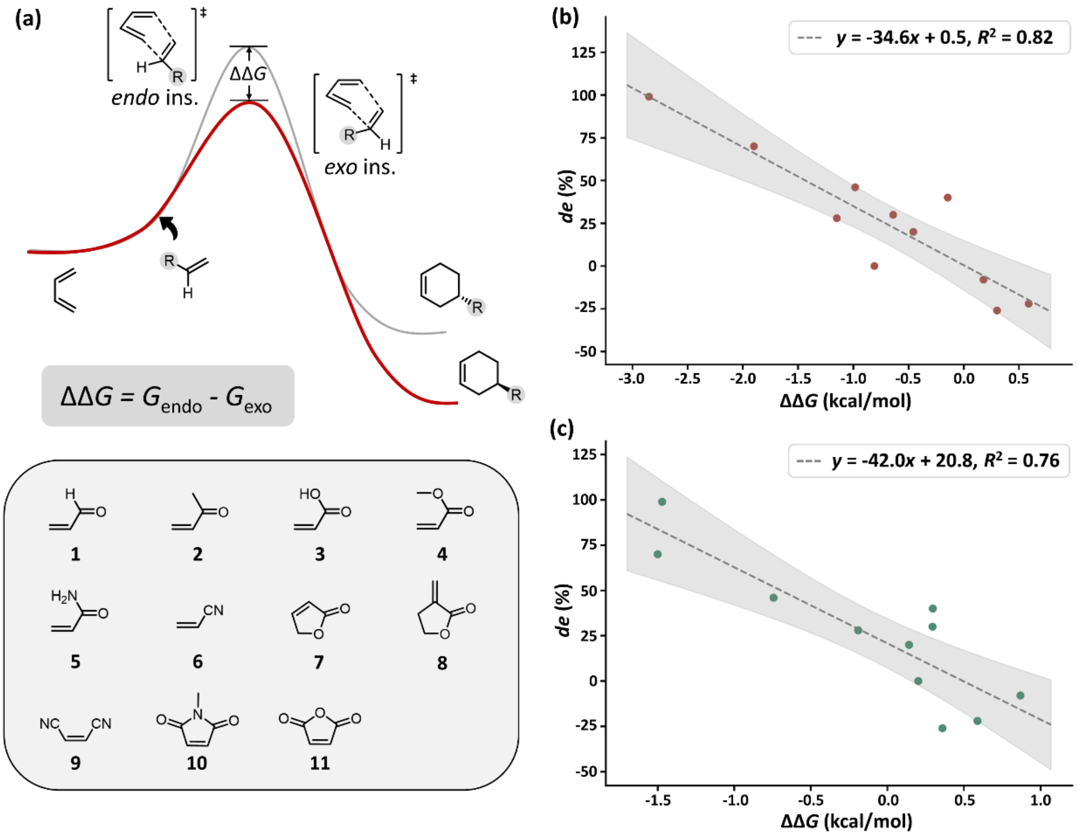

Fig. 8 Linear fitting between ΔΔ*G* and *de* values for endo and exo insertions of different reactants in Diels-Alder reactions. (a) ΔΔ*G* definition and reactant structures; **(b)** Linear fitting between DFT-calculated ΔΔ*G* and experimental *de* values; **(c)** Linear fitting between DeePEST-OS-calculated ΔΔ*G* and experimental *de* values.

22. Line 174, Figure 2b: If the y-axis of this figure is intended to be energy and the x-axis is the reaction coordinate, it is unclear what the two paths passing through F_max_rev and F_max_forw are, and why they each start/end at the energy of the reactants/products but the reaction coordinate position of the TS. Also, while I understand that these names correspond to the two conformations with maximum force, they should be made clearer for less technically inclined audiences.

Clarification:

We sincerely appreciate your constructive suggestions. In **Fig. 2(b)**, five critical conformations are selected along the IRC pathway. The two endpoints represent the reactant and product structures, and the maximum point corresponds to the transition state. The remaining two conformations are identified as those with the steepest slopes along the IRC pathway—specifically, the points where the absolute value of the first

derivative of energy with respect to the reaction coordinate reaches its maximum. Since this first derivative represents the force acting on the system, these two conformations are denoted as F_max_rev (reverse direction) and F_max_forw (forward direction). To improve clarity for readers, we have supplemented relevant descriptions in the revised manuscript.

Action taken:

- The sentence "*Then, five critical conformations were selected along the IRC pathway. The two endpoints represented the reactant and product structures, and the maximum point corresponded to the transition state. The remaining two conformations were identified as those with the steepest slopes along the IRC pathway—specifically, the points where the absolute value of the first derivative of energy with respect to the reaction coordinate reaches its maximum. Since this first derivative represents the force acting on the system, these two conformations were denoted as F_max_rev (reverse direction) and F_max_forw (forward direction).*" **has been added** (see **Lines 174-179** in the revised manuscript).

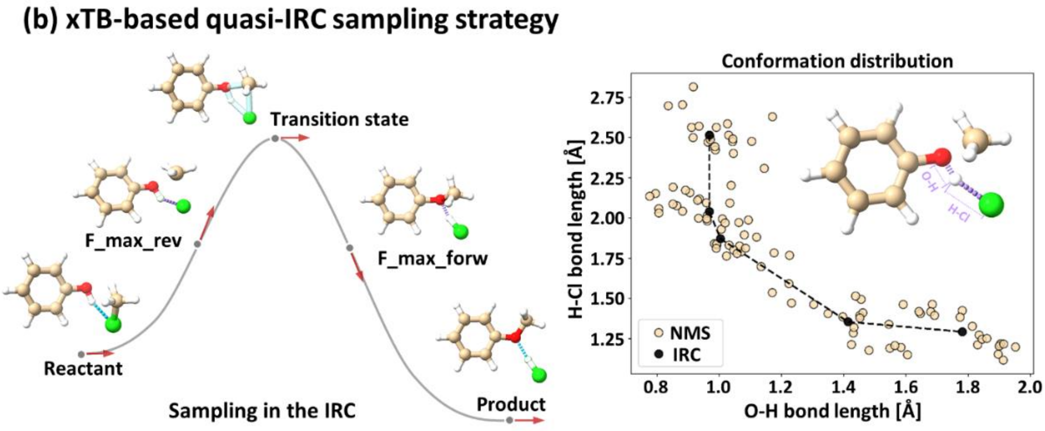

Fig. 2 The generation of the DORTS database. (b) Five selected critical conformations along the IRC pathway (left figure) and the NMS-sampled conformations (yellow dots) for the five selected critical conformations (black dots) along the IRC pathway during phenol etherification (right figure).

23. Line 188: The authors say that the reactions in DORTS-9K were sampled randomly from the wider DORTS dataset, rather than being stratified by reaction classes to ensure there is no bias towards/against any particular reaction types in DORTS-9K. Given the authors already have reaction class labels from the generation of DORTS, do they anticipate that random sampling could leave out or underemphasise any

reaction classes when constructing DORTS-9K? To test this, they could check for a uniform distribution of the 294 reaction templates in the reactions that made it into DORTS-9K.

Furthermore, given that the full DORTS dataset already contains 7.5 million conformations from across the whole range of input reactions, why did the authors not sample (with stratification by reaction class) these conformers directly? Surely this would yield a more chemically diverse dataset than DORTS-9K, given that conformers from all 74,837 reactions are present rather than only containing conformers from the 9,017 reactions selected in DORTS-9K?

Clarification:

We sincerely appreciate your insightful suggestions regarding the sampling strategy of the DORTS-9K dataset.

For the first question concerning potential bias in random sampling, we have plotted a scatter diagram illustrating the number of reactions corresponding to each reaction template in both the DORTS-9K and full DORTS dataset (**Fig. S1**). As shown in the figure, the two datasets exhibit a highly consistent trend in the reaction count across different templates, which demonstrates that the random sampling strategy achieved a satisfactory performance without introducing obvious bias toward specific reaction templates. Importantly, DORTS-9K still contains all reaction templates without omission after random sampling. Furthermore, the number of reactions generated by different reaction templates varies significantly instead of showing a uniform distribution. This non-uniformity originates from the CAMD generation rules rather than from the sampling step, because different reaction templates inherently differ in their designable branches (*). As illustrated in **Fig. S1**, the two example templates contain 1 and 5 branches, respectively. A higher number of branches increases the combinatorial space for group attachment, thus yielding a greater variety of possible reactions. To achieve a reaction count comparable to that of a five-branched template using a single-branched template, one would need to form excessively long chains—composed of numerous groups—that are subsequently attached to the single branch. However, groups at the distal ends of such long chains generally exhibit limited electronic effect on the reactive site. Consequently, generating a large number of such reaction samples (which exert similar effects on the reactive site) reduces the challenge inherent in extrapolation tasks, thus leading to artificially inflated prediction accuracy.

Regarding the second question about direct stratified sampling (by reaction class) of conformers from the full DORTS dataset, while this approach would expose the model to more reactions, it would also reduce the extrapolation challenge. For example, if the training set is altered to DORTS-74K (maintaining the original sample size of 874,176), randomly selecting samples from the remaining DORTS data for extrapolation testing would reveal that all chosen samples correspond to reactions already present in the training set. This overlap reduces the extrapolation challenge, artificially inflating prediction accuracy and failing to reflect real-world generalization. To ensure a rigorous evaluation of true extrapolation ability, we instead train the model on DORTS-9K and validate it using DORTS-1K, which contains entirely novel reactions never encountered during training. This design guarantees that the assessment measures how well the model generalizes to unseen reactions, thus providing a reliable benchmark for its practical utility.

Action taken:

➢ The sentence "***Fig. S1*** *illustrates the distribution of reaction counts for DORTS-9K and DORTS across 255 reaction templates*" **has been added** (see **Line 202** in the revised manuscript and **Fig. S1** in the revised supporting information).

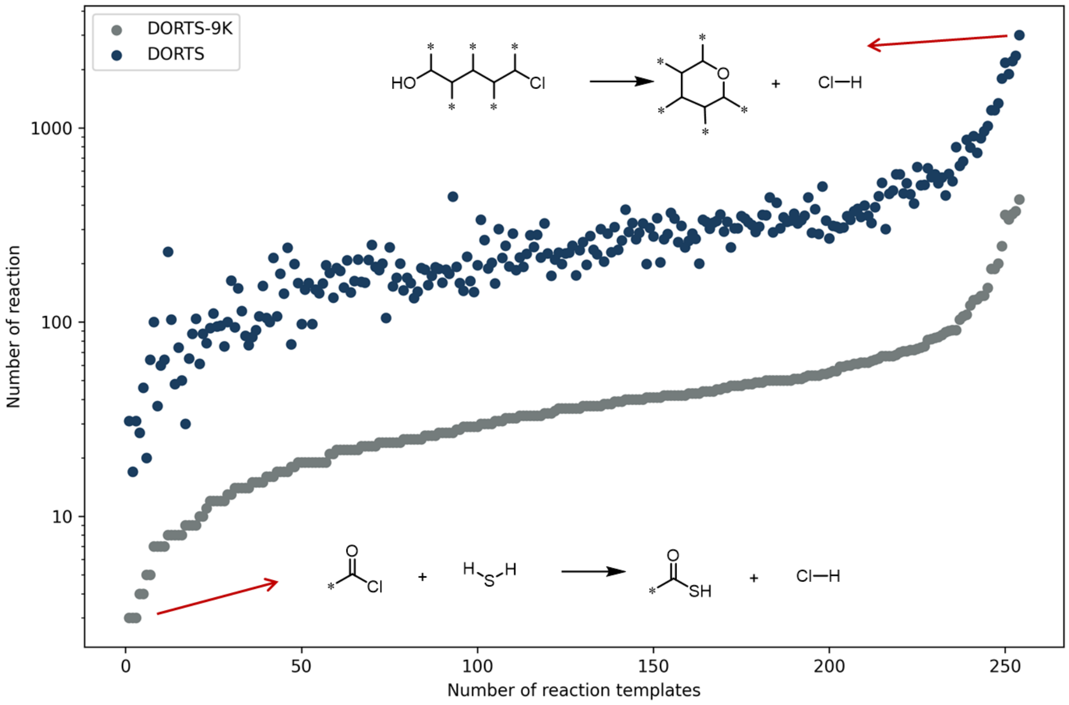

Fig. S1 Distribution of reaction counts for DORTS-9K and DORTS across 255 reaction templates.

24. Line 215, Figure 3b: It appears that many of the transition states from the Transition1x dataset fall outside of the latent chemical space that DORTS encompasses, evidenced by a lack of points around many of the red regions. Does this indicate that models trained on DORTS-9K would fail to accurately predict the reaction paths of the reactions from Transition1x, or is it an artefact of how t-SNE was run on this data? Either way, this should be clarified in the text.

Clarification:

We sincerely appreciate your careful observation and insightful comments. As you rightly noted, the chemical space of Transition1x does not overlap with that of DORTS, indicating that the reactions in Transition1x are chemically distinct from those in DORTS. To quantify this difference, we computed the nearest-neighbor Euclidean distance metric between Transition1x and DORTS. After analyzing this metric, we confirmed that Transition1x is Out-Of-Distribution (OOD) relative to DORTS. More details of the nearest-neighbor Euclidean distance metric and its analysis process can be found in **Question [20]**.

Then, we utilized the Transition1x dataset for the OOD validation of the DeePEST-OS model to comprehensively evaluate its generalization capability. In this OOD test, a mean RMSD of 0.11 Å for transition state geometries and an MAE of 3.80 kcal/mol for reaction energy barriers were achieved by the MACE_deltaL model (**Fig. 5(f)**). While these metrics indicate a modest decline in accuracy relative to the performance on the DORTS-1K test set — attributable to the notable structural differences between the Transition1x and DORTS-9K datasets that pose challenges for extrapolation—the predictive accuracy of our MACE_deltaL model remains highly competitive with that of the OA-ReactDiff model (a mean geometric RMSD of 0.129 Å and an MAE of 4.4 kcal/mol for reaction energy barriers) [Nat. Comput. Sci., 3, 1045-1055, 2023]. Note that OA-ReactDiff was specifically trained on the Transition1x dataset, whereas our MACE_deltaL model has not trained on it. Collectively, these results demonstrate the robust extrapolation ability of our MACE_deltaL model.

Action taken:

➢ The paragraph "*To quantify the transferability of MACE_deltaL and mitigate the risks of unreliable extrapolation, an "applicability domain" metric was established based on the Euclidean distance and a 512-dimensional embedding space, which was derived from the embedding layer of MACE_deltaL during inference.*

Utilizing this metric allows potentially unreliable predictions to be filtered out and unreasonable extrapolation to be prevented. Specifically, the Euclidean distance to the nearest neighbor in the training set (DORTS-9K) was calculated for each sample in the DORTS-1K set using the 512-dimensional embeddings. A smaller Euclidean distance to the nearest neighbor indicates a higher similarity between the test sample and the training set. The relationship between the nearest-neighbor Euclidean distance and the MAE of reaction energy barriers is presented in ***Fig. 5(e)****, revealing a clear positive correlation where the MAE rises with increasing nearest-neighbor Euclidean distance. Notably, when the nearest-neighbor Euclidean distance exceeds 0.7 Å, the MAE of predicted reaction energy barriers surpasses the chemical accuracy threshold of 1 kcal/mol. Consequently, a value of 0.7 was defined as the cutoff for the "applicability domain". Reactions with nearest-neighbor Euclidean distances within this threshold are considered to fall within the reliable prediction scope of the MACE_deltaL model.*" **has been added** (see **Lines 360-372** in the revised manuscript).

- The paragraph "*To validate the extrapolation ability of the MACE_deltaL model, it is essential to test its performance on Out-Of-Distribution (OOD) reactions. As illustrated in the chemical space in* ***Fig. 3(c)****, the reactions in the Transition1x (red dots) and DORTS-9K training set (blue dots) exhibit limited overlap and occupy distinct regions, indicating reaction dissimilarity. This dissimilarity was further quantified using the "applicability domain" analysis, revealing that 1,038 reactions (~97%) in the Transition1x set exhibited a nearest-neighbor Euclidean distance greater than 0.7 relative to the training data in DORTS-9K. This confirms that Transition1x serves as a representative OOD dataset for testing MACE_deltaL. In this rigorous OOD test, a mean RMSD of 0.11 Å for transition state geometries and an MAE of 3.80 kcal/mol for reaction energy barriers were achieved by the MACE_deltaL model (****Fig. 5(f)****). While these metrics indicate a modest decline in accuracy relative to the performance on the DORTS-1K test set—attributable to the notable structural differences between the Transition1x and DORTS-9K datasets that pose challenges for extrapolation—the predictive accuracy of our MACE_deltaL model remains highly competitive with that of the OA-ReactDiff model (a mean geometric RMSD of 0.129 Å and an MAE of 4.4 kcal/mol for reaction energy barriers)[55], which was specifically trained on the Transition1x dataset. These results demonstrate the robust extrapolation ability of the*

MACE_deltaL model." **has been added** (see **Lines 373-386** in the revised manuscript).

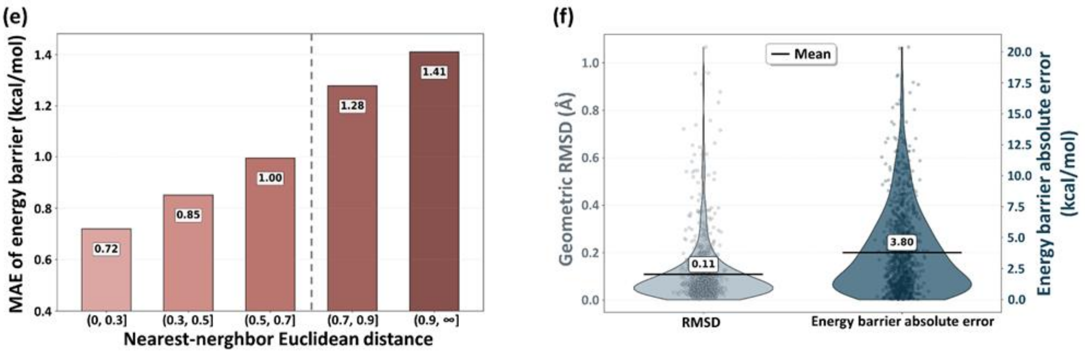

Fig. 5 Accuracy and computational cost evaluations based on the test set. (e) Bar chart illustrating the trend between the nearest-neighbor Euclidean distance and the MAE of predicted reaction energy barriers for DORTS-1K (the grey dashed line at a distance of 0.7 marks the defined cutoff for the applicability domain of MACE_deltaL. **(f)** Violin plot depicting the distribution of transition state geometric RMSDs and absolute deviations in predicted reaction energy barriers for the 1,073 reactions in Transition1x by the MACE_deltaL model.

25. Line 215, Figure 3c: The latent chemical space shown in this figure is not the same as in Figure 3b, so the two are not directly comparable. I assume this is because a different t-SNE map was constructed without the reactions from Transition1x? However, this means the two figures cannot be compared. Instead, the same t-SNE map could be used for both figures, but with the Transition1x points excluded from Figure 3c.

Clarification:

We sincerely appreciate your insightful comment regarding the consistency of the latent chemical space visualization. As you correctly pointed out, the original **Fig. 3(c)** aimed to visualize the continuity of energy distribution within the DORTS dataset. We acknowledge that using different t-SNE projections hinders a direct comparison between the geometry (**Fig. 3(b)**) and energy distributions (**Fig. 3(c)**). Following your constructive suggestion, we have revised **Fig. 3(c)** by adopting the same chemical space coordinates as in **Fig. 3(b)**. Specifically, we excluded the Transition1x data points and plotted DORTS data points at their corresponding positions in **Fig. 3(b)**, with colors representing energy values. This modification enables a direct comparison between the

geometry distribution in **Fig. 3(b)** and the energy distribution in **Fig. 3(c)**, providing a more consistent and intuitive representation of the dataset characteristics.

Action taken:

➢ **Fig. 3** has been revised (see **Line 240** in the revised manuscript).

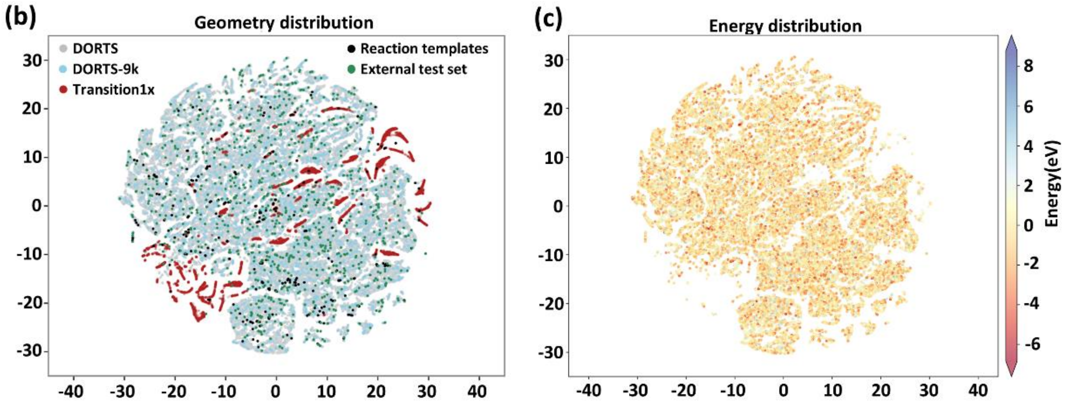

Fig. 3 Data analysis for the DORTS database. (b) Chemical space mapped using the ACSFs descriptors and t-SNE method (gray points represent the DORTS transition states, blue points represent the DORTS-9K transition states that are used to train DeePEST-OS, red points represent the Transition1x transition states, black point represent the initial 255 reaction templates, green point represent DORTS-1K comprising 1,000 novel reactions not included in DORTS-9K). **(c)** Chemical space of the DORTS transition states with colors representing energy values (the plotted energy values are obtained by subtracting the reference energies from the DFT-calculated energies).

26. Line 385: The authors state that test cases for the DeePEST-OS/React-OT benchmark are detailed in the supporting information, but this is not present.

Clarification:

We sincerely apologize for omitting the relevant data in our initial submission. The benchmark details for the DeePEST-OS/React-OT test cases are now available at https://github.com/kaipai-ren/DeePEST-OS/tree/main/dataset/cross-dataset_validation_of_DeePEST-OS-T1x.

Action taken:

- ➢ The sentence "*<u>All source data in this study are available at https://github.com/kaipai-ren/DeePEST-OS/tree/main.</u>*" **has been added** (see **Line 724** in the revised manuscript).

27. While it is clear that DeePEST-OS excels at prediction of reaction energetics, when performing chemical kinetics simulations this is often only half of the problem – additionally, the thermodynamics of reactions must be considered. In conventional transition state theory, this requires accurate vibrational energies of both reactants and transition states. While I appreciate that benchmarking this would be a significant undertaking, it would be valuable and highly relevant to the discussion of the simulation of chemical reaction networks.

Clarification:

Thank you for this important comment and we fully agree that, for chemical kinetics simulations within transition state theory, accurate vibrational energies of both reactants and transition states are essential. In response, we have revised the section "Kinetic prediction of multi-step organic synthesis driven by DeePEST-OS" by replacing the electronic energies (E) with Gibbs free energies (G). Geometry and energy barrier error analyses (**Fig. 7(c)**) reveal that DeePEST-OS achieves lower mean geometric RMSD (0.16 Å for intermediates/transition states) and MAE of Gibbs free energy barriers (1.18 kcal/mol) compared to GFN2-xTB (0.43 Å and 10.00 kcal/mol, respectively), confirming the precision of DeePEST-OS in multi-step barrier prediction.

In addition, we have added a new section entitled "Experimental validation of endo and exo diastereoselectivity in Diels-Alder reactions", where Gibbs free energies are employed to benchmark the experimentally observed selectivity. **Fig. 8(c)** displays the correlation between DeePEST-OS-predicted $\Delta\Delta G$ values and experimental *de* values. The resulting R^2 of 0.76 demonstrates a reasonably strong quantitative correlation.

Considering that electronic energy (E) allows fair and consistent comparisons with other models such as OA-ReactDiff and React-OT, we prioritized comparisons based on electronic energies to demonstrate the model accuracy and to enable a transparent error analysis in the large-scale benchmarking part of the manuscript. In contrast, for the sections where reaction kinetics were studied (including the newly added experimental selectivity validation), we have followed your suggestion and used Gibbs free energies.

Action taken:

➢ The sentence "*The DeePEST-OS model is then employed to identify the most stable intermediate and transition state conformers, along with calculating energy barriers for elementary reactions.*" **has been revised to** "*DeePEST-OS was then employed to identify the most stable intermediate and transition state conformers, along with calculating Gibbs free energy barriers for elementary reactions.*" (see **Line 492** in the revised manuscript).

➢ The sentence "*Structural and energy barrier error analyses (**Fig. 7(c)**) reveal that DeePEST-OS achieves significantly lower mean geometric RMSD (0.16 Å for intermediates/transition states) and energy barrier MAE (0.43 kcal/mol) compared to GFN2-xTB (1.69 Å and 11.52 kcal/mol, respectively), confirming the precision of DeePEST-OS in multi-step barrier prediction.*" **has been revised to** "*Geometry and energy barrier error analyses (**Fig. 7(c)**) reveal that DeePEST-OS achieved lower mean geometric RMSD (0.16 Å for intermediates/transition states) and MAE of Gibbs free energy barriers (1.18 kcal/mol) compared to GFN2-xTB (0.43 Å and 10.00 kcal/mol, respectively), confirming the precision of DeePEST-OS in multi-step barrier prediction.*" (see **Lines 497-500** in the revised manuscript).

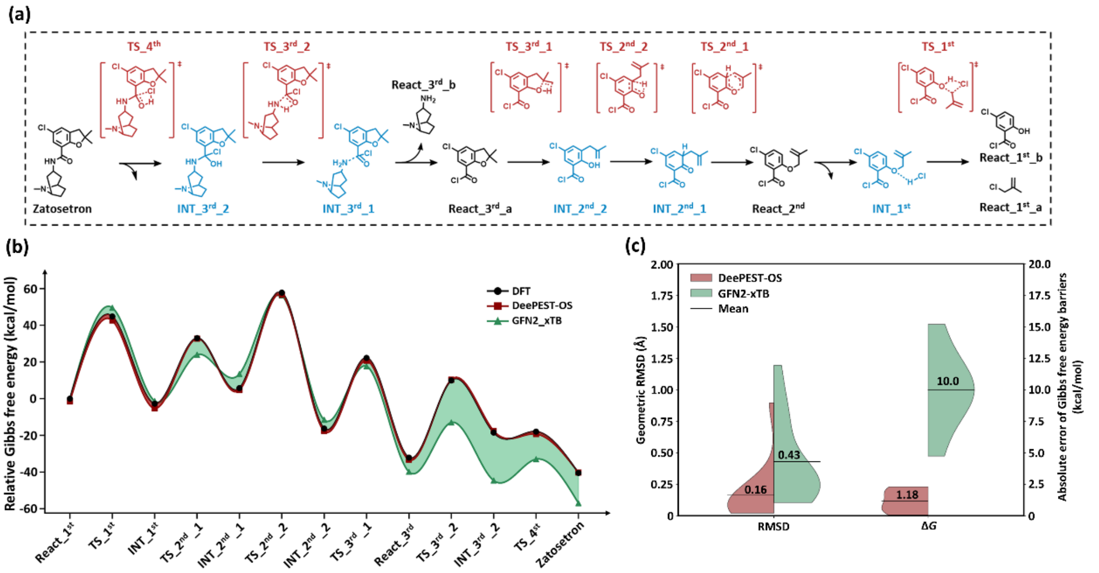

Fig. 7 Kinetic prediction of multi-step organic synthesis for Zatosetron compound using DeePEST-OS. (a) The optimal reaction pathway for Zatosetron synthesis co-constructed by retrosynthesis software RetroSynX and initial guess of

transition state generator GENiniTS-RS (black structures represent the synthesis pathway designed by RetroSynX, red structures indicate transition state conformations generated by GENiniTS-RS, and blue structures correspond to intermediate conformations along the IRC pathways). **(b)** Multi-step reaction potential energy profiles of Zatosetron synthesis calculated by DFT, DeePEST-OS, and GFN2-xTB (black curve: DFT benchmark; red curve: DeePEST-OS prediction; green curve: GFN2-xTB prediction). **(c)** Violin plots comparing RMSD of transition state/intermediate geometries and absolute error of Gibbs free energy barriers between DeePEST-OS and GFN2-xTB, with DFT calculations as reference.

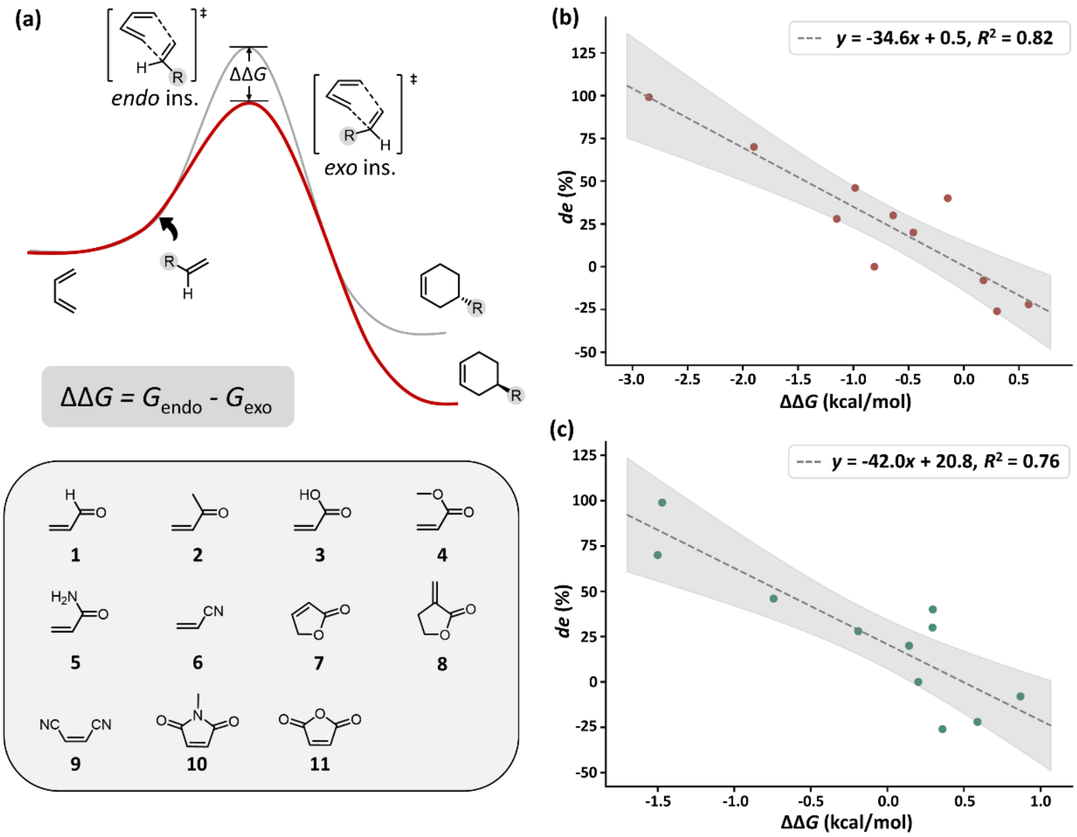

Fig. 8 Linear fitting between ΔΔ*G* and *de* values for endo and exo insertions of different reactants in Diels-Alder reactions. (a) ΔΔ*G* definition and reactant structures; **(b)** Linear fitting between DFT-calculated ΔΔ*G* and experimental *de* values; **(c)** Linear fitting between DeePEST-OS-calculated ΔΔ*G* and experimental *de* values.

28. The additional dataset zip file that is being provided with the paper has no documentation and is not mentioned in the text. It appears to contain XYZ geometries

for Table 2, Figure 6 and Figure 7. This is useful and should be included, it just needs mentioning in e.g. the Data Availability section.

Clarification:

We sincerely apologize for omitting documentation for the supplementary dataset zip file and failing to mention it in the original manuscript. To enhance accessibility and clarity, we have moved all source data to a GitHub repository instead of uploading a zip file. The repository includes a README file detailing the dataset contents. We have also updated the Data Availability and Reproducibility Statement to confirm that all source data in this study are available at https://github.com/kaipai-ren/DeePEST-OS/tree/main/.

Action taken:

- The sentence "*All source data in this study are available at https://github.com/kaipai-ren/DeePEST-OS/tree/main.*" **has been added** (see **Line 724** in the revised manuscript).

Response to the Reviewers

Dear Editor,

Thank you for your letter and the reviewers' comments on our manuscript submitted to *Nature Communications* (Submission title: Reactive Machine Learning Potential for Accelerating Transition State Search in Organic Synthesis; Manuscript ID: NCOMMS-25-69131A). We are delighted to hear that our manuscript can be published in principle.

Following the editorial requests and the remaining comments from the reviewers, we have further refined the manuscript and the supporting materials. Specifically, we have supplemented the missing axis labels in Figure 3 and created a permanent DOI for our code and models to ensure long-term accessibility.

A point-by-point response to the final editorial and reviewer comments is provided below.

Yours sincerely,

Qilei Liu

Address: State Key Laboratory of Fine Chemicals, Frontiers Science Center for Smart Materials Oriented Chemical Engineering, Department of Pharmaceutical Sciences, Institute of Chemical Process Systems Engineering, School of Chemical Engineering, Dalian University of Technology, Dalian 116024, China

Email: liuqilei@dlut.edu.cn

Editorial Requests

1. Featured Image Suggestion

Response:

We have prepared a high-resolution featured image (1200 × 675 pixels, RGB). This image has been uploaded with the title “featured image”.

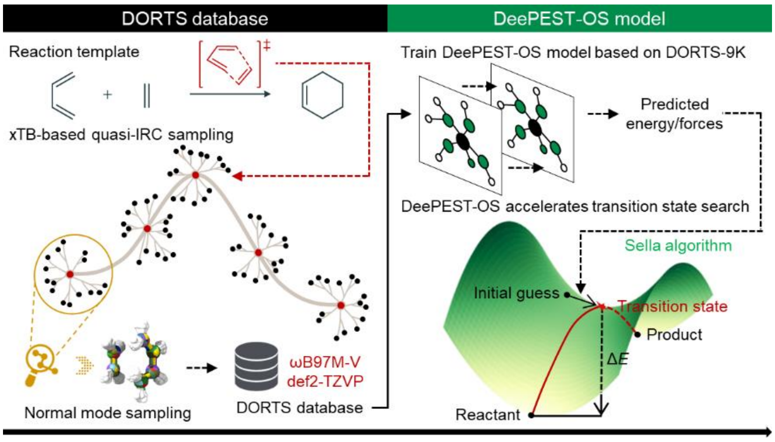

2. ORCID Linking

Response:

The corresponding author (Dr. Qilei Liu) has linked his ORCID identifier (0000-0002-3879-1827) to his account on the Manuscript Tracking System (MTS) as requested.

3. Data and Code Availability

Response:

We have updated the "Code Availability" section. In response to Reviewer 3's suggestion regarding long-term availability, the DeePEST-OS and inference codes have been deposited on Zenodo with a permanent DOI: 10.5281/zenodo.19305799.

Reviewer 1:

The authors have done substantial work in this revision for the comments from other reviewers and myself. All my comments are addressed. I really like their efforts on pushing datasets towards elements other than CNOH, which is much needed in the field towards real impactful applications.

Response:

We sincerely thank you for your positive recognition of our work and for the encouraging feedback regarding the expansion of the chemical space in reactive datasets.

Reviewer 2:

I co-reviewed this manuscript with one of the reviewers who provided the listed reports. This is part of the Nature Communications initiative to facilitate training in peer review and to provide appropriate recognition for Early Career Researchers who co-review manuscripts.

Response:

We would like to thank you for your participation in the peer-review process of our manuscript as part of the Nature Communications co-review initiative. We appreciate the time and effort you dedicated to reviewing our work.

Reviewer 3:

The authors have done a good job in addressing my previous comments. I do not have any further major comments to add. The work should be a useful addition to the growing number of ML models for predicting reaction properties such as activation energies.

The further analysis of sampled chemical reaction space is very useful, as is the further performance and transferability analysis performed for alternative datasets. The new sections and additions help set the context for the work, and provide further insight into the range of applicability of the ML model developed here.

A minor comment is that some of the figures are missing labels on their axes - for example, the new Figure 3 is missing axis labels.

While I have not fully cloned and tested the uploaded software, I have had a look at the GitHub repository and it appears to contains the relevant information and documentation that one would expect (at least on the front page). A further comment here is that some sort of permanent DOI (or deposit somewhere with this facility) would help ensure that this software and data remains available in the longer term - as it is, this software and data appears to be associated with one of the authors personal GitHub repository.

Response:

We sincerely thank you for the positive evaluation and for the constructive suggestions to further polish our manuscript.

Regarding the axis labels, we apologize for the oversight in the previous version. We have now thoroughly updated Figure 3 to ensure all axes are clearly labeled and scientifically rigorous:

1) For the t-SNE maps in Figure 3(b) and Figure 3(c), we have added "t-SNE 1" and "t-SNE 2" as axis labels.

2) For the violin plots in Figure 3(d) and Figure 3(e), we have explicitly added "Element type" as the *x*-axis label.

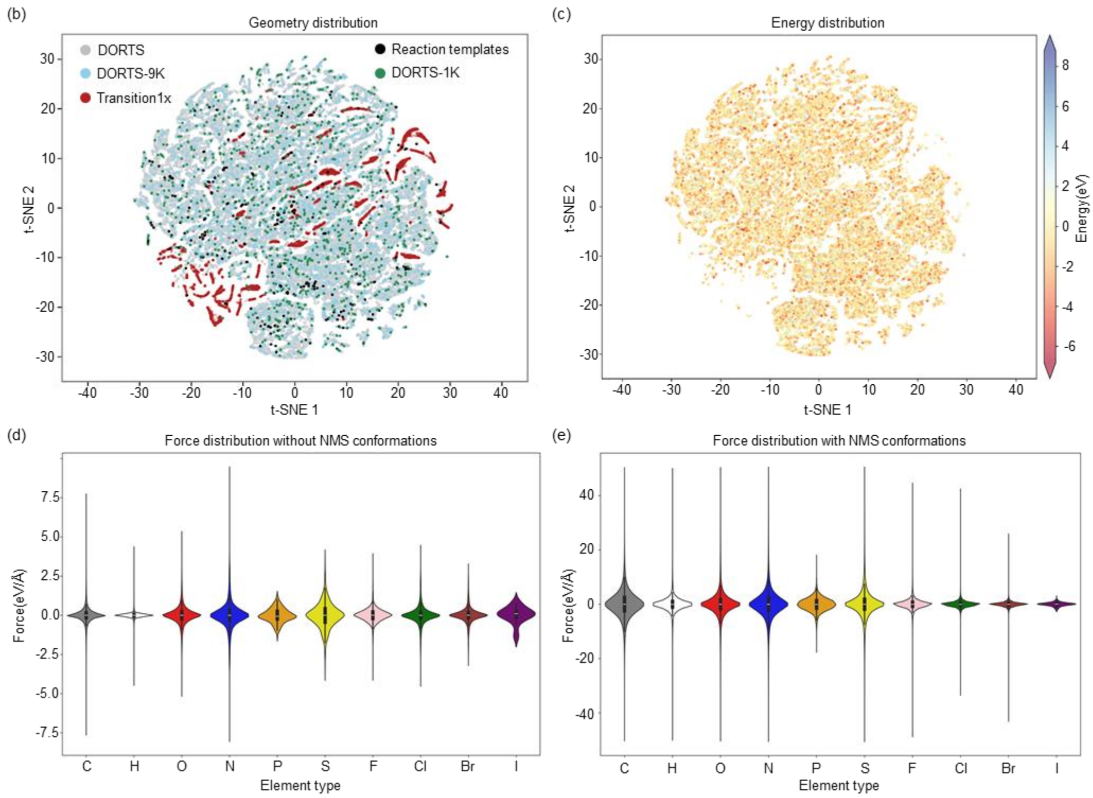

Fig. 3 Data analysis for the DORTS database. (a) Distribution of total atoms and heavy atoms per reaction in the DORTS and Transition1x databases (gray bars represent total atom counts in DORTS; white bars represent heavy atom counts in DORTS; red bars represent total atom counts in Transition1x; pink bars represent heavy atom counts in Transition1x). (b) Chemical space mapped using the ACSFs descriptors and t-SNE method (gray points represent the DORTS transition states; blue points represent the DORTS-9K transition states that are used to train DeePEST-OS; red points represent the Transition1x transition states; black points represent the initial 255 reaction templates; green points represent DORTS-1K comprising 1,000 reactions not included in DORTS-9K). (c) Chemical space of the DORTS transition states with colors representing energy values (the plotted energy values are obtained by subtracting the reference energies from the DFT-calculated energies). (d) Violin diagram of atomic force distributions considering only IRC conformations. (e) Violin diagram of atomic force distributions considering both IRC and NMS conformations. Source data are provided as a Source Data file.

Furthermore, we fully agree with the importance of data persistence and long-term accessibility. As suggested, the DeePEST-OS and inference codes have been deposited on Zenodo with a permanent DOI: 10.5281/zenodo.19305799. Correspondingly, we have updated this citation in the "Code Availability" section and the "References" section to ensure the codes and models are properly archived and accessible to the community.
